# Mechanistic models project bird invasions with accuracy

**DOI:** 10.1038/s41467-023-38329-4

**Published:** 2023-05-02

**Authors:** Diederik Strubbe, Laura Jiménez, A. Márcia Barbosa, Amy J. S. Davis, Luc Lens, Carsten Rahbek

**Affiliations:** 1 Terrestrial Ecology Unit (TEREC), Department of Biology, Ghent University, K.L. Ledeganckstraat 35, 9000 Gent, Belgium; 2 Center for Macroecology, Evolution, and Climate (CMEC), GLOBE Institute, University of Copenhagen, 2100 Copenhagen Ø, Denmark; 3 School of Life Sciences, University of Hawai’i at Mānoa, 2538 McCarthy Mall, Honolulu, HI 96822 USA; 4 Centro de Modelamiento Matemático (CNRS IRL2807), Universidad de Chile, Santiago, Chile; 5 CICGE—Centro de Investigação em Ciências Geo-Espaciais, Alameda do Monte da Virgem, 4430-146 Vila Nova de Gaia, Portugal; 6 Ecology, Department of Biology, University of Konstanz, Universitätsstraße 10, 78464 Konstanz, Germany

**Keywords:** Macroecology, Invasive species, Ecophysiology

## Abstract

Invasive species pose a major threat to biodiversity and inflict massive economic costs. Effective management of bio-invasions depends on reliable predictions of areas at risk of invasion, as they allow early invader detection and rapid responses. Yet, considerable uncertainty remains as to how to predict best potential invasive distribution ranges. Using a set of mainly (sub)tropical birds introduced to Europe, we show that the true extent of the geographical area at risk of invasion can accurately be determined by using ecophysiological mechanistic models that quantify species’ fundamental thermal niches. Potential invasive ranges are primarily constrained by functional traits related to body allometry and body temperature, metabolic rates, and feather insulation. Given their capacity to identify tolerable climates outside of contemporary realized species niches, mechanistic predictions are well suited for informing effective policy and management aimed at preventing the escalating impacts of invasive species.

## Abstract (web summary)

Forecasts of risks of invasion by non-native species are challenging to obtain. Here, the authors show that mechanistic models based on functional traits related to species’ capacity to generate and retain body heat identify areas at risk of invasion by non-native birds in Europe.

## Introduction

Driven by advances in technology and trade liberalization policies, globalization has had drastic effects on the economic world and societies’ way of life, facilitating unparalleled economic growth, cross-border movements of goods and a diversification of human immigrant populations^1^. Concurrently, this increased mobility led to a profound reshuffling of biodiversity, as humans transport more and more species across the globe, inadvertently or for commercial or cultural reasons. The ‘Global Report’ of the Intergovernmental Science‐Policy Platform on Biodiversity and Ecosystem Services (IPBES) alarmingly states that introduced species that become invasive and spread across their new range are one of the main direct causes of the current biodiversity collapse^2^. Invasive alien species also cause massive damage to agriculture^3^ and can pose a considerable threat to human health^4^. While these threats were recognized decades ago, and despite the implementation of legal frameworks ranging from more stringent biosafety practices to (supra)national list of invasive species of high concern^5^, invasive species accumulation across continents and taxonomic groups shows no sign of abating. The rate of species introductions is increasing and, if current trends continue, established alien species numbers globally are predicted to increase by more than 35% by 2050^6^. As removal of established invaders is costly^7^, formal risk assessments aimed at identifying likely high-impact invaders are increasingly used to set trade-restrictive regulations. For example, the European Union’s flagship initiative (Regulation 1143/2014) for meeting United Nations Aichi Biodiversity Target 9 concerning biological invasions applies evidence-based risk assessment protocols to restrict the use, trade, and transport of certain invasive alien species^8^.

Identifying the geographical areas at risk of invasion is crucial for prioritization of alien species for policy and management efforts^9^, as it relates to an invader’s overall impact^10^ and guides surveying of risk areas for early invader detection and elimination^11^. Inaccurate forecasts of areas suitable to invaders jeopardize timely risk assessment outcomes, with significant downstream effects on policy and management actions, such as the selection of suboptimal response measures and over—as well as underinvestment in mitigation efforts^12^. Predictions of the potential geographic distribution of species are primarily derived from correlative species distribution models (SDM)^13^. These tools are widely used in biodiversity assessments, for example, to select places for protected areas, habitat restoration, and species’ translocations^14^. SDMs have proved especially effective for modeling further range expansion of established invaders^15,16^, but the reliability of invasion risk forecasts obtained through SDMs fitted with only native-range distribution data is equivocal^17–20^. One possible explanation is that accurately forecasting species’ full potential distributions requires characterizing their fundamental (Grinnelian) niche, i.e., identifying all combinations of environmental variables that allow species to persist^21,22^. Quantifying the fundamental niche generally requires physiological data^23,24^. Although there is an active debate as to whether certain SDM strategies, reflecting different hypotheses on the nature of the fundamental niche, can adequately approximate it^15,25–28^, there is no broadly applicable methodological consensus for obtaining forecasts of invasive species’ full potential distributions based on native occurrence data^20,29–31^.

**Fig. 1:**
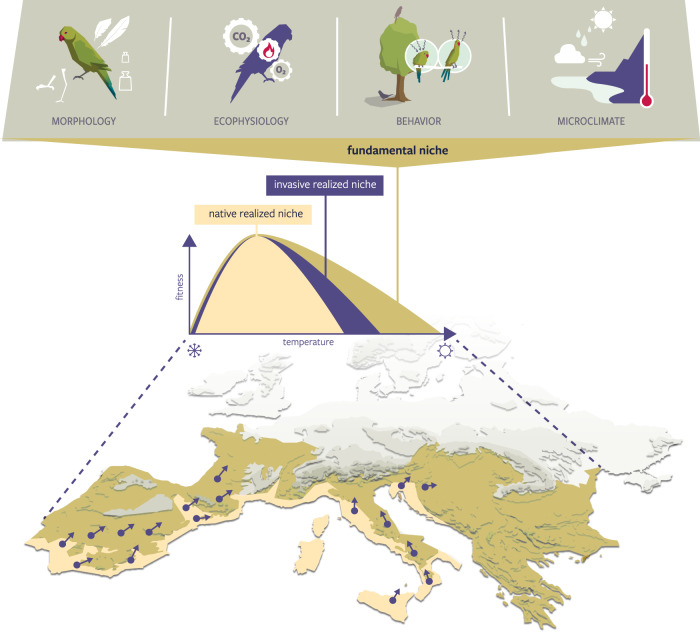
Relationships between realized and fundamental niches and the geographical areas at risk of invasion by introduced species. Native-area species distribution models are based on species’ contemporary occurrences and may underpredict the area at risk of invasion (yellow areas). Purple dot-arrows illustrate an invasive species invading and spreading beyond the areas predicted as suitable based on the climates they occupy in their native range. Mechanistic ecophysiological models, in contrast, quantify species’ fundamental thermal niches by integrating their physiology, morphology and behavior with the microclimates they experience, predicting wider areas at risk of invasion (olive-green).

Trait-centered biogeography has recently emerged as one of the main approaches for assessing how species respond when faced with changing environments^32^. For example, generalist birds with larger brains and capable of rapid population growth are more likely to succeed as invasive species and cause damage^33^. Such information can assist with the ranking of species in terms of their likely invasiveness and impacts^34^. Here, we investigate the capacity of trait-based ecology to generate spatially explicit predictions of invasion risk by focusing on a process-explicit framework (‘NicheMapper’), which relies on the ecophysiological mechanisms that underpin species’ responses to their environment^35^. Such a biophysical ‘first principles’ approach does not use species occurrences to delineate the environmental tolerances, but instead relies on functional biophysical traits that have a clear a priori interpretation^29^, allowing the role of climate in limiting potential invasive distributions to be investigated mechanistically (Fig. 1). For invasive species management, mechanistic approaches have so far been used to assess ectotherm invasion risks, showing much larger areas at risk of invasion, e.g., by African clawed frogs (*Xenopus laevis*) introduced to Europe^36^ or cane toads (*Rhinella marina*) introduced to Australia^37^. Further applications include combining estimates of modeled energy expenditure with food availability to determine where introduced carp species^38^ are most likely to invade the North American Great Lakes or to assess how climate change and human water storage practices shape invasive mosquito invasion risk across Australia^39^. Yet, because of concerns regarding the availability of sufficient information on key biophysical traits^40^, similar applications on endotherms are rare, especially for invasive species.

Here, we combine modeled microclimates with readily available species trait data obtained from museum specimens, literature review, and ecological theory to obtain mechanistic forecasts of invasion risk for a large number of sub-tropical bird species introduced to Europe. To identify those functional traits that most strongly limit invasive ranges, and to assess how predictions of geographical areas at risk of invasion vary with trait parameter estimates, we carried out a series of sensitivity analyses. We found that mechanistic models accurately predict invasive occurrences of birds introduced to Europe, especially when intraspecific variation in key limiting traits is taken into account. Model performance chiefly depended on a relatively small number of functional traits related to species’ capacity to generate and retain body heat. From a biodiversity management perspective, our findings highlight that generic mechanistic models can provide the robust and early forecasts of invasion potential that are needed for efficient resource allocation according to the risks posed by introduced species. To realize fully the promise of biophysical models for ecological forecasting across diverse species and geographic regions, the development of standardized open-access biophysical trait databases consolidating available knowledge and identifying knowledge gaps is, however, needed.

## Results

### Realized niche shifts are prevalent among invasive birds in Europe

Comparing the climates that invasive birds occupy in their native versus the invasive ranges showed that the invasion of Europe by twenty bird species of predominantly (sub)tropical origin is characterized by a marked niche expansion into colder environments, as birds had on average more than 50% of their invasive occurrences in climate conditions that are outside of their native realized niche. Niche similarity tests revealed significant niche differences for 13 of the 20 bird species, implying that differences between native and invasive realized niches are mostly not solely due to the different availability of climates in the native versus the invasive range (Supplementary Fig. 1 and Supplementary Data 1).

### Mechanistic models more accurately predict invasive occurrences

**Fig. 2:**
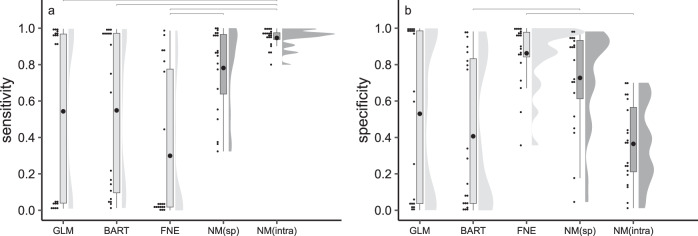
Accuracy of invasion risk predictions. Accuracy of correlative (light gray) versus mechanistic (dark gray) models in correctly predicting **a** invasive presences (‘sensitivity’) and **b** locations currently uninvaded (‘specificity’) by introduced birds across Europe (*n* = 20 introduced bird species). Each small black dot (jittered for visibility) denotes an introduced bird species. Boxplots indicate mean (large black dot) and interquartile range (i.e., 25th percentile–75th percentile), with whiskers corresponding to 1.5 times the interquartile range. Split-half violin plots visualize the probability density of the data at different accuracy values. Top gray lines indicate significant differences between correlative and mechanistic models as revealed by post-hoc Tukey tests. Correlative models: GLM (binomial Generalized Linear Models), BART (Bayesian Additive Regression Trees), and FNE (Fundamental Niche Ellipses). Mechanistic predictions were obtained through the NicheMapper platform using either species-level parameter estimates (NM_(sp)_) or allowing for intraspecific variation (NM_(intra)_).

Mechanistic forecasts of invasion risk were good to very good at identifying invasive bird occurrences across Europe, as 79% of bird presences were successfully predicted based on species-level trait estimates, and rising to 96% when accounting for intraspecific variation (Fig. 2). Both the species-level and intraspecific-level models also showed good to moderate ability to characterize areas that are likely to be climatically unsuitable, correctly identifying 73 and 36% of the locations where introduced birds are currently absent, respectively (Fig. 2, Supplementary Data 2). Mechanistic models predict that southern Europe is most at risk of invasion, while only introduced pheasants (*Syrmaticus reveesi* and *Chrysolophus pictus)* and larger parakeet species (*Myiopsitta monachus*, *Psittacula eupatria*, and *P. krameri*) are expected to tolerate the central European climate, with pheasants able to invade Scandinavia as well (Supplementary Figs. 2 and 3).

**Fig. 3:**
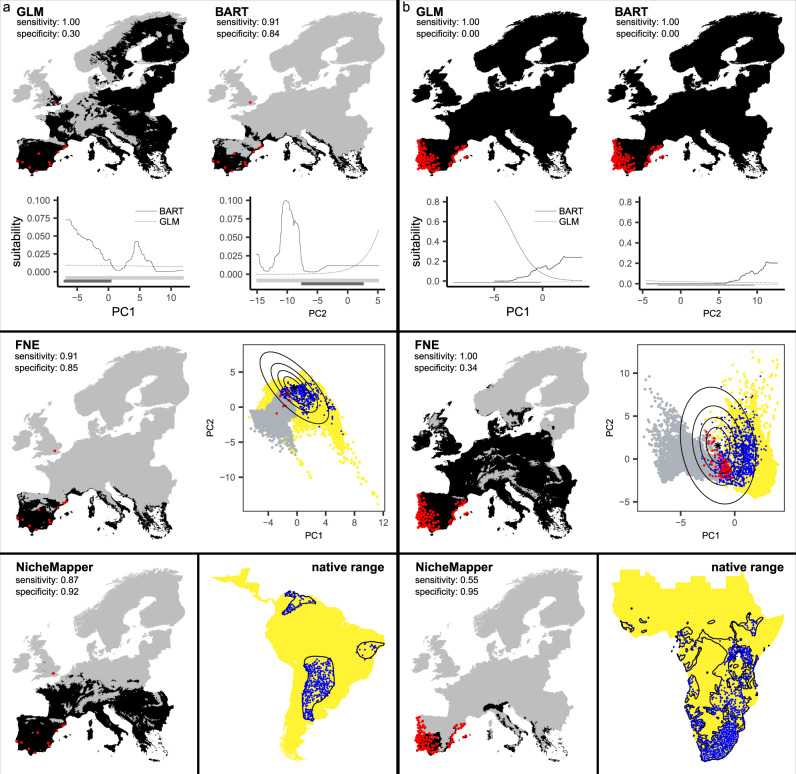
Forecasts of invasion risk. Areas at risk of invasion by introduced **a** blue-crowned parakeets *Thectocercus acuticaudatus* (body mass of ~170 g) and **b** common waxbills *Estrilda astrild* (~9 g) across Europe. Gray indicates areas predicted to be climatically unsuitable while black areas are at risk of invasion according to correlative (GLM: generalized linear model, BART: Bayesian additive regression trees, FNE: fundamental niche ellipse) versus mechanistic (species-level NicheMapper) models. Red dots represent current invasive occurrences, used to independently evaluate model forecasts, expressed as the percentage of correctly predicted occurrences (sensitivity). Thresholds discriminating suitable versus unsuitable area are based on a 5% native-range omission rate for the correlative models and on a 4.6 times the basal metabolic rate limit for the mechanistic model. Response curves in the upper panel show how climate gradients (summarized into principal component axes) drive predicted suitability, while gray and black horizontal bars show the range of each climate axis in the native and invasive area, respectively. Estimated ellipses in the middle panel (FNE) represent 25, 75, and 95% confidence regions of the modeled fundamental niche, with the star as the niche center. Yellow points represent the climate conditions accessible to the species in the native range (‘M hypothesis’), blue dots the available native-range occurrences. Gray points indicate climates across Europe, red dots the invasive occurrences. In the lower panel, the black polygons are the species’ native ranges according to BirdLife, the blue dots are occurrences obtained through GBIF, and the yellow regions are the geographical backgrounds (‘M’) used to train the correlative models.

Correlative models including biotic and habitat predictor variables better captured species’ native-range distributions (AUC ratio of 1.32 ± 0.14 when including biotic and habitat variables versus 1.28 ± 0.13 for climate-only models, all partial ROC p-values <0.05). Invasion risk forecasts, in contrast, were more accurate when using climate-only models (AUC ratio of 1.40 ± 0.30 for climate-only versus 1.31 ± 0.27 for models with biotic and habitat variables, all partial ROC *p*-values < 0.05). While the accuracy of correlative invasion risk forecasts varied with model algorithm and settings (see below), they were generally less accurate compared to the mechanistic models (Fig. 2, Supplementary Data 2–3). The fundamental niche ellipse method tended to underpredict invasive distributions, correctly identifying 79% of locations currently free from invasion but only predicting 37% of invasive occurrences. GLM correctly identified on average 58% of presences and 47% of absences, but achieved this through alternating strong over- and underprediction of species’ invasion risks, either labeling almost the whole of Europe as suitable or none of it. BART was arguably the best performing correlative method, correctly identifying 70% of occurrences and 33% of currently uninvaded locations (Fig. 2, Supplementary Data 2–3). This machine-learning method, however, also tended to overpredict potential distributions, for example designating large parts of northern Scandinavia as suitable for several small-bodied Aftrotropical estrildid finches (Estrildidae), weaverbirds (Ploceida) and lovebirds (Psittaculidae, Fig. 3, Supplementary Fig. 2).

For seven bird species, contemporary native-range distributions may not allow to capture adequately their fundamental niche, but this native niche truncation did not affect correlative model performance (Supplementary Data 3 and 4). Model accuracy correlated positively with the number of native-range occurrence data available for model training, and was higher when model forecasts were restricted to those parts of Europe that have a climate similar to the climate species encounter across their native range. When making forecasts for parts of Europe with climates different from species’ native climates, extrapolating response curves (as opposed to clamping to limit extrapolation) tended to result in more accurate forecasts. Correlative model performance was higher for species exhibiting less niche expansion in the invaded range (Supplementary Data 3, Supplementary Fig. 4). Specifying an invasive range omission rate of 2.5% was associated with higher evaluation statistics compared to a 5% rate. Restricting the invaded range to areas accessible to the species via dispersal since their introduction and the number of invasive range occurrences available for model evaluation did not affect the accuracy of model forecasts (Supplementary Data 3).

### A limited number of key biophysical traits determine invasive range distributions

**Fig. 4:**
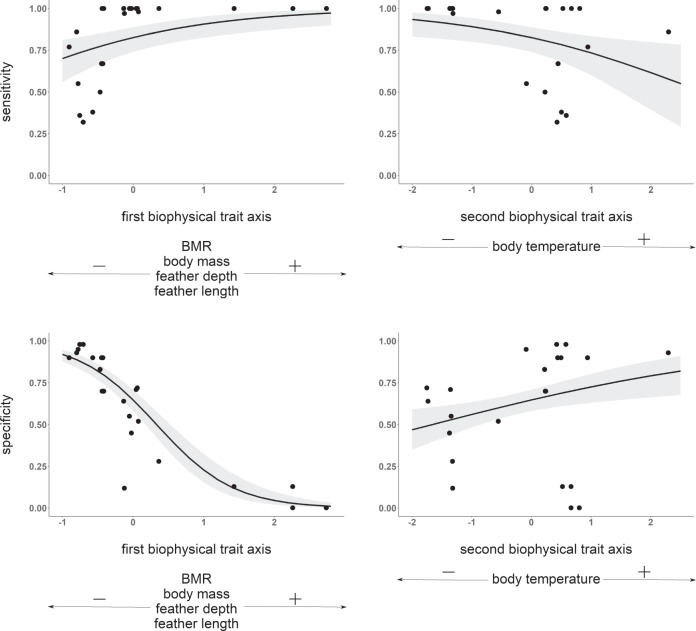
Key biophysical traits. Significant relationships between biophysical traits and mechanistic model predictive accuracy. Model performance is chiefly governed by a small number of key biophysical traits related to species’ capacity to generate and retain heat. Each dot represents a bird species, and data are slightly jittered along the x-axis for visibility. Solid black lines represent the mean estimate, gray shading indicates the 95% confidence interval of significant relationships.

Sensitivity analyses indicate that mechanistic model performance in birds is most strongly influenced by basal metabolic rate, body mass, and density, followed by feather length, feather depth, and body temperature (Supplementary Fig. 5). Model capacity to identify accurately invasive occurrences was higher for species characterized by higher body masses, higher basal metabolic rates, better plumage insulation capacity, and a lower body temperature, while the opposite was true for the capacity to predict locations where species are currently absent (Fig. 4, Supplementary Figs. 6 and 7). Please see Supplementary Notes 1 for an extended technical description of the results.

## Discussion

Globalization of trade and transport increasingly allows species to overcome their natural biogeographic barriers and spread across new ranges. To promote effective management of such invasive alien species, the ongoing IPBES Invasive Alien Species assessment has identified the development of strategies and procedures to forecast the spread of invasive alien species as a key support tool for decision makers^41^. Climate is a dominant driver of both species’ native and invasive range distributions^42,43^, but this does not necessarily imply that reliable forecasts of invasion risk can easily be obtained based solely on the native-range climate conditions a species currently occupies. Here, we show that characterizing species’ fundamental thermal niche of ecophysiological tolerance allows us to identify invasive occurrences with high accuracy (Figs. 2–3). We found little evidence of shifts in species’ fundamental thermal niches during invasion, and the successful invasion of parts of Europe by introduced (sub)tropical birds can rather be explained by species occupying different parts of their fundamental niche in different regions.

In recent decades, the pet bird trade has been dominated by the export of (sub)tropical birds to more affluent consumers in the global North^44^. Predicting the potential distribution of such introduced species is arguably one of the most difficult challenges facing biodiversity models^14^. We found that mechanistic niche models can be used to accurately guide proactive surveillance aimed at early detection. This is critically important with charismatic species such as sub-tropical birds, as culling measures to control or even eradicate large populations are costly and are not easily welcomed by the general public^45^. Species-level estimates of biophysical traits, such as upper and lower critical body temperatures or maximal heat production rates, alone often correlate poorly with distributional limits^46^. However, here we show that integrating key traits related to species’ capacity to generate and retain heat under a single process-explicit framework allows us to reliably identify geographical areas with climates that make them vulnerable to species’ invasion (Figs. 2–3). Ecophysiological insights have so far only been applied sparingly to questions of range dynamics of endothermic animals, partly because of the greater data requirements compared to correlative models. However, we have found that the cold resistance of birds is mainly determined by a limited number of input variables that characterize their mass, size, feather properties, metabolic rate and body temperature (Fig. 4). Species with special thermoregulatory adaptations, such as the use of daily torpor by hummingbirds, may require more detailed information and consideration, but our results suggest that for many species generic mechanistic models are likely to produce useful predictions of invasion risks.

Our forecasts show that most bird species introduced to Europe have ample climatically suitable areas available to spread further into (Supplementary Fig. 3). This is not unexpected, as pet bird introductions typically only stem from the 1960s and onwards^47^, and species are likely still in the early stages of invasion. Some degree of range overprediction can however not be excluded, as we did not explicitly consider the development of juvenile stages, which may prevent species establishment in areas where (nest) microclimates do not allow for nestling survival. Similarly, we may have overestimated the amount of intraspecific variation available to individuals by allowing key traits to vary according to the amount of variation found at the species-level. Finally, as we did not explicitly account for bird water requirements and water availability, our mechanistic forecasts may also have overestimated invasion risks in the warm and arid parts of Europe, as a lack of access to water may hamper the potential for evaporative cooling, with the risk of mortality from hyperthermia^48^.

The comparatively lower accuracy of our correlative model forecasts is in line with a recent meta-analysis based on >200 invasive species, stating that when niche expansion is high (as is the case here), confidence in native-range SDM predictions is low^49^. Correlative models are more readily applicable compared to mechanistic models, as they require only species occurrence data and relevant environmental factors for deriving correlations^50^. Yet, the quality and quantity of available occurrence data can have profound effects on model performance^19^. The positive association we found between the number of native-range presences available for model calibration and the accuracy of the fundamental niche ellipse approach (Supplementary Data 3) suggests that, at least for some bird species, an insufficient occurrence sample size may have prevented us from fully harnessing the predictive capacity of this method. An equally important factor to consider is the delineation of the areas across which models are calibrated, and especially so for applications aimed at estimating fundamental niches^41^. Ideally, the area across which species’ responses to environments are estimated should represent the set of sites that have been accessible to the species via dispersal over relevant periods^51^. Accurately defined calibration areas may allow us to account statistically for the fact that available occurrence data do not necessarily reflect all the environmental potentiality of a species. In our case, we lacked the detailed ecological knowledge needed to create species-specific accessible areas and account for possible abiotic barriers to dispersal such as large rivers, deep valleys or high mountain crests, whose barrier effects differ among species. Potential niche truncation^52^, which describes to what extent a limited availability of climates across the calibration area can lead to an incomplete representation of a species’ fundamental niche, was not related to predictive accuracy here. Yet, issues regarding optimal calibration area selection likely contributed to uncertainty in our correlative model forecasts.

Another factor potentially contributing to the differing performance of correlative versus mechanistic models is the difference in climate and weather variables used. Whereas our mechanistic models included a built-in microclimate module to convert a range of climate and weather data into detailed estimates of the thermal environment organisms experience, for our correlative models we followed the common practice of using WorldClim variables, representing climate conditions at a standard reference height of ~2 m above ground level^53^. Recent studies, mainly focusing on ectotherms and plants, have, however, shown that including downscaled microclimates into SDMs can significantly increase their performance, e.g., by uncovering local topography-driven climate variability, accounting for the buffering effect of vegetation cover or anthropogenic disturbances such as urban heat islands^54^. Microclimate SDM applications to endotherms are rare to date but constitute a promising avenue for further research.

Pooling occurrence data from native and invasive ranges to fit SDMs has been proposed as an alternative strategy for better approximating species’ fundamental niches, and hence, obtaining more reliable forecasts of invasion risk^15^. Here, SDMs were created using only native-range occurrence data so we could assess the usefulness of correlative models for species at very early stages of (or even before) invasion. For example, several species on the List of Invasive Alien Species of Union concern (https://ec.europa.eu/environment/nature/invasivealien/list/index_en.htm) have no recorded invasive presences yet. As the standard native-area based SDM modeling procedures applied here did not consistently result in accurate invasion risk forecasts, our results support recent calls for more research into identifying modeling procedures and conditions in which SDM invasion risk forecasts can be most reliable^18,55^. An alternative modeling strategy could be to model explicitly species’ native realized niches only, which has been done for example to identify hotspots of traded non-native birds under current and future climates^56^. This, however, comes at the cost of reduced reliability for areas where the climate is predicted to be unsuitable for the establishment and spread of invaders. In cases where the availability of high-quality native-range occurrence data is limited, and where knowledge of the species’ autecology does not allow to make informed decisions on calibration areas and relevant ecological predictor variables, our results indicate that mechanistic, ecophysiological approaches can serve as a valid alternative—even when input data are largely based on allometric scaling, literature reviews and museum specimens.

To promote the uptake of biophysical modeling in ecological forecasting, and to assess further which functional traits best predict invasive ranges under different environmental conditions, it is imperative to increase the availability of reliable biophysical functional trait information. While over the past decades, a wealth of ecophysiological data has been gathered, the development of standardized open-access databases for functional traits has lagged^57^. We here find that at least for macro-ecological predictions of invasion risk of sub-tropical birds introduced to Europe, a relatively small number of key functional traits suffices—yet parameter values for these traits still typically need to be retrieved through bespoke literature reviews, combined with empirical measurements on live or museum specimens. For example, repositories such as the recent AVONET database^58^ are described as providing comprehensive functional trait data for all extant bird species worldwide, however, from a biophysical point of view, this database contain mainly descriptive phenotypic traits such as wing, tail and tarsus lengths. While the widespread availability of such traits allows for testing of evolutionary and macro-ecological hypotheses (e.g., on drivers of rapid morphological diversification^59^, or on the role of divergent adaptation during speciation^60^), the sole biophysical functional trait (i.e., an individual property that has a connection to organismal performance in the context of energy and mass exchanges between an individual and its environments^61^) that is readily available for most bird species is body mass. Biophysical functional traits such as plumage depth and feather length, which influence the capacity for thermal insulation, are not more difficult to measure than traditional phenotypic traits but their importance is hitherto largely unknown to the wider scientific community, hindering data collection and sharing.

To support the development of robust and interpretable biophysical models of species’ distributions, building databases, including the decision on which functional traits to prioritize, should be based on, and in dialog with, ecological theory. Collecting empirical measurements for large numbers of species will remain a challenge for the foreseeable future, especially for ecophysiological functional traits, such as metabolic rates, body temperatures or evaporative water loss, that can only be collected using often time-consuming and costly experiments. Therefore, instead of simply collecting more biophysical trait data for more species, studies should focus on collecting those data that best enable the testing, development and refinement of predictive hypotheses about how biophysical functional traits are likely to vary across space, time and phylogeny. For example, empirical measurements of (basal) metabolic rates—which determine heat generating capacity—are currently available for only about 10% of the world’s bird species^62^. Although body-mass-based scaling laws^63^ may serve as a first reasonable approximation, much uncertainty remains about the factors underlying inter- and intraspecific variation in metabolic rates (e.g., whether tropical birds are characterized by lower and less plastic metabolic rates compared to birds from temperate regions^64^). Similar unresolved questions exist regarding body temperature regulation^65^ (e.g., whether the plasticity of upper limits is much smaller than that of lower limit plasticity^66^) and how birds trade off water conservation against avoiding lethal hyperthermia via evaporative cooling^67^. A better mechanistic understanding of these traits is key to predicting invasion risks, for example in hot and arid areas, where ranges are more likely to be limited by the species’ heat tolerance^48^.

For (sub)tropical birds introduced to Europe, our results show that several introduced bird species, most notably parakeets and waxbills, have established populations in parts of Europe with climates that are close to the maximal thermoregulatory costs they can bear (Supplementary Fig. 3). They may be able to do so because they are effectively subsidized by the abundant availability of energy rich food at bird tables^68^ or in highly productive rice fields^69^. To assess the extent and rate at which invasive birds will be able to fill their potential climatic distribution, ecophysiological estimates of discretionary energy (i.e., the amount of energy available to individuals for growth and reproduction after accounting for thermoregulatory needs^70^) can be combined with information on habitat availability and species’ colonization potential. As a first practical approximation, this can be achieved by using the presence or absence of required habitats as a filter to refine invasion risk maps^56,71^. Trends in data availability and computational power also increasingly allow to employ process-explicit population-level models that constrain demographic rates or carrying capacity to suitability surfaces^72^. Given their capacity to identify climatically suitable areas that are outside of species’ contemporary native realized niches, ecophysiological energetic surfaces are ideally suited for models aimed at identifying areas that can sustain viable invasive populations. Combined with spatially explicit dispersal models (which can range from simple cellular automata to more complex tools simulating dispersal heterogeneity among individuals^73^) and estimates of biotic resistance to invasion (e.g., based on species compositions of both source and incipient communities^74^), forecasts of temporal patterns of invasive spread and potential invasion corridors can then be obtained^75^.

Our analyses thus strengthen the case for including biophysical modeling of the mechanisms underlying the physiological response of organisms to their environment as a key component in the ecologists’ toolbox, to forecast where non-native species may invade. Recent developments such as the availability of free and open software packages to conduct climate downscaling (e.g., ‘microclima’^76^) and biophysical modeling (e.g., ‘NicheMapR’^77^) that allow to link up to high performance computing facilities, combined with dedicated tutorials and visualization tools (e.g., through the TrEnCh and CAMEL projects), create an ideal moment for biophysical ecology to rise to the daunting challenge of improving forecasts of biodiversity under global change^78^.

## Methods

### Species occurrence data

From the set of known non-native bird species in Europe^79^, we selected terrestrial bird species that occurred in at least five different locations (the minimum sample size for niche dynamic analyses^80^), that did not have any part of their native range in Europe, and for which museum specimens were present in the Royal Belgian Institute of Natural Sciences (RBINS, Brussels, Belgium), resulting in 20 species for this study. Species occurrence data for native and invasive (i.e., European) ranges were obtained through the Global Biodiversity Information Facility portal (GBIF; see Supplementary Table 1). For the invaded range, to minimize the risk of including species observations that do not reflect established, self-sustaining populations, occurrences were only retained when literature sources confirmed the presence of established breeding populations^69,79,81–88^. All occurrence data were rarefied using a distance of 50 km^52^ to minimize spatial autocorrelation (except for *Agapornis personatus*, *A. fischeri*, *A. roseicollis*, *Acridotheres tristes*, and *A. cristatellus*, where a distance of 10 km was used for the invasive range to retain a minimum of 5 occurrences). To delimit the native range of each species, we first considered occurrences that were within the species’ natural distribution range, as given by BirdLife’s extent of occurrence digital maps^89^. Then, we buffered the original range maps with a distance of 0.5° to reduce potential errors associated with georeferencing and digitalization procedures, and we excluded those areas occupied only during the non‐breeding season or during migration^90,91^. Next, the remaining GBIF points were fed to the R CoordinateCleaner^92^ package, using its default settings tailored for cleaning occurrence records from biological collection databases. This package removes occurrences with potentially problematic geographical coordinates, such as country centroids, equal longitude-latitude observations, GBIF headquarters, biodiversity institutions and zero coordinates.

### Niche dynamics during invasion

To assess niche dynamics during the invasion process and to obtain correlative forecasts of invasion risk, native and invasive range occurrence data were used together with the full set of 19 bioclimatic variables available at the WorldClim v2 repository^53^. Then, for each species, we delimited native and invasive ‘background’ areas (or, M hypothesis) that reflected the set of sites accessible to a species through dispersal over its recent history^51,93^. For the native range, these background areas were obtained by applying a spatial buffer surrounding available rarefied occurrences, with a radius equaling the mean pairwise great-circle distance^94^ (calculated through the ‘geobuffer’ R package) between occurrences, and cut with the zoogeographical regions^95^ where the species is native to account for major biogeographical barriers to dispersal^96^. We refer to these areas as calibration areas or native backgrounds, since they were used to calibrate the species distribution models. For the invasive range, we explored two alternative background areas. First, we considered the whole of Europe as invasive background area (‘EU-background’), reflecting the assumption that pet bird trade has given bird species the opportunity to escape and establish populations across the continent^44^. Second, we created a more restricted invasive background by buffering known introduction locations with a distance equal to residence time (i.e., introduction date) multiplied by a general estimate of bird invasion speeds (see refs. ^90,97^ for details), combined with the location of known failed introduction events, buffered with 50 km (‘invasion history background’).

We then used a principal components analysis (PCA) to transform the environmental space of the 19 environmental variables into a two-dimensional surface defined by the first and second principal components. The PCA was calibrated using environmental data at all sites comprising the native and invaded areas (thus, once using the EU and once with the invasion history background). The PCA scores of the occurrences of each species were then projected onto a 100 × 100 grid of cells bounded by the minimum and maximum PCA values in the background data. A smoothed density of occurrence for each species in each cell of the PCA grid was estimated using a kernel density function^80,98^ and used to calculate metrics of niche dynamics.

Niche dynamics were studied by testing for niche equivalency and similarity, and by calculating metrics of niche expansion, stability and unfilling^80,98^. Niche expansion was calculated as the proportion of species occurrence densities in the non‐native distribution located in climates other than the native distribution. Niche stability is the proportion of occurrence densities that overlaps with the native distribution climate conditions, and niche unfilling is the proportion of occurrence densities in the native distribution located in climates other than the non‐native distribution^98^. Note that these metrics are calculated only on the environmental space that is shared between both ranges, and using the intersection of the 75^th^ percentile of climates in each range^80,98^. Tests of niche similarity and equivalency followed randomization tests as outlined previously^80^. Rejection of niche equivalency means that the niches of native and non‐native populations are not statistically equivalent, while a rejection of niche similarity indicates that niches are more similar than expected at random.

### Correlative species distribution modeling

We applied three different algorithms for species distribution modeling. Our algorithms include (1) a novel presence-only ‘envelope’ type model assuming an ellipsoidal shape of the fundamental niche in climate space^99^, (2) a state-of-the-art machine-learning method called Bayesian Additive Regression Trees (BART, computed with the ‘embarcadero’ R package^100^), and (3) a classic statistical method, namely logistic Generalized Linear Model^101^ (GLM, computed with the ‘glm’ function of base R). The ellipsoidal envelope method assumes that the fundamental niche of the species has a convex, symmetrical shape in climate space and accounts for the fact that the observed occurrences are constrained by the dispersal abilities of the species which define the subregion of the fundamental niche that is currently occupied by the species (i.e., the realized niche), and hence, the subregion that is observable. This approach has shown to be useful in predicting the occurrence sites of an invasive species outside of its native range^99^. The GLM approach has shown to be a simple yet robust and generalizable method^102,103^, while BART provides highly accurate predictions without overfitting to noise or to particular cases in the data^104,105^.

All three modeling algorithms were first run using the above-mentioned first and second principal components as bioclimatic predictor variables. To account for possible geographical sampling biases due to differences in human accessibility, we applied the ‘*sampbias*’ R package^106^. This method uses a Bayesian approach to estimate how sampling rates vary as a function of proximity to bias factors such as the presence of roads, rivers, airports and cities. Spatially explicit estimates of sampling rates were then included as covariates when training SDMs on the native range, and were set to zero for invasive range predictions^107^. As it currently is not yet possible to include such bias correction into the fundamental niche ellipse method, accessibility biases are only implemented for the GLM and BART analyses. To verify the assumption that species’ range boundaries are primarily determined by climate^43,108^, GLM and BART models were also run with climate, habitat and biotic interaction variables (note that the presence-only fundamental niche envelope model^99^ is geared towards climate niches and can consequently only handle two predictor variables). As habitat variables, we selected a set of 14 habitat heterogeneity measures based on the textural features of enhanced vegetation index (EVI) imaginary^109^. As biotic interaction variables, representing main food and nest resources^110^, we selected eight variables available from the Copernicus Global Land Service (cover of bare ground, crops, forest, grassland, moss and shrub cover, as well as the presence of permanent and seasonal water bodies; averaged over the available 2015-2019 time series)^111^. We then used a PCA analysis to retain two ‘habitat’ and two ‘biotic’ principal components in the models.

GLM and BART species distribution models were computed using native-range occurrence data only, and a five-fold spatial block cross-validation resampling method^112^ to generate the model training and test datasets in the species’ native backgrounds. Cross‐validation first splits the data into K (here: five) roughly equal‐sized parts, and then fits the models *K* times. Each time one part is used as test data and the other *K*–1 parts of the data are used as training data. While conventional random cross-validation can underestimate prediction error, spatial block cross-validation allows a more rigorous assessment of model performance, as it reserves both nearby and more distant places for model testing. Spatial blocks were computed with the ‘blockCV’ R package^112^ using a 200-km range, which was considered a reasonable distance across species. After calibrating the models with the three different algorithms for each species, we proceeded to calculate different evaluation metrics. To obtain forecasts of invasion risk across Europe, we (1) allowed models to extrapolate beyond their training data range and (2) adopted a ‘clamping’ approach, i.e., holding predicted suitability at constant beyond the limits of training environments^113^ using the ‘clamp.vars’ function of the R package ‘ENMeval’^114^.

To generate binary predictions of invasion risk across Europe, we applied both a 2.5 and 5% training omission threshold^25^, based on the native-range occurrence data used to calibrate the models. We assessed the accuracy of these invasion risk forecasts by computing the sensitivity (i.e., the proportion of correctly predicted invasive presences) and specificity (i.e., correctly identified locations without invasive presences) of the models when extrapolated to the invaded region, using the ‘threshMeasures’ function of the ‘modEvA’ R package^115^. Using the same threshold values, we also calculated the partial area under the receiver operating characteristic (ROC) curve and its statistical significance, using the ‘kuenm_proc’ function of the ‘kuenm’ R package^116^ with 1000 iterations for each species. Evaluation statistics were calculated based on the two alternative invasive range background areas defined above (i.e., EU versus invasion history background). To assess to what extent extrapolation to climate conditions non-analogous to those in the native-range calibration areas affected model accuracy, we first identified regions of strict extrapolation using the Mobility-Oriented Parity method (MOP)^117^ using the ‘mop’ function of the ‘enmSdm’ R package^118^. MOP compares the environmental breadth of predictor variables in the native-range calibration areas with that in the projection area (i.e., Europe). We tested for the influence of extrapolation by recalculating evaluation metrics based on maps excluding areas where strict extrapolation was present. Lastly, we calculated to Potential Niche Truncation Index, a metric that describes how much of the perimeter of the climate space occupied by the species abuts, overlaps, or is outside the margins of the environment’s (available) climate space^52^. The larger the proportion of the occupied niche that is truncated in the available climate space, the higher the risk that the occupied niche may poorly reflect the fundamental niche. All R scripts used for species distribution models are available in a public GitHub repository at https://github.com/LauraJim/Modeling_bird_invasions. Model procedures have been detailed through the ODMAP (Overview, Data, Model, Assessment and Prediction) protocol^119^ presented in Supplementary Table 2.

### The NicheMapper ecophysiological modeling platform

Ecophysiological, mechanistic forecasts of invasion risk were obtained using NicheMapper, a mechanistic bioenergetics model that uses an animal’s morphological, physiological, and behavioral traits in combination with the ambient microenvironment in order to identify geographical areas with climates that are within a species’ fundamental thermal niche. NicheMapper consists of two submodules: (a) a microclimate model that generates, for each landscape pixel, hourly calculations of solar and infrared radiation intensities, above‐ground profiles of air temperature, wind velocity and relative humidity, and (b) an endotherm model that calculates metabolic rates based on a heat balance equation in which the animal’s metabolic heat production must equal the net heat loss to its microenvironment to maintain a stable core temperature. The simulated metabolic rate is then compared to a target metabolic rate for any given hour (using the basal metabolic rate when the animal is resting, or basal rates multiplied by an activity multiplier when the animal is active) to determine whether thermoregulatory actions to avoid hypo- or hyperthermia should be taken. If the simulated metabolic rate is beyond ±5% (arbitrary error accepted by the model) of the target range, the model allows animals to employ both physiological and behavioral thermoregulation (e.g., varying flesh thermal conductivity to simulate vasodilation or vasoconstriction, seeking shade)^70,120^ until a heat balance is achieved. The model thus calculates the necessary (in cold temperatures) or allowable (in warm temperatures) metabolic rate that will enable an animal to maintain its body temperature within a tolerable range. Metabolic rates hereby represent hard limits for survival: if the simulated metabolic rate falls below the basal metabolic rate, the animal is assumed to have died of overheating, if the simulated required energy expenditure exceeds the estimated maximal energy intake, the animal is assumed to have died of hypothermia.

### Ecophysiological data inputs

*The microclimate model* uses approximately sinusoidal equations to translate long-term, gridded monthly climate averages, which are typically derived from weather stations placed approximately 1–2 m above the ground into estimates of hourly microclimates at the animal’s average height, representing mid-month conditions for a calendar year (i.e., 12 total model days)^77^. It relies on information about monthly maximum and minimum daily average temperature, relative humidity, cloud coverage, wind speed, snow presence/absence, topography, solar radiation and soil properties. Minimum and maximum temperature data and windspeeds were obtained from the WorldClim v2 repository^53^. We used the same average wind speed throughout the day because no maximum/minimum values were available. Windspeeds were modified based on habitat cover and set to 50% of the reference value for forests^120^, 90% for shrubs and/or herbaceous vegetation^120^ and 75% for urban areas^121^, whereby land-cover was derived from the CORINE Land Cover inventory^122^. Cloud cover was taken from CRU v2.0^123^, and we assumed the same cloud cover for every hour within a given month. Presence or absence of snow cover for each month of the year was taken from NOAA Northern Hemisphere SCE v1.0^124^. Hourly relative humidity profiles were calculated assuming a daily maximum of 100% and minimum values were calculated based on the daily temperature range assuming constant water mass in the air over 24 h^125^. All climate and weather data used here span the period 1970–2000, except for cloud cover, which represents averages for the period 1960–1990. Data were downloaded at the highest resolution available and rescaled to 10 x 10 km grid pixels. Topography (elevation, slope and aspect) was derived from the Copernicus EU-DEM dataset. Clear sky solar radiation was calculated based on date, hour and geographic location. Using a built-in subroutine, the timing and intensity of radiation reaching the ground were further adjusted according to horizon angle, slope, aspect and cloud cover^126^. Based on the Soil Atlas of Europe, each landscape pixel was assigned to a generalized soil type (i.e., either sand, rock or soil)^127^, using the corresponding values for thermal conductivity, density, specific heat and substrate reflectivity; with the exception of urban areas (as identified by CORINE Land Cover, see above), which were assigned the properties of (Portland cement) concrete, as derived from www.engineeringtoolbox.com. Pixels that had more than one soil types at a 10x10km resolution were modeled using a weighted average of the parameter values of the constituent soil types. Average animal height was set at 150 cm above ground level for Psittaciformes, as these are mainly arboreal species but descending to lower heights, e.g., for foraging. For Estrildidae, Viduidae and Ploceidae, we used 120 cm reflecting their habitat preference for scrubs and reedbeds. As Sturnidae and Phasianidae mainly forage on ground-level, for these species we used approximate breast heights of 10 and 20 cm, respectively.

*The endotherm model* requires information on a set of biophysical and behavioral species traits. Morphological traits: Bird body dimensions were measured from museum specimens, body-mass estimates were taken from the literature and average bird density was estimated at 875 kg/m³^128,129^. Body fat was considered to be present subcutaneously on bird neck and torso, and taxon-specific fat percentages were derived from^130,131^. Within the model, adjustments to morphometric measurements of each body part were made when model-calculated densities of body parts (flesh only, without feathers) differed by more than 5% from the 875 kg/m³ estimate above^129^. These adjustments were allowed because body masses were taken from the literature while morphometric parameters were measured from museum specimens. Feather length, depth and solar reflectivity were measured dorsally and ventrally for each body part. Using the same body locations as for feather length and depth, solar reflectivity was measured across 300–2100 nm using a dual spectrophotometer^132^. Solar reflectivity of legs and beak was set at 0.33^129^. For species that have (seasonally) sexually dimorphic plumages, reflectivity was calculated separately for males and females, assuming that outside of the breeding season, males had the same reflectivity as females. Ecophysiological traits: body temperature^133–139^ and basal metabolic rates (BMR) were taken from literature on the focal species, from closely related species, or based on allometric scaling relationships^63^. BMR multipliers for daily foraging activity were set at 2.5^140–142^, and an additional energetic multiplier of 1.5 was implemented to account for heat generated during breeding. Muscle efficiency was assumed to be 25%^143^, flesh-specific heat capacity was modeled at 4185 J/Kg.C^144^ and flesh thermal conductivity was allowed to vary between 0.41 and 2.80 W/m.C^145^ (simulating vasodilation or vasoconstriction). We estimated that 1% of the skin functioned as a free water surface to account for the eyes and thin skin, and assumed that effective percent wet skin can rise up to 5% under heat stress^146^. Birds were also allowed to increase plumage depth to simulate ptiloerection under cold stress. Behavioral traits: to preserve homeothermy, birds were assumed to first employ ecophysiological responses before activating behavioral modifications. All birds were considered to be active during diurnal and crepuscular periods^147^. More details on the exact parametrization of NicheMapper for our bird species can be found in Supplementary Methods 1.

### Ecophysiological model building and evaluation

Ecophysiological models usually assume that a single individual is representative of the species as a whole. Here, we first ran such a species-level model, which was then subjected to a sensitivity analysis uncovering the most influential variables (see Latin Hypercube sampling below). These variables were then considered in an intraspecific-level model accounting for trait variation.

To obtain such a *species-level model*, whenever multiple measurements were available for a species, the mean was taken to obtain a single value. While NicheMapper allows some traits (body mass and fat, feather lengths and depths) to vary throughout the year, given a lack of information, all were considered to be constant throughout the year. In order to persist in a given location, we assumed birds need to be able to survive throughout the year and to complete a breeding cycle. As little or no information is available on the breeding phenology of most introduced birds across Europe, we separately modeled energetic requirements for yearly survival (i.e., ‘daily foraging’ BMR multiplier of 2.5 for each month of the year, see above) and breeding (i.e., an additional energetic multiplier of 1.5, see above). For any location (i.e., pixel), to be suitable in any given month, modeled energetic requirements (a) cannot correspond to a metabolic rate lower than the BMR and (b) cannot exceed digestible energy intake, which for birds is generally considered to be ~4.6 times the BMR^148,149^. For annual survival, a location was deemed suitable only if energetic demands could be met for each month of the year. For breeding, we first obtained the average duration (months) from egg laying to fledging from the literature^69,82,150–163^, rounded up to the nearest integer) and areas were only considered suitable when energetic demands could be met for sufficient consecutive months. A pixel was considered at risk of invasion only when the climate allows for survival year-round and for the completion of at least one breeding cycle. For those birds exhibiting (seasonal) sexual dimorphism, survival and breeding were separately modeled for males and females, and only locations where both sexes can meet energetic demands were considered as suitable. Note that here we focus on survival of and breeding by adult birds only, and did not account for the survival and development of eggs and nestlings. The resulting binarized maps, representing areas that are in- versus outside a species metabolic’ reach (i.e., at risk of invasion or not), were used to calculate model sensitivity and specificity, using the same invasive range occurrence data as for evaluating the correlative models.

Next, we used an (iterative) Latin Hypercube Sensitivity (LHS) analysis to assess the impact on model performance of varying the values of a set of key functional traits (i.e., BMR, BMR multipliers, body mass, body density, body fat and body core temperature, the estimated temperature differential between body core and skin, feather diameter, feather length, feather depth, and feather solar reflectivity, temperature difference between inhaled and exhaled air)^70,149,164–166^. Parameter values were allowed to vary within the range of values contained within our empirical measurements or present within the literature surveyed (Supplementary Data 5). Feather lengths and depths were scaled according to the total mass of the modeled bird assuming isometric scaling^167^. Latin Hypercube sampling simultaneously varies the values of the input parameters to efficiently sample the parameter space^168,169^, and was used to first generate 1.000 model variants for each bird species considered here. Variable importance was then obtained by fitting a random forest^170^ to model outcomes (using the True Skill Statistic TSS, an evaluation metric providing an equal weighting to commission and omission errors)^171^. We then selected all models whose settings resulted in TSS values larger than 0.3^172–174^. For all variables that had a variable importance larger than 5%, we assessed the range of parameter values that characterized these well-performing models. The resulting, trimmed parameter space was then used as input for a second LHS iteration. This process was repeated until the reduction in the range of parameters values fell below 5%. For birds showing (seasonal) sexual dimorphism, LHS analyses were run for males and females separately. To assess how changing the values of the most important input parameters turns into changes in the area identified as suitable by the biophysical models, we used the percentage of Europe predicted to be at risk of invasion as dependent variable and the LHS generated values of the input parameters as dependent variables in a mixed beta-regression framework, specifying species and LHS iteration as random effects. Input data for mechanistic models can be found at https://github.com/LauraJim/Modeling_bird_invasions (NicheMapper alomvars.dat and endo.dat files).

Lastly, to obtain ecophysiological predictions accounting for intraspecific variation (‘*intraspecific-level model*’) in key biophysical traits, instead of running NicheMapper using a single value for each trait, for each pixel, 100 LHS model variants were run, assigning to the pixel the lowest amount of energy needed for thermoregulation present among the model variants considered. Key biophysical traits considered were BMR, body mass, body density and body temperature, feather depth and length (cfr. results of the species-level LHS analyses, see results). For computational reasons, and given the smaller parameter space (six traits versus 14), we here only ran 100 model variants^175^. The predictive accuracy of models at the intraspecific level was assessed on the basis of sensitivity and specificity, as above for the model at the species level.

### Statistical analyses

To investigate differences in model evaluation criteria between modeling approaches, we used linear mixed models, specifying species identity as a random effect. When AUCratio was the dependent variable, we used a Gaussian error distribution, but for specificity and sensitivity, we adopted a beta-regression approach suitable for analyzing dependent variables ranging between 0 and 1 (R package ‘glmmTMB’^176^). Model contrasts were set up using the ‘emmeans’ function of the R library ‘emmeans’, resulting in Tukey p-values adjusted for multiple testing. To explore which factors influence model forecasts, we ran similar linear models using the different model evaluation statistics as dependent variables, and the explanatory variables: native-range occurrence sample size, invasive range occurrence sample size, the potential niche truncation index and the niche dynamic indices (i.e., niche expansion). Lastly, to assess whether key biophysical traits relate to correlative and ecophysiological model performance, beta-regression models were created using the ‘betareg’ package^177^. As several biophysical variables were strongly correlated, we first used a PCA to retain two independent ‘biophysical trait’ axes (explaining 82.2% of the variation; Axis 1: 65.1%, Axis 2: 17.1%). Given the pervasive influence of propagule pressure on invasive species distributions^178,179^, we also included residence time in the invasive range (i.e., time since first successful introduction) and the number of successful introduction events as covariates in these analyses. Propagule pressure data were taken from^47,79^.

### Reporting summary

Further information on research design is available in the Nature Portfolio Reporting Summary linked to this article.

## Supplementary information

Supplementary Information

Peer Review File

Supplementary Data 1

Supplementary Data 2

Supplementary Data 3

Supplementary Data 4

Supplementary Data 5

Reporting Summary

## Data Availability

The data gathered and processed, and resulting model outcomes (GIS maps and associated model evaluation statistics), are openly available via the Zenodo database under accession code 10.5281/zenodo.7733648.

## Code availability

Code used for parametrizing the mechanistic and correlative models are available and openly accessible at https://github.com/LauraJim/Modeling_bird_invasions. Mechanistic models were run using the full NicheMapper endotherm module, available as proprietary software that needs to be purchased from http://niche-mapper.com/.
