# Supplementary material for: Mechanistic models project bird invasions with accuracy
Source: Nat Commun. 2023 May 2;14:2520. doi: 10.1038/s41467-023-38329-4 (PMC10154326; doi:10.1038/s41467-023-38329-4)

Supplementary Material

Supplementary Notes 1 – Extended technical description of results

Realized niche shifts are prevalent among invasive birds in Europe.

Niche dynamics during invasion were assessed based on bird occurrences obtained through the Global Biodiversity Information Facility (GBIF) web portal (Supplementary Table 1). To ensure data are indicative of suitable climates, invasive range occurrences were only retained when reasonable evidence of self-sustaining breeding populations was available while native range occurrences were confined to areas within species' native extent-of-occurrence maps. To minimize sampling biases, occurrences were rarefied at a 50 km distance. Climatic niches were characterized using the full set of 19 bioclimatic temperature and precipitation variables available in the WorldClim v2 repository. We found that the invasion of Europe by twenty bird species of predominantly (sub)tropical origin is characterized by a marked niche expansion into colder environments, as birds had on average more than 50% of their invasive occurrences in climate conditions that are outside of their native realized niche (niche expansion metric: mean 0.52±0.47, median 0.61, when considering the whole of Europe as accessible background area; 0.53±0.49, 0.75, when considering a more limited background area based on invasion history and dispersal). Niche similarity tests uncovered significant niche differences for 13 (European) to 14 (dispersal-constrained background) out of the 20 bird species, meaning that differences between native and invasive niches are mostly not due to a differing availability of climates in the native versus the invasive ranges (Supplementary Figure 1, Supplementary Data 1).

Mechanistic model accuracy in predicting invasion risks.

Mechanistic forecasts of invasion risk were obtained using the NicheMapper framework, an animal energetics model that does not rely on any information about a species'

distribution for parametrization but that instead leverages physiological, morphological and behavioral animal properties to identify geographical areas within a species' fundamental (thermal) niche. Mechanistic models were good to very good at correctly identifying invasive bird occurrences across Europe (sensitivity estimates 0.79±0.24, and up to 0.96±0.06 when accounting for intraspecific variation). When estimated against a European background, models retain a good to moderate capacity to characterize locations where invasive species are (at least currently) absent (model specificity 0.73±0.27 and 0.36±0.22 respectively), decreasing to 0.37±0.29 and 0.19±0.17 respectively when using the dispersal-constrained background (Supplementary Data 2). Mechanistic models predict that southern Europe is most at risk of invasion, as only introduced pheasants (*Syrmaticus reveesi* and *Chrysolophus pictus)* and larger parakeet species (*Myiopsitta monachus*, *Psittacula eupatria* and *P. krameri*) are expected to be able to tolerate central European climates, with the pheasants likely to invade in Scandinavia as well (Supplementary Figure 2-3).

Correlative model accuracy in predicting species' distributions across their native ranges.

Across the native range, model performance varied with modeling algorithm, predictor variables used and evaluation threshold considered. All models performed better than random expectations (i.e. all pROC p-values < 0.05), and model accuracy was higher when using a 5% omission threshold (AUCratio of 1.32±0.13) versus a 2.5% threshold (AUCratio 1.28±0.14, p-value = 0.0006). The effect of predictor variables differed between models, whereby BART and GLM models including habitat and biotic predictor variables outperformed climate-only models (BART: AUCratio 1.46±0.11 vs 1.36±0.12, p-value < 0.0001; GLM: 1.31±0.10 vs 1.23±0.11, p-value < 0.0001) but no such differences were found for the fundamental niche ellipses (FNE: AUCratio 1.21±0.069 vs 1.23±0.12). The higher accuracy of BART and GLM models that incorporate habitat and climate variables is driven by a higher capacity to

discriminate locations where species are absent (BART: specificity of 0.57±0.088 when including habitat and biotic interaction variables vs 0.47±0.10 for climate only, p-value < 0.0001; GLM: 0.38±0.12 vs 0.29±0.13, p-value < 0.0001). Model capacity to correctly predict bird presences across the native range was independent of predictor variables used (BART: sensitivity of 0.89±0.073 when including habitat and biotic interaction variables vs 0.91±0.064 for climate only, GLM: 0.94±0.034 vs 0.95±0.031). Please see Supplementary Data 3 (tab 'SDM native range evaluation') for detailed reporting of statistical results.

Correlative model accuracy in predicting invasion risks.

Correlative forecasts of invasion risks based on climate-only models had higher AUCratios compared to models incorporating habitat and biotic interactions predictors as well (BART: AUCratio of 1.28±0.24 for climate-only versus 1.23±0.23 including habitat and biotic interaction variables, p-value = 0.0025; GLM: 1.39±0.30 vs 1.30±0.29, p-value < 0.0001; FNE: 1.62±0.25 vs 1.37±0.27, p-value < 0.0001). The accuracy of climate-only correlative models varied with model algorithm and settings. For BART and GLMs, AUCratios were higher when restricting model forecasts to those parts of Europe that have a climate similar to species' native ranges as identified by MOP-analyses (BART: AUCratio of 1.26±0.24 when restricting to climatically similar areas vs 1.19±0.22 for the whole of Europe, p-value = 0.024; GLM: AUCratio of 1.42±0.30 vs 1.36±0.30, p-value = 0.0056; FNE: 1.62±0.25 vs 1.63±0.25). Allowing responses curves to extrapolate as opposed to clamping led to higher AUC values for GLM and FNE (GLM: AUCratio of 1.43±0.30 when extrapolating vs 1.34±0.30 when clamping, p-value = 0.010, FNE: 1.69±0.20 vs 1.55±0.27, p-value < 0.0001), but not for BART models (1.23±0.23 vs 1.23±0.2). Restricting the geographical background for model evaluation to geographical areas likely accessible to species via dispersal since their introduction dates did not affect AUCratios, nor did varying the invasive range omission rate (from E= 2.5 to 5%).

In terms of model sensitivity, correlative model forecasts were influenced by invasive range omission rate and model algorithm. Specifying a 2.5% omission rate was associated with a higher capacity to identify invasive occurrences for BART and FNE (BART: 2.5% model sensitivity of 0.70±0.37 vs 0.57±0.44 when using 5%, p-value=0.0013; FNE: 0.37±0.45 vs 0.28±0.39, p-value=0.0048) but not when using GLM (0.58±0.47 vs 0.54±0.49). Model sensitivity was highest for BART models, followed by GLM and FNE (all p-values < 0.011). Model capacity to identify locations currently free from invasive birds depended on invasive range omission rate and model algorithm. Specifying a 2.5% omission rate was associated with a lower model specificity for BART models (2.5% model specificity of 0.33±0.38 vs 0.40±0.41 when using 5%, p-value=0.0082) but not for GLM and FNE (0.47±0.46 vs 0.50±0.47; FNE: 0.79±0.28 vs 0.83±0.26). Model specificity was highest for FNE models, followed by GLM and BART (all p-values < 0.0001). Please see Supplementary Data 3 (tab 'SDM invasive range evaluation') for detailed reporting of statistical results.

Factors influencing the performance of correlative invasion risk forecasts

Sample sizes for model training (i.e. the number of available native range occurrences) only influenced the FNE models, and native range sample size positively correlated with model sensitivity (p-value=0.002) and specificity (p-value=0.046). Invasive range sample size was independent of model accuracy (all p-values > 0.17, Supplementary Data 3 (tab 'SDM invasive sample size' and tab 'SDM invasive sample size')). Niche expansion strongly influenced correlative model performance, being negatively correlated with model sensitivity (FNE p-value < 0.0001, GLM p-value = 0.014, BART p-value = 0.082) and positively with specificity (FNE p-value < 0.0001, GLM p-value = 0.016, BART p-value = 0.18). Mechanistic model performance was unrelated to the degree of niche expansion (all P > 0.421, Supplementary Data

3 tab ‘niche dynamics’, Supplementary Figure 4). While three out of 20 bird species show moderate and four strong risk of native-range niche truncation (Supplementary Data 4, meaning that their contemporary distributions may not adequately characterize their likely fundamental niche, niche truncation indices did not influence model performance (all P > 0.14, Supplementary Data 3 tab ‘SDM-NTI’).

Comparison of mechanistic and invasive models

Mechanistic model capacity to correctly identify invasive bird occurrences was always higher than correlative model sensitivity (Fig. 1). Compared to the best performing SDMs, mechanistic forecasts accounting for intraspecific variation had significantly higher sensitivity values (0.96±0.06 for NicheMapper vs 0.70±0.37 for BART models, p-value = 0.047; versus 0.58±0.47 for GLM, p-value = 0.028; versus 0.37±0.45 for FNE, p-value < 0.0001), with broadly similar results for species-level mechanistic forecasts (0.79±0.24 for NicheMapper, p-value versus BART models is 0.14, versus GLM p-value = 0.012 and versus FNE p-value < 0.0001; Supplementary Data 3 tab ‘SDM vs NicheMapper sensitivity’). In terms of model specificity, intraspecific mechanistic models have a similar capacity to identify locations currently free from invasive bird species as GLM and BART models (0.36±0.22 for NicheMapper versus 0.33±0.38 for BART, p-value = 0.99; versus 0.47±0.46 for GLM, p-value = 0.53), but lower than FNE models (versus 0.79±0.28, p-value < 0.0001). Species-level mechanistic models had a model specificity to comparable to that of the FNE models (0.73±0.27 for NicheMapper, p-value compared against FNE is 0.75). Species-level mechanistic models had a higher specificity than GLM (p-value = 0.11) and BART models (p-value = 0.0004; Supplementary Data 3 tab ‘SDM vs NicheMapper specificity’).).

A limited number of key biophysical traits governs invasive range distributions. Latin Hypercube sampling was used to efficiently sample the functional parameter space, simultaneously varying the values of the input parameters. These sensitivity analyses, while accounting for invasion history, indicate that mechanistic model performance is most strongly influenced by basal metabolic rate, body mass and density, followed by feather length, feather depth and body temperature (Supplementary Figure 5). A PCA on these key biophysical traits yielded two relevant axes, whereby the first axis (82.2% of variation) was mainly influenced by basal metabolic rate (+0.50), body mass (+0.49), feather depth (0.49) and feather length (0.49). The second axis (17.1%) was dominated by body temperature only (+0.94). Mechanistic model sensitivity was higher for species characterized by higher body masses, higher basal metabolic rates, better plumage insulation capacity (biophysical PCA axis 1; p-value = 0.0027, z-value: 2.99) and a lower body temperature (biophysical PCA axis 2: p-value = 0.021, z-value: -2.31) while the opposite was true for model specificity (first PCA axis: p-value < 0.001, z-value: -8.89, second axis: p-value = 0.009, z-value: 2.61). Specificity also correlated negatively with propagule pressure (defined as the number of successful introductions across Europe, p-value = 0.0039, z-value: -2.89, Supplementary Figure 6).

Supplementary Methods 1 – Extended technical description of the NicheMapper ecophysiological platform.

The endotherm model requires information on a set of biophysical and behavioral species traits, which were either measured directly from museum specimens or derived from the literature. Morphological traits. Body dimensions of all species (i.e. shape, lengths and diameters of the beak, head, neck, torso and the legs) were measured based on museum specimens held at the Royal Belgian Institute of Natural Sciences (Brussels, Belgium). For each species, four specimens (two males and two females) were selected and measured using digital calipers and metal bird rulers. Body mass estimates were taken from the literature (Supplementary Table 3), and average bird density was estimated at 875 kg/m^3 based on [1,2]. Body fat was considered to be present subcutaneously on bird neck and torso, and percentages varied from 2.6 to 12.3 % (Psittaciformes: 2.6%[3], Sturnidae: 2.7%[4,5], Estrildidae, Ploceidae and Viduidae: 4.0 %[6], Phasianidae: 12.3 %[7,8]). Within the model, adjustments to morphometric measurements of each body part were made when model-calculated densities of body parts (flesh only, without feathers) differed by more than 5% from the 875 kg/m^3 estimate above[2]. These adjustments were allowed because body masses were taken from the literature while morphometric parameters were measured from museum specimens. Feather length, depth and solar reflectivity were measured dorsally and ventrally for each body part. Feather depth was measured using a small, blunt wooden probe that was inserted normal to the skin surface, with the probe lightly touching the skin, and then marking the probe with pencil where it visually disappears into the feathers. Feather length was obtained by inserting the wooden probe parallel to the lie of the feathers until it touches the skin, and marking the probe where it disappears into the feathers. Feather length and feather depth measurements were conducted separately for the head (1 location), neck (1 location) and torso (3 locations longitudinally), both ventrally and dorsally,

and were repeated twice (i.e. total of 20 measurements per museum specimen). Feather element diameter was considered to be 30 µm and element density was set at 10.000/cm²[9]. Using the same body locations as for the feather length and depth measurements, solar reflectivity was measured across 300–2100 nm using a dual spectrophotometer and light source setup (Avantes Inc., Broomfield, CO, USA): AvaSpec-2048 spectrometer (300–1100 nm) and AvaSpec-NIR256-2.0TEC (1000–2100 nm), and AvaLight-DH-S deuterium–halogen light source and AvaLight-HAL-S-MINI light source connected to a quadrifurcated fibre optic cable and held at 90° using a RPH-1 reflection probe holder [10], and calculating average reflectance values following[11]. Solar reflectivity of legs and beak was set at 0.33[2]. For those species that have (seasonally) sexually dimorphic plumages, reflectivity was calculated separately for males and females, assuming that outside of the breeding season, males had the same reflectivity as females. Thermal conductivity was set at 0.034 W/mC (www.engineeringtoolbox.com) and the point along the feather layer temperature profile where radiant heat exchange with the environment is modelled was set at 0.9 (0 = at the skin surface versus 1 = at the feather-air interface)[12]. Ecophysiological traits: body temperature (core, minima and maxima) [13–19] and basal metabolic rates (BMR, Supplementary Table 4) were taken from literature on the focal species or from closely related species (family level) where available. When no empirical measurements were available, body temperature was estimated from bird order level body temperatures summarized in [20] while BMR was estimated using allometric scaling relationships[21]. BMR multipliers for daily foraging activity were set at 2.5 based on [22–24], and as breeding bird energy expenditure is generally estimates at four times the BMR [25–27], an additional energetic multiplier of 1.5 was implemented to account for heat generated during breeding (see below). Muscle efficiency, i.e. the proportion of additional activity expenditure that contributes to the animal's heat balance, was assumed to be 25% [28] (meaning that of the chemical energy used, 75% is dissipated as heat). Flesh specific heat capacity was modelled at

4185 J/Kg.C [29] while flesh thermal conductivity was allowed to vary between 0.41 and 2.80 W/m.C[30] (for simulating vasodilation or vasoconstriction). Assuming an ellipsoid torso shape, the temperature differential between body core and skin was calculated based on equation (1) in [31], exhaled air was assumed to be 2°C warmer than local air temperature at each hour [12,32], and oxygen extraction efficiency [33] (which is used to simulate panting) was allowed to vary between 6 and 30% [34–36]. While birds cannot sweat, for cutaneous water loss, we estimated that 1% of the skin functioned as a free water surface to account for the eyes and thin skin, and assumed that effective percent wet skin can rise up to 5% under heat stress[37]. Birds were also allowed to increase plumage depth to simulate ptiloerection under cold stress. Behavioral traits: to preserve homeothermy, birds were assumed to first employ ecophysiological responses before activating behavioral modifications. For a bird at risk of hyperthermia, the order of ecophysiological and behavioral changes was as follows: increase flesh thermal conductivity to maximum, increase core temperature to maximum, pant by reducing oxygen efficiency to minimum, 'sweat' (i.e. subcutaneous water loss, see above), fly to a higher position (assuming perches were always available) and ultimately seek shade (assuming shade was always available and that birds remain active while in shaded areas). For birds at risk of hypothermia this is: increase feather insulation capacity through ptiloerection, reduction of flesh thermal conductivity, reduction of core body temperature to the minimum, and finally changing their posture to reduce surface area (i.e. 'curling up' to a 1:1 'single lump' shape, tucking the beak, head, neck and torso into a single object and only keeping the legs exposed to the environment[38]). All birds were considered to be active during diurnal and crepuscular periods[39].

Supplementary References

1. Seamans, T., Hamershock, D. & Bernhardt, G. Determination of body density for twelve bird species. *Ibis (Lond. 1859).* **137**, 424–428 (1995).
2. Fitzpatrick, M. J., Mathewson, P. D. & Porter, W. P. Validation of a Mechanistic Model for Non-Invasive Study of Ecological Energetics in an Endangered Wading Bird with Counter-Current Heat Exchange in its Legs. *PLoS One* **10**, e0136677 (2015).
3. Ramesh, A. *et al.* Characteristic trend of persistent organochlorine contamination in wildlife from a tropical agricultural watershed, South India. *Arch. Environ. Contam. Toxicol.* **23**, 26–36 (1992).
4. Meijer, T., Mohring, F. J. & Trillmich, F. Annual and Daily Variation in Body Mass and Fat of Starlings Sturnus vulgaris. *J. Avian Biol.* **25**, 98 (1994).
5. Christians, J. K. & Williams, T. D. Effects of exogenous 17(β)-estradiol on the reproductive physiology and reproductive performance of european starlings (Sturnus vulgaris). *J. Exp. Biol.* **202**, (1999).
6. Ward, P. Seasonal and Diurnal Changes in the Fat Content of an Equatorial Bird. *Physiol. Zool.* **42**, 85–95 (1969).
7. Barrett, M. W. & Bailey, E. D. Influence of Metabolizable Energy on Condition and Reproduction of Pheasants. *J. Wildl. Manage.* **36**, 12 (1972).
8. Draycott, R. A. H., Hoodless, A. N., Ludiman, M. N. & Robertson, P. A. Effects of Spring Feeding on Body Condition of Captive-Reared Ring-Necked Pheasants in Great Britain. *J. Wildl. Manage.* **62**, 557 (1998).
9. Kearney, M. R., Porter, W. P. & Murphy, S. A. An estimate of the water budget for the endangered night parrot of Australia under recent and future climates. *Clim. Chang. Responses* **3**, (2016).

10. Shawkey, M. D. *et al.* Beyond colour: consistent variation in near infrared and solar reflectivity in sunbirds (Nectariniidae). *Naturwissenschaften* **104**, 78 (2017).

11. Smith, K. R. *et al.* Colour change on different body regions provides thermal and signalling advantages in bearded dragon lizards. *Proc. R. Soc. B Biol. Sci.* **283**, 20160626 (2016).

12. Porter, W. P., Budaraju, S., Stewart, W. E. & Ramankutty, N. Calculating Climate Effects on Birds and Mammals: Impacts on Biodiversity, Conservation, Population Parameters, and Global Community Structure1. *https://doi.org/10.1668/0003-1569(2000)040[0597:CCEOBA]2.0.CO;2* **40**, 597–630 (2000).

13. Johnson, S. R. & Cowan, I. M. The energy cycle and thermal tolerance of the starlings (Aves, Sturnidae) in North America. *Can. J. Zool.* **53**, 55–68 (1975).

14. Thabethe, V., Thompson, L. J., Hart, L. A., Brown, M. & Downs, C. T. Seasonal effects on the thermoregulation of invasive rose-ringed parakeets (Psittacula krameri). *J. Therm. Biol.* **38**, 553–559 (2013).

15. Weathers, W. W. & Caccamise, D. F. Seasonal acclimatization to temperature in monk parakeets. *Oecologia* **35**, 173–183 (1978).

16. Bucher, T. L. & Morgan, K. R. The effect of ambient temperature on the relationship between ventilation and metabolism in a small parrot (Agapornis roseicollis). *J. Comp. Physiol. B* **159**, 561–567 (1989).

17. Cade, T., Tobin, C. & Gold, A. Water economy and metabolism of two Estrildine finches. *Physiol. Zool.* **38**, 9–33 (1965).

18. Luo, Y. *et al.* Metabolism and thermoregulation between Mrs Hume's Pheasant (Syrmaticus humiae) and Elliot's Pheasant (S. ellioti). doi:10.3724/SP.J.1141.2011.04396.

19. Dmi'el, R. & Tel-Tzur, D. Heat balance of two starling species (Sturnus vulgaris and

Onychognathus tristrami) from temperate and desert habitats. *J. Comp. Physiol. B* **155**, 395–402 (1985).

20. Prinzinger, R., Preßmar, A. & Schleucher, E. Body temperature in birds. *Comparative Biochemistry and Physiology -- Part A: Physiology* vol. 99 499–506 (1991).

21. Bushuev, A., Tolstenkov, O., Zubkova, E., Solovyeva, E. & Kerimov, A. Basal metabolic rate in free-living tropical birds: the influence of phylogenetic, behavioral, and ecological factors. *Curr. Zool.* **64**, 33–43 (2018).

22. Magrath, R. & Lill, A. The use of time and energy by the Crimson rosella in a temperate wet forest in winter. *Aust. J. Zool.* **31**, (1983).

23. Beggs, J. R. & Wilson, P. R. The kaka Nestor meridionalis, a New Zealand parrot endangered by introduced wasps and mammals. *Biol. Conserv.* **56**, 23–38 (1991).

24. Root, T. Environmental Factors Associated with Avian Distributional Boundaries. *J. Biogeogr.* **15**, 489 (1988).

25. Stevenson, I. R. & Bryant, D. M. Avian phenology: Climate change and constraints on breeding. *Nature* **406**, 366–367 (2000).

26. Drent, R. H. & Daan, S. The prudent parent: energetic adjustments in avian breeding. *Ardea* **55**, 225–252 (1980).

27. Ricklefs, R. E. & Williams, J. B. Daily Energy Expenditure and Water-Turnover Rate of Adult European Starlings (Sturnus vulgaris) during the Nesting Cycle. *Auk* **101**, 707–716 (1984).

28. Lovvorn, J. . Thermal substitution and aerobic efficiency: measuring and predicting effects of heat balance on endotherm diving energetics. *Philos. Trans. R. Soc. B Biol. Sci.* **362**, 2079–2093 (2007).

29. Kearney, M. R., Simpson, S. J., Raubenheimer, D. & Kooijman, S. A. L. M. Balancing heat, water and nutrients under environmental change: a thermodynamic niche

framework. *Funct. Ecol.* **27**, 950–966 (2013).

30. Chato, J. Advanced heat transfer. in *Heat transfer in bioengineering* (ed. Chao, B.) 395–412 (Urbana: University of Illinois, 1969).

31. Porter, W., Munger, J., Stewart, W., Budaraju, S. & Jaeger, J. Endotherm Energetics - From a Scalable Individual-Based Model to Ecological Applications. *Aust. J. Zool.* **42**, 125 (1994).

32. Fort, J., Porter, W. P. & Grémillet, D. Thermodynamic modelling predicts energetic bottleneck for seabirds wintering in the northwest Atlantic. *J. Exp. Biol.* **212**, 2483–90 (2009).

33. Zhang, Y., Mathewson, P. D., Zhang, Q., Porter, W. P. & Ran, J. An ecophysiological perspective on likely giant panda habitat responses to climate change. *Glob. Chang. Biol.* **24**, 1804–1816 (2018).

34. Arens, J. R. & Cooper, S. J. Seasonal and Diurnal Variation in Metabolism and Ventilation in House Sparrows. *Condor* **107**, 433–444 (2005).

35. Bucher, T. L. Oxygen consumption, ventilation and respiratory heat loss in a parrot, Bolborhynchus lineola, in relation to ambient temperature. *J. Comp. Physiol.* ■ *B* **142**, 479–488 (1981).

36. Bucher, T. L. Ventilation and oxygen consumption in Amazona viridigenalis - A reappraisal of 'resting' respiratory parameters in birds. *J. Comp. Physiol. B* **155**, 269–276 (1985).

37. Porter, W. P., Vakharia, N., Klousie, W. D. & Duffy, D. Po'ouli landscape bioinformatics models predict energetics, behavior, diets, and distribution on Maui. *Integr. Comp. Biol.* **46**, 1143–1158 (2006).

38. Kearney, M. & Porter, W. Mechanistic niche modelling: Combining physiological and spatial data to predict species' ranges. *Ecol. Lett.* **12**, 334–350 (2009).

39. Mathewson, P. D. *et al.* Experimental and modeled thermoregulatory costs of repeated sublethal oil exposure in the Double-crested Cormorant, Phalacrocorax auritus. *Mar. Pollut. Bull.* **135**, 216–223 (2018).

Supplementary Table 1 – GBIF data sources

species	***date downloaded***	***sources***
Acridotheres cristatellus	8 February 2023	10.15468/dl.ustqqc
Acridotheres tristis	8 February 2023	10.15468/dl.b4zahe
Agapornis fischeri	8 February 2023	10.15468/dl.5n4zsb
Agapornis personatus	8 February 2023	10.15468/dl.ssbk5f
Agapornis roseicollis	8 February 2023	10.15468/dl.wu9ayj
Amandava amandava	8 February 2023	10.15468/dl.524449
Aratinga acuticaudata	8 February 2023	10.15468/dl.eb4bh6
Chrysolophus pictus	8 February 2023	10.15468/dl.825xfb
Estrilda astrild	8 February 2023	10.15468/dl.xmp8gj
Estrilda melpoda	8 February 2023	10.15468/dl.j2xqk8
Estrilda troglodytes	8 February 2023	10.15468/dl.46yfp6
Euplectes afer	8 February 2023	10.15468/dl.6ympyy
Myiopsitta monachus	8 February 2023	10.15468/dl.exku3s
Nandayus nenday	8 February 2023	10.15468/dl.r4a6dm
Ploceus melanocephalus	8 February 2023	10.15468/dl.a3kede
Poicephalus senegalus	8 February 2023	10.15468/dl.668w64
Psittacula eupatria	8 February 2023	10.15468/dl.jv55pg
Psittacula krameri	8 February 2023	10.15468/dl.raah5v
Syrmaticus reveesii	8 February 2023	10.15468/dl.qaxtnh
Vidua macroura	8 February 2023	10.15468/dl.8mkp8w

Supplementary Table 2 – ODMAP reporting protocol for SDM

**ODMAP Reporting Protocol for SDM**			
**section**	**subsection**	**element**	**Value**
Overview	Authorship	Study title	Mechanistic models project bird invasions with accuracy
Overview	Authorship	Author names	Diederik Strubbe, Laura Jiménez, A. Márcia Barbosa, Amy J.S. Davis, Luc Lens, Carsten Rahbek
Overview	Authorship	Contact	diederik.strubbe@ugent.be
Overview	Authorship	Study link	https://github.com/LauraJim/Modeling_bird_invasions
Overview	Model objective	Model objective	Testing the accuracy with which native-range based SDM can predict invasive bird occurrences across Europe.
Overview	Model objective	Target output	Geographical maps of Europe indicating whether a given location (pixel) is climatically suitable for a set of 20 non-native introduced bird species.
Overview	Focal Taxon	Focal Taxon	Aves
Overview	Location	Location	Europe (model forecasts), global (training regions for native range SDMs)
Overview	Scale of Analysis	Spatial extent	NA (cfr. global), Europe: -10.66667, 33, 34.75, 71.25 (xmin, xmax, ymin, ymax)
Overview	Scale of Analysis	Spatial resolution	~10 km²
Overview	Scale of Analysis	Temporal extent	Climate: 1970-2000, habitat: 2015-2019, occurrence data: 1960-2019
Overview	Scale of Analysis	Temporal resolution	Climate data were averaged over the 30 year period, habitat data were averaged over the 5-year period for which they were available.
Overview	Scale of Analysis	Boundary	Coord. ref. : lon/lat WGS 84 (EPSG:4326)
Overview	Biodiversity data	Observation type	Volunteer/citizen-science based survey data
Overview	Biodiversity data	Response data type	Presence-only
Overview	Predictors	Predictor types	Climatic variables, habitat variables, variables representing (resource-related) biotic interaction variables.
Overview	Hypotheses	Hypotheses	We test the hypothesis that the climates that species currently occupy across their native range can be used to accurately predict in which non-native areas they can potentially invade.
Overview	Assumptions	Model assumptions	We assume that across their native ranges, species are in pseudo-equilibrium with their environment. We also assume that available occurrence data are representative

			of species' contemporary distributions, and that any biases are adequately accounted for).
Overview	Algorithms	Modelling techniques	**Fundamental niche ellipses** (FNE, Jiménez, L. & Soberón, J. 2020 Estimating the fundamental niche: Accounting for the uneven availability of existing climates in the calibration area. Ecol. Modell. 464, 109823).<br>**Bayesian Additive Regression Trees** (BART, Chipman, H. A., George, E. I. & McCulloch, R. E. 2010 BART: Bayesian additive regression trees. Ann. Appl. Stat. 4, 266–298).<br>**Generalized Linear Models** (GLM, McCullagh, P. & Nelder, J. A. Generalized Linear Models. 2019 Regression Analysis with Application G.B. Wetherill, Routledge.)
Overview	Algorithms	Model complexity	NA, default settings
Overview	Algorithms	Model averaging	No model averaging was performed
Overview	Workflow	Model workflow	(1) clean GBIF occurrence data , (2) summarize all 19 bioclim variables into two PCA axes, (3) train models on species' native ranges using spatial cross-validation, (4) forecast models to Europe to obtain predictions of invasion risk using (a) extrapolation and (b) clamping, (5) use independent invasive range (i.e. European) occurrence data to evaluate accuracy of native-range based predictions. This workflow was then repeated for the inclusion of habitat and biotic interaction variables in the models.
Overview	Software	Software	R version 4.2.1. Core SDM packages:<br>**library(embarcadero)**<br>Carlson C (2022). _embarcadero: Species distribution models with BART_. R package version 1.2.0.1003.<br>**library(stats)**<br>R Core Team (2022). R: A language and environment for statistical computing. R Foundation for Statistical Computing, Vienna, Austria. URL https://www.R-project.org/.<br>**library(modEvA)**<br>Barbosa, A.M., Real, R., Munoz, A.R. & Brown, J.A. (2013). New measures for assessing model equilibrium and prediction mismatch in species distribution models. Diversity and Distributions, 19(10), 1333-1338.<br>**library(blockCV)**

			Valavi R, Elith J, Lahoz-Monfort JJ, Guillera-Arroita G. blockCV: An R package for generating spatially or environmentally separated folds for k-fold cross-validation of species distribution models. Methods Ecol Evol. 2019; 10:225–232.<br>**library(kuenm)**<br>Cobos ME, Peterson AT, Barve N, Osorio-Olvera L. (2019) kuenm: an R package for detailed development of ecological niche models using Maxent PeerJ, 7:e6281<br>**Fundamental niche ellipses**:<br>Functions 'niche.G', 'GE.space', 'negloglike' and 'fitNiche' available at https://doi.org/10.1016/j.ecolmodel.2021.109823<br>**library(CoordinateCleaner)**<br>Zizka A, Silvestro D, Andermann T, Azevedo J, Duarte Ritter C, Edler D, Farooq H, Herdean A, Ariza M, Scharn R, Svanteson S, Wengstrom N, Zizka V, Antonelli A (2019). "CoordinateCleaner: standardized cleaning of occurrence records from biological collection databases." _Methods in Ecology and Evolution_, -7. doi:10.1111/2041-210X.13152 <https://doi.org/10.1111/2041-210X.13152>, R package version 2.0-20, <https://github.com/ropensci/CoordinateCleaner>.
Overview	Software	Code availability	https://github.com/LauraJim/Modeling_bird_invasions
Overview	Software	Data availability	https://github.com/LauraJim/Modeling_bird_invasions
Data	Biodiversity data	Taxon names	(*Acridotheres cristatellus, Acridotheres tristis, Agapornis fischeri, Agapornis personatus, Agapornis roseicollis, Amandava amandava, Aratinga acuticaudata, Chrysolophus pictus, Estrilda astrild, Estrilda melpoda, Estrilda troglodytes, Euplectes afer, Myiopsitta monachus, Nandayus nenday, Ploceus melanocephalus, Poicephalus senegalus, Psittacula eupatria, Psittacula krameri, Syrmaticus reveesi, Vidua macroura)*
Data	Biodiversity data	Taxonomic reference system	The Integrated Taxonomic Information System, DOI: 10.15468/kdu5gg
Data	Biodiversity data	Ecological level	species
Data	Biodiversity data	Data sources	https://www.gbif.org/
Data	Biodiversity data	Sampling design	opportunistic; volunteer-based recording schemes
Data	Biodiversity data	Sample size	After rarefying at 50km resolution: *Acridotheres cristatellus (7), Acridotheres tristis (43), Agapornis fischeri (32), Agapornis personatus (19), Agapornis roseicollis (67), Amandava amandava (295), Aratinga acuticaudata (100), Chrysolophus pictus (19), Estrilda astrild (789), Estrilda melpoda (200), Estrilda troglodytes(90), Euplectes afer (302), Myiopsitta monachus (464), Nandayus nenday (40), Ploceus*

			melanocephalus (118), Poicephalus senegalus (129), Psittacula eupatria (288), Psittacula krameri (835), Syrmaticus reveesi (24), Vidua macroura (890)
fo	Biodiversity data	Clipping	Native range background areas were here obtained by applying a spatial buffer surrounding available rarefied occurrences, with a radius equaling the mean pairwise great-circle distance (calculated through the 'geobuffer' R package) between occurrences, and clipped to the zoogeographical regions where the species is native to account for major biogeographical barriers to dispersal.
Data	Biodiversity data	Scaling	One occurrence record per 10km pixel, rarefaction of occurrences using a 50 km distance.
Data	Biodiversity data	Cleaning	GBIF occurrences (a) were retained when they were located within the species' natural distribution range, as given by BirdLife's extent of occurrence digital maps, which were buffered with distance of 0.5° to reduce potential errors associated with georeferencing and digitalization procedures, and (b) we excluded occurrences in those areas occupied only during the non-breeding season or during migration. To identify potentially problematic occurrence data, we used the CoordinateCleaner R package.
Data	Biodiversity data	Absence data	NA
Data	Biodiversity data	Background data	Accessible but not occupied locations (pixels) were considered as background area/(pseudo)absences.
Data	Biodiversity data	Errors and biases	Native-range occurrence data: restricted to areas corresponding with BirdLife extent of occurrence maps, invasive range occurrence data: to minimize the risk of including species observations that do not reflect established, self-sustaining populations, occurrences were only retained when literature sources confirmed the presence of established breeding populations. Model evaluation threshold were varies from 2.5% to 5% to account for possible remaining occurrence data errors.
Data	Data partitioning	Training data	five-fold spatial block cross-validation resampling was used to obtain native range training and validation data
Data	Data partitioning	Validation data	five-fold spatial block cross-validation resampling was used to obtain native range training and validation data
Data	Data partitioning	Test data	Invasive range occurrence data were used as independent test data
Data	Predictor variables	Predictor variables	19 bioclimatic variables summarized into two PCA axes
Data	Predictor variables	Data sources	Fick, S.E. and R.J. Hijmans, 2017. WorldClim 2: new 1km spatial resolution climate surfaces for global land areas. International Journal of Climatology 37 (12): 4302-4315.

Data	Predictor variables	Spatial extent	NA (cfr. global), Europe: -10.66667, 33, 34.75, 71.25 (xmin, xmax, ymin, ymax)
Data	Predictor variables	Spatial resolution	10 km
Data	Predictor variables	Coordinate reference system	lon/lat WGS 84 (EPSG:4326)
Data	Predictor variables	Temporal extent	1970-2000
Data	Predictor variables	Temporal resolution	Climate data were averaged over the 30 year period
Data	Predictor variables	Data processing	NA
Data	Predictor variables	Errors and biases	NA
Data	Predictor variables	Dimension reduction	PCA on all 19 bioclimatic variables
Data	Transfer data	Data sources	Fick, S.E. and R.J. Hijmans, 2017. WorldClim 2: new 1km spatial resolution climate surfaces for global land areas. International Journal of Climatology 37 (12): 4302-4315.
Data	Transfer data	Spatial extent	Europe: -10.66667, 33, 34.75, 71.25 (xmin, xmax, ymin, ymax)
Data	Transfer data	Spatial resolution	10 km
Data	Transfer data	Temporal extent	lon/lat WGS 84 (EPSG:4326)
Data	Transfer data	Temporal resolution	1970-2000
Data	Transfer data	Models and scenarios	Climate data were averaged over the 30 year period
Data	Transfer data	Data processing	NA
Data	Transfer data	Quantification of Novelty	MESS analysis to identify areas of strict interpolation
Model	Variable pre-selection	Variable pre-selection	PCA on all 19 bioclimatic variables
Model	Multicollinearity	Multicollinearity	NA (see above, PCA axes were used to model)
Model	Model settings	Model settings (fitting)	defaults
Model	Model settings	Model settings (extrapolation)	We used both ‘extrapolation’ and ‘clamping’, using the ‘clamp.vars’ function of the R package ‘ENMeval’
Model	Model estimates	Coefficients	Assessed through 5-fold cross validation
Model	Model estimates	Parameter uncertainty	Assessed through 5-fold cross validation
Model	Model estimates	Variable importance	Not assessed

Model	Model selection - model averaging - ensembles	Model selection	No formal model selection, after cross-validation, all native range occurrences were combined with the two PCA climate variables to generate predictions.
Model	Model selection - model averaging - ensembles	Model averaging	NA
Model	Model selection - model averaging - ensembles	Model ensembles	NA
Model	Analysis and Correction of non-independence	Spatial autocorrelation	Occurrences were rarefied at a ~50 km resolution to minimize the influence of spatial autocorrelation, spatial blocking for cross-validation was applied via the blockCV R package.
Model	Analysis and Correction of non-independence	Temporal autocorrelation	NA
Model	Analysis and Correction of non-independence	Nested data	NA
Model	Threshold selection	Threshold selection	We applied a both a 2.5 and 2.5% training omission threshold ('E'), based on the native range occurrence data used to calibrate the models.
Assessment	Performance statistics	Performance on training data	AUCratio, sensitivity, specificity
Assessment	Performance statistics	Performance on validation data	AUCratio, sensitivity, specificity
Assessment	Performance statistics	Performance on test data	AUCratio, sensitivity, specificity
Assessment	Plausibility check	Response shapes	Not assessed
Assessment	Plausibility check	Expert judgement	Yes: model predicting that small-bodied (sub)tropical birds introduced to Europe can find climatically suitable habitat in the high Artic were considered of debatable reliability.
Prediction	Prediction output	Prediction unit	presence and absences (i.e. climatically suitable or not).
Prediction	Prediction output	Post-processing	Model predictive performance was assessed an (a) the whole of Europe, and (b) excluding areas of strict extrapolation as identified by a MESS analysis.
Prediction	Uncertainty quantification	Algorithmic uncertainty	Three different SDM methods were used on this study.
Prediction	Uncertainty quantification	Input data uncertainty	NA
Prediction	Uncertainty quantification	Parameter uncertainty	Assessed through 5-fold cross validation

Prediction	Uncertainty quantification	Scenario uncertainty	
Prediction	Uncertainty quantification	Novel environments	Model predictive performance was assessed an (a) the whole of Europe, and (b) excluding areas of strict extrapolation as identified by a MESS analysis.

Supplementary Table 3 – Body mass data

species	Sample size (# of individuals)
Acridotheres cristatellus	49
Acridotheres tristis	170
Agapornis fischeri	21
Agapornis personatus	31
Agapornis roseicollis	51
Amandava amandava	15
Aratinga acuticaudata	17
Chrysolophus pictus	15
Estrilda astrild	184
Estrilda melpoda	114
Estrilda troglodytes	386
Euplectes afer	44
Myiopsitta monachus	450
Nandayus nenday	16
Ploceus melanocephalus	35
Poicephalus senegalus	17
Psittacula eupatria	335
Psittacula krameri	698
Syrematicus reveesii	618
Vidua macroura	79

Supplementary References

1. Dunning, J. J. *CRC Handbook of Avian Body Masses, 2nd Edition*. CRC Press, Boca raton. (2007).

2. Johnson, S. R. & Cowan, I. M. The energy cycle and thermal tolerance of the starlings (Aves, Sturnidae) in North America. *Can. J. Zool.* **53**, 55–68 (1975).

3. Burton, S., Perrin, M. R. & Downs, C. T. Thermal biology of African lovebirds and Australian grass parakeets. *J. Therm. Biol.* **33**, 355–362 (2008).

4. Koves Hrabar, H. D. N. & Perrin, M. The effect of bill structure on seed selection by granivorous birds. *African Zool.* **37**, 67–80 (2002).

5. Godon, J. J., Arulazhagan, P., Steyer, J. P. & Hamelin, J. Vertebrate bacterial gut diversity: Size also matters. *BMC Ecol.* **16**, 12 (2016).

6. McNab, B. K. Ecological factors affect the level and scaling of avian BMR. *Comp. Biochem. Physiol. Part A Mol. Integr. Physiol.* **152**, 22–45 (2009).

7. Iwaniuk, A. N., Dean, K. M. & Nelson, J. E. Interspecific Allometry of the Brain and Brain Regions in Parrots (*Psittaciformes*): Comparisons with Other Birds and Primates. *Brain. Behav. Evol.* **65**, 40–59 (2005).

8. Bucher, T. L. & Morgan, K. R. The effect of ambient temperature on the relationship between ventilation and metabolism in a small parrot (*Agapornis roseicollis*). *J. Comp. Physiol. B* **159**, 561–567 (1989).

9. Mohanty, B., Pandey, S. P. & Tsutsui, K. Thyroid disrupting pesticides impair the hypothalamic-pituitary-testicular axis of a wildlife bird, *Amandava amandava*. *Reprod. Toxicol.* **71**, 32–41 (2017).

10. Sullivan, M. J. P., Davies, R. G., Mossman, H. L. & Franco, A. M. A. An Anthropogenic Habitat Facilitates the Establishment of Non-Native Birds by Providing Underexploited Resources. *PLoS One* **10**, e0135833 (2015).

11. Iwaniuk, A. N., Gutierrez-Ibanez, C., Pakan, J. M. P. & Wylie, D. R. Allometric Scaling of the Tectofugal Pathway in Birds. *Brain. Behav. Evol.* **75**, 122–137 (2010).

12. Forys, E. A. & Allen, C. R. Biological invasions and deletions: Community change in south Florida. *Biol. Conserv.* **87**, 341–347 (1998).

13. Cabezas, S., Carrete, M., Tella, J. L., Marchant, T. A. & Bortolotti, G. R. Differences in acute stress responses between wild-caught and captive-bred birds: A physiological mechanism contributing to current avian invasions? *Biol. Invasions* **15**, 521–527 (2013).

14. McDonald, S. Normal weight of pet birds. http://www.scottemcdonald.com/pdfs/AverageWeights.pdf (2020).

15. Feslka-Blaszczyk, L. & Phorecki, K. Comparison of conformation and laying performance of various pheasant species. *Acta Sci. Pol. Zootech.* **14**, 39–108 (2015).

16. Barnard, P. Ageing and sexing Common waxbill Estrilda astrild. *Safring News* **17**, 79–83 (1988).

17. Akinpelu, A. Body mass and moult cycles in adult orange-cheeked waxbill *Estrilda melpoda* in Ile-Ife, Nigeria. *Ostrich* **68**, 119–120 (1997).

18. Stephens, C. M., Siegel, R. B. & Weathers, W. W. Thermal conductance and basal metabolism of the orange-cheeked waxbill (*Estrilda melpoda*). *Ostrich* **72**, 121–123 (2001).

19. Payne, R., Bonan, A. & Kirwan, G. Common waxbill (Estrilda astrild). In *Birds of the World* (eds. del Hoyo, J., Elliot, A., Sargatal, J., Christie, D. & de Juana, E.) (2020).

20. Weathers, W. W. & Nagy, K. A. Daily energy expenditure and water flux in black-rumped waxbills (*Estrilda troglodytes*). *Comp. Biochem. Physiol. -- Part A Physiol.* **77**, 453–458 (1984).

21. Yarbrough, C. G. The influence of distribution and ecology on the thermoregulation of small birds. *Comp. Biochem. Physiol. -- Part A Physiol.* **39**, 235–266 (1971).

22. Kendeigh, S. C. Tolerance of Cold and Bergmann's Rule. *Auk* **86**, 13–25 (1969).

23. Bennettand, P. M. & Harvey, P. H. Active and resting metabolism in birds: allometry, phylogeny and ecology. *J. Zool.* **213**, 327–344 (1987).

24. Cox, D.T.C. & Cresswell, W. Mass gained during breeding positively correlates with adult survival because both reflect life history adaptation to seasonal food availability. *Oecologia* **174**, 1197–1204 (2014).

25. Cox, D.T.C. *et al.* Patterns of seasonal and yearly mass variation in West African tropical savannah birds. *Ibis* **153**, 672–683 (2011).

26. Payne, R. Black-rumped waxbill *Estrilda troglodytes*. In *Birds of the World2* (eds. del Hoyo, J., Elliott, A., Sargatal, J., Christie, D. & de Juana, E.) (2020).

27. McGregor, R. Survival rates and seasonality of tropical birds. PhD thesis, University of St Andrews, 227p (2005).

28. Dangoisse, G. Etude de la population de conures veuves (*Myiopsitta monachus*) de Bruxelles-Capital. *Aves* **46**, 57–69 (2009).

29. Eberhard, J. Breeding biology of the monk parakeet. *Wilson Bull.* **110**, 463–473 (1998).

30. Humphrey, P. S. & Peterson, R. T. Nesting behavioir and affinities of monk parakeets of southern Buenos Aires Province, Argentina. *Wilson Bull* vol. 90 (1978).

31. Pollock, C. Basic information sheet: Conure. https://lafeber.com/vet/basic-information-sheet-for-the-conure/ (2012).

32. Sanz-Aguilar, A., Carrete, M., Edelaar, P., Potti, J. & Tella, J. L. The empty temporal niche: breeding phenology differs between coexisting native and invasive birds. *Biol. Invasions* **17**, 3275–3288 (2015).

33. Wanmi, N., Mohammed, A. & Nev, T. Morphometric Study on Some Body Organs of the Wild African Senegal Parrot (*Poicephalus senegalus versteri*). *J. Vet. Anat.* **8**, 81–88 (2015).

34. Godon, J. J., Arulazhagan, P., Steyer, J. P. & Hamelin, J. Vertebrate bacterial gut diversity: Size also matters. *BMC Ecol.* **16**, 12 (2016).

35. Perrin, M. Niche separation in African parrots. In *Proceedings of the 12th Pan-African Ornithological Congress* (eds. Harebottle, D., Craig, A., Anderson, M., Rakotomanana, H. & Muchai, M.), pp. 9–37 (2009).

36. Johnson, S. Alexandrine parakeets. *Beauty of birds* https://www.beautyofbirds.com/alexandrineparakeet.html (2011).

37. Anonymous. Alexandrine parakeets. *ParrotFeather* http://parrotfeather.com/asiatics/alexandrine/ (2020).

38. Aubin, S. Species profile: Alexandrine parakeet. *Hari Hagen Avicultural Research Institute* https://hari.ca/avian-care/bird-health-booklets-species-profiles/species-profile-alexandrine-parakeet/ (2020).

39. Anonymous. Alexandrine parakeet fact sheet. *Northern Parrots* https://www.northernparrots.com/alexandrine-parakeet-fact-sheet-blog143/ (2013).

40. Anonymous. The Alexandrine parrot/Alexandrine parakeet. *AllPetBirds* https://www.allpetbirds.com/alexandrine-parrot (2020).

41. Kendeigh, S. C. Energy Requirements for Existence in Relation to Size of Bird. *Condor* **72**, 60–65 (1970).

42. Beklova, M., Pikula, J. Selected morphometric characteristics of Syrmaticus reevesii (Gray 1823) during the hunting season. *Folia Venatoria - Polovnicky Zb. - Mysliv. Sb. (Slovak Republic)* (1997).

43. Anonymous. Pheasant & Reeve's Pheasant. *Shooting Enterprise* http://www.shootingenterprise.com/pheasant-reeves-pheasant (2010).

44. McNab, B. K. Ecological factors affect the level and scaling of avian BMR. *Comp. Biochem. Physiol. - A Mol. Integr. Physiol.* **152**, 22–45 (2009).

Supplementary Table 4 – Basal Metabolic Rate data

species	sources
Acridotheres cristatellus	[1–3]
Acridotheres tristis	[4]
Agapornis fischeri	[5,6]
Agapornis personatus	[5]
Agapornis roseicollis	[5,6]
Amandava amandava	Estimated based on [7–16]
Aratinga acuticaudata	Estimated based on [17]
Chrysolophus pictus	[18]
Estrilda astrild	Estimated based on [7–16]
Estrilda melpoda	[7,13,14]
Estrilda troglodytes	[9,11,12,15,19,20]
Euplectes afer	Estimated based on [17]
Myiopsitta monachus	[21,22]
Nandayus nenday	Estimated based on [17]
Ploceus melanocephalus	Estimated based on [17]
Poicephalus senegalus	Estimated based on [23]
Psittacula eupatria	Estimated based on [24]
Psittacula krameri	[24]
Syrmaticus reveesii	[18]
Vidua macroura	Estimated based on [7–16]

References

1. McKechnie, A. E. & Wolf, B. O. The allometry of avian basal metabolic rate: Good predictions need good data. *Physiol. Biochem. Zool.* **77**, 502–521 (2004).

2. Atanasov, A. T. The near to linear allometric relationship between the total metabolic energy per life span and the body mass of Aves. *J. Anim. Vet. Adv.* **7**, 425–432 (2008).

3. Johnson, S. R. & Cowan, I. M. The energy cycle and thermal tolerance of the starlings (Aves, Sturnidae) in North America. *Can. J. Zool.* **53**, 55–68 (1975).

4. Bech, C., Chappell, M. A., Astheimer, L. B., Londoño, G. A. & Buttemer, W. A. A 'slow pace of life' in Australian old-endemic passerine birds is not accompanied by low basal metabolic rates. *J. Comp. Physiol. B* **186**, 503–512 (2016).

5. Burton, S., Perrin, M. R. & Downs, C. T. Thermal biology of African lovebirds and Australian grass parakeets. *J. Therm. Biol.* **33**, 355–362 (2008).

6. McNab, B. K. Ecological factors affect the level and scaling of avian BMR. *Comp. Biochem. Physiol. Part A Mol. Integr. Physiol.* **152**, 22–45 (2009).

7. Aschoff, J. & Pohl, H. Rhytmic variations in energy metabolism. *Fed. Proc.* **29**, 1541–1552 (1970).

8. Bennettand, P. M. & Harvey, P. H. Active and resting metabolism in birds: allometry, phylogeny and ecology. *J. Zool.* **213**, 327–344 (1987).

9. Cade, T., Tobin, C. & Gold, A. Water economy and metabolism of two Estrildine finches. *Physiol. Zool.* **38**, 9–33 (1965).

10. Gupta, B. B. P. & Thapliyal, J. P. Role of thyroid and testicular hormones in the regulation of

basal metabolic rate, gonad development, and body weight of spotted munia, Lonchura punctulata. *Gen. Comp. Endocrinol.* **56**, 66–69 (1984).

11. Lasiewski, R. C., Hubbard, S. & Moberly, W. Energetic relationships of a very small passerine bird. *Condor* **66**, 212–220 (1964).

12. Lasiewski, R. C. & Dawson, W. R. A Re-Examination of the Relation between Standard Metabolic Rate and Body Weight in Birds. *Condor* **69**, 13–23 (1967).

13. Stephens, C. M., Siegel, R. B. & Weathers, W. W. Thermal conductance and basal metabolism of the orange-cheeked waxbill (*Estrilda melpoda*). *Ostrich* **72**, 121–123 (2001).

14. Tieleman, B. I. & Williams, J. B. The adjustment of avian metabolic rates and water fluxes to desert environments. *Physiol. Biochem. Zool.* **73**, 461–479 (2000).

15. Weathers, W. W. & Nagy, K. A. Daily energy expenditure and water flux in black-rumped waxbills (*Estrilda troglodytes*). *Comp. Biochem. Physiol. Mol. Integr. Physiol.* **77**, 453–458 (1984).

16. Yarbrough, C. The influence of distribution and ecology on the thermoregulation of small birds. *Comp. Biochem. Physiol.* **39**, 235–266 (1971).

17. Bushuev, A., Tolstenkov, O., Zubkova, E., Solovyeva, E. & Kerimov, A. Basal metabolic rate in free-living tropical birds: the influence of phylogenetic, behavioral, and ecological factors. *Curr. Zool.* **64**, 33–43 (2018).

18. Luo, Y. *et al.* Metabolism and thermoregulation between Mrs Hume's Pheasant (S*yrmaticus humiae*) and Elliot's Pheasant (*S. ellioti*). *Dongwuxue. Yanjiu.* **32**, 396–402 (2011).

19. Yarbrough, C. G. The influence of distribution and ecology on the thermoregulation of small birds. *Comp. Biochem. Physiol. -- Part A Physiol.* **39**, 235–266 (1971).

20. Bennettand, P. M. & Harvey, P. H. Active and resting metabolism in birds: allometry, phylogeny and ecology. *J. Zool.* **213**, 327–344 (1987).

21. Weathers, W. W. & Caccamise, D. F. Temperature regulation and water requirements of the monk parakeet, Myiopsitta monachus. *Oecologia* **18**, 329–342 (1975).

22. Weathers, W. W. & Caccamise, D. F. Seasonal acclimatization to temperature in monk parakeets. *Oecologia* **35**, 173–183 (1978).

23. Lovegrove, B. G., Perrin, M. R. & Brown, M. The allometry of parrot BMR: Seasonal data for the Greater Vasa Parrot, *Coracopsis vasa*, from Madagascar. *J. Comp. Physiol. B Biochem. Syst. Environ. Physiol.* **181**, 1075–1087 (2011).

24. Thabethe, V., Thompson, L. J., Hart, L. A., Brown, M. & Downs, C. T. Seasonal effects on the thermoregulation of invasive rose-ringed parakeets (*Psittacula krameri*). *J. Therm. Biol.* **38**, 553–559 (2013).

Supplementary Figures

Supplementary Figure 1a,b: niche dynamic (expansion, unfilling, stability) indices for invasive birds introduced to Europe.

Supplementary Figure 2: predicted invasive bird species richness across Europe according to correlative versus ecophysiological model forecasts.

Supplementary Figure 3: invasion risk forecasts for invasive birds introduced to Europe as derived from ecophysiological (NicheMapper) models.

Supplementary Fig 4: relationship between model sensitivity and specificity and the amount of niche expansion detected.

Supplementary Fig 5: relative biophysical trait importance derived from sensitivity analyses on mechanistic niche models for birds invading Europe

Supplementary Figure 6: predictive accuracy of invasion risk forecasts obtained from mechanistic models in relation to species' biophysical traits and introduction history.

Supplementary Figure 7: impact of changing values of different input parameters (i.e. sensitivity analyses) on the geographical area identified as suitable by mechanistic models.

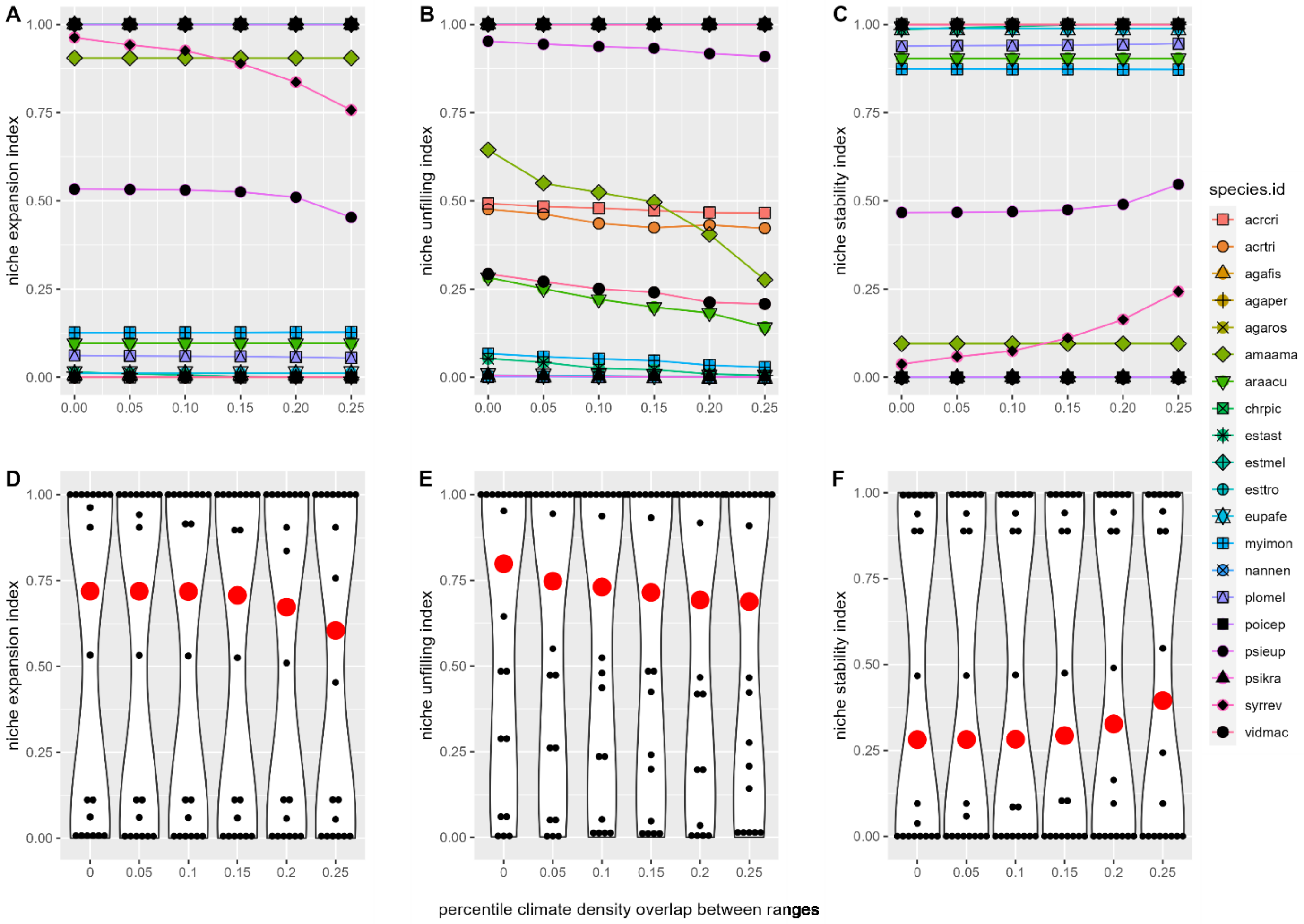

Supplementary Fig 1a. Niche dynamics (A, D: niche expansion; B, E: niche unfilling; C, F: niche stability) for 20 bird species introduced to Europe obtained using the whole of Europe as invasive range 'background' area. The x-axes show the percentile of the available environmental conditions in each range used to calculate the niche dynamic indexes (y-axes), varying from the 75th to the 100th percentile (=including all marginal climates). A-C represent the index value for each species, D-F represent summary statistics expressed through violin plots (each black dot is a species, red dot indicates the median value).

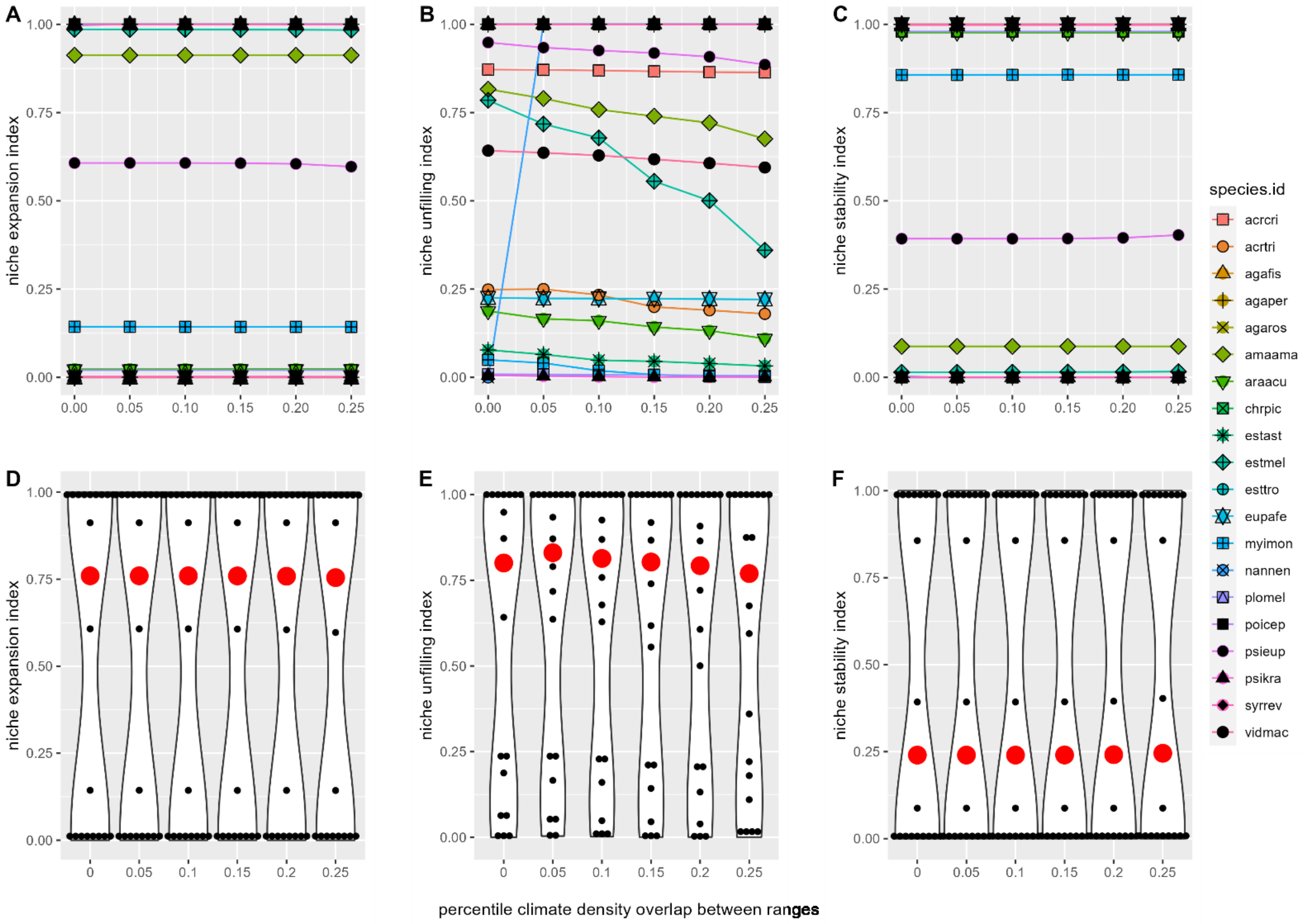

Supplementary Fig 1b. Niche dynamics (A, D: niche expansion; B, E: niche unfilling; C, F: niche stability) for 20 bird species introduced to Europe obtained using an invasive range background defined based on invasion history (residence time and invasion speed). The x-axes show the percentile of the available environmental conditions in each range used to calculate the niche dynamic indexes (y-axes), varying from the 75th to the 100th percentile (=including all marginal climates). A-C represent the index value for each species, D-F represent summary statistics expressed through violin plots (each black dot is a species, red dot indicates the median value).

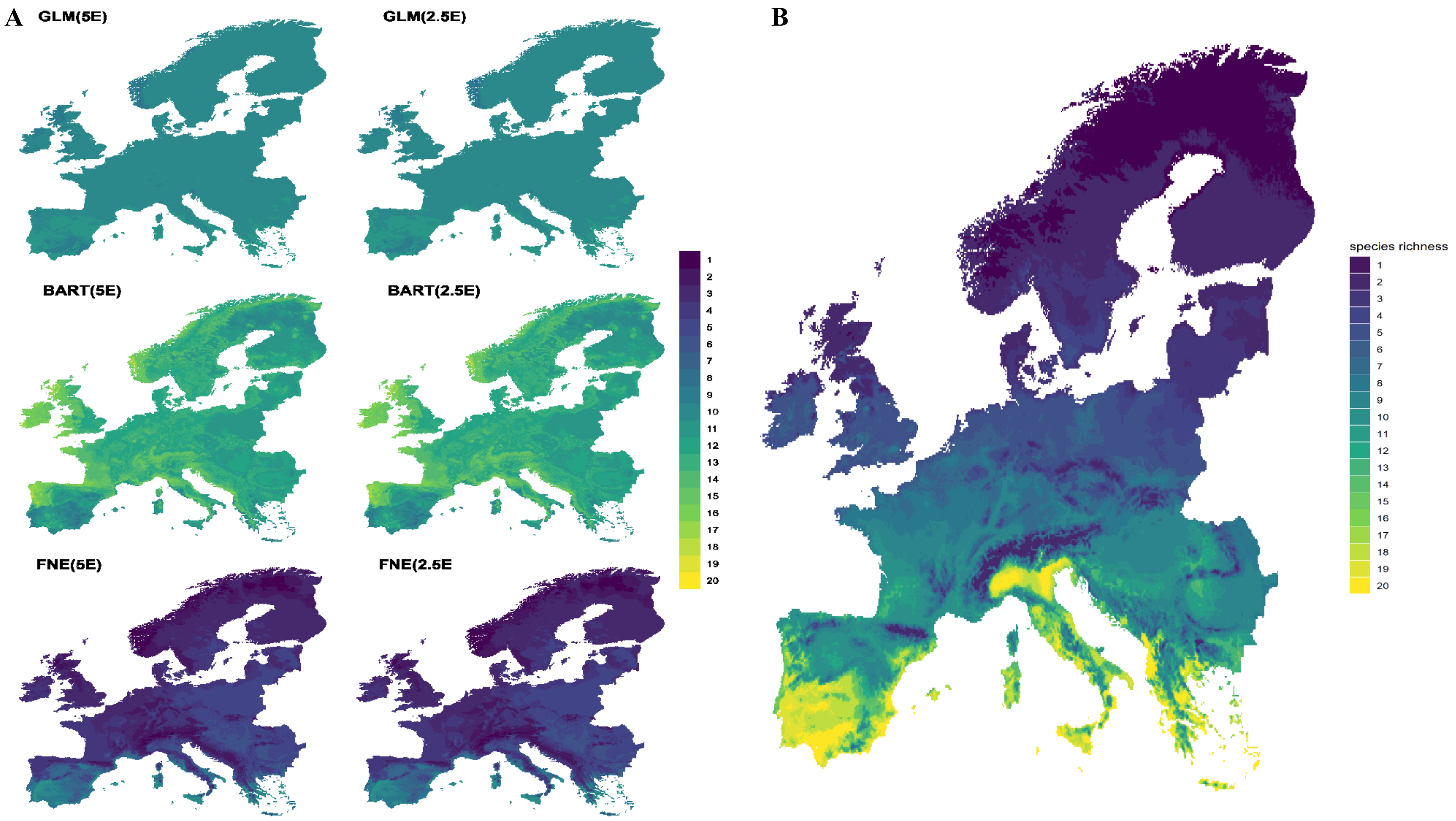

Supplementary Fig 2A,B. Predicting invasive bird species richness across Europe based on the 20 introduced bird species considered here. **A**: Richness forecasts according to correlative model forecasts (GLM: Generalized Linear Model, BART: Bayesian Additive regression trees, FNE: Fundamental Niche Ellipses; 5E: native-range omission threshold set to 5%, 2.5E: native range omission threshold set to 2.5%). **B**: Richness forecast according to the species-level NicheMapper ecophysiological model. Predicted invasive distributions for each species and SDM method separately can be found at https://github.com/LauraJim/Modeling_bird_invasions.

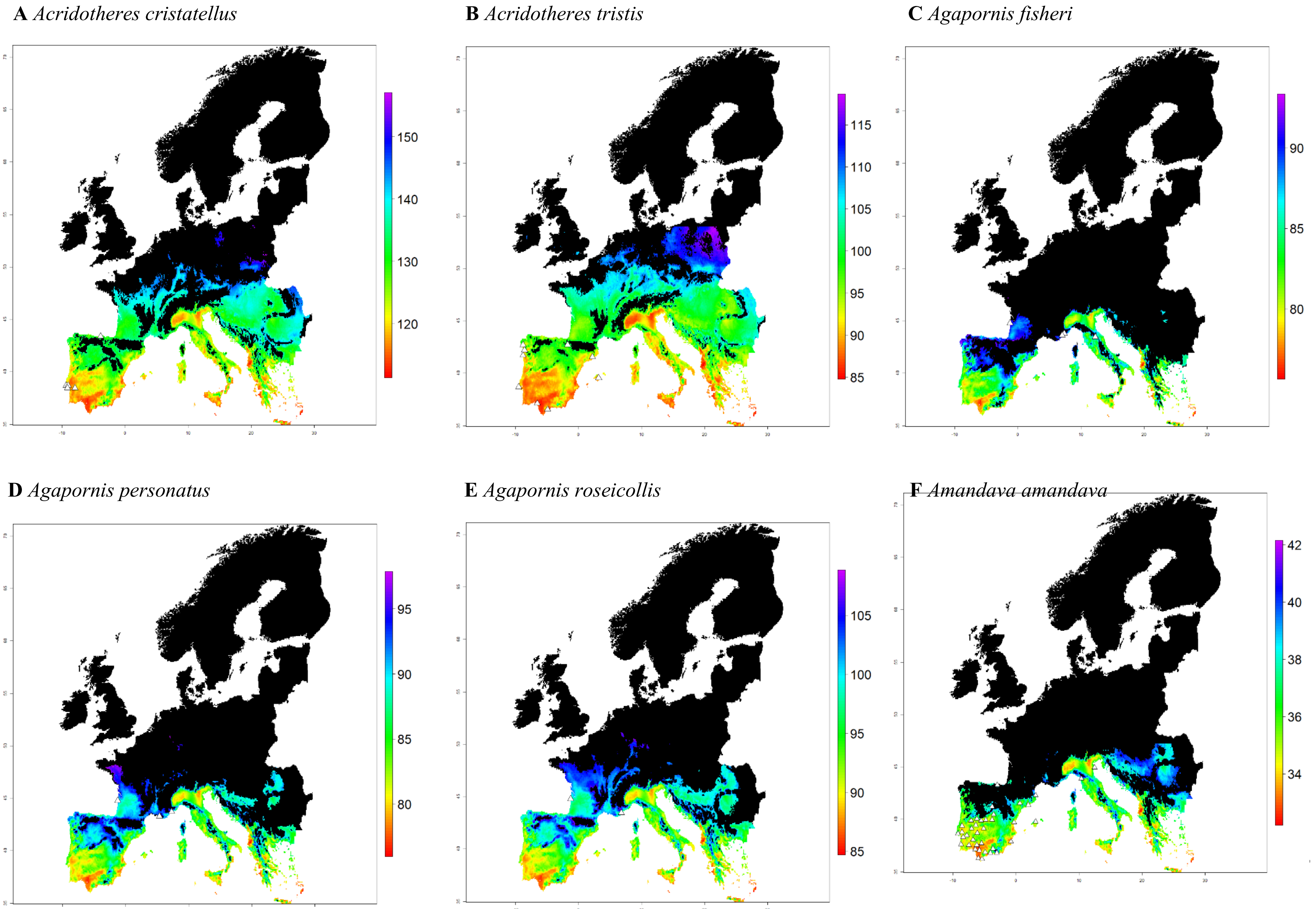

A *Acridotheres cristatellus*
150
140
130
120
B *Acridotheres tristis*
115
110
105
100
95
90
85
C *Agapornis fisheri*
90
85
80
D *Agapornis personatus*
95
90
85
80
E *Agapornis roseicollis*
105
100
95
90
85
F *Amandava amandava*
42
40
38
36
34

G *Thectocercus (Aratinga) acuticaudatus*

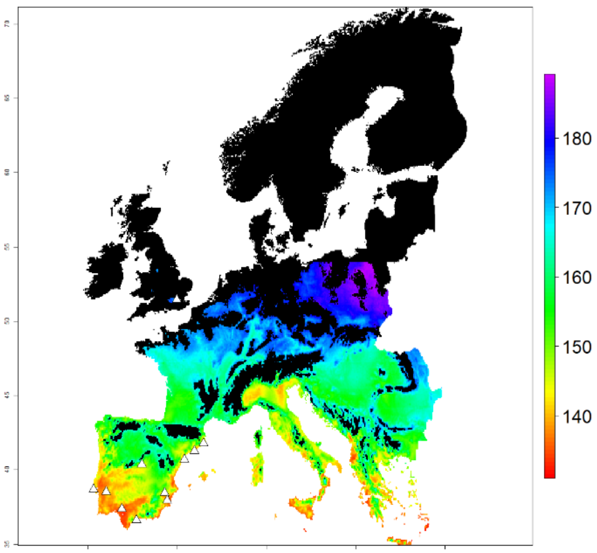

H *Chrysolophus pictus*

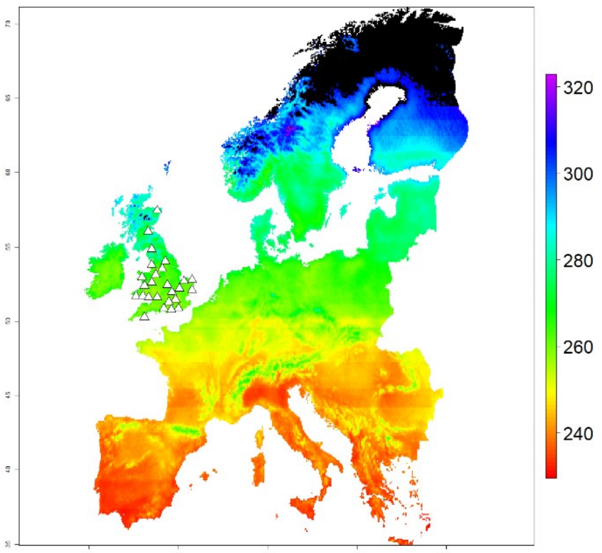

I *Estrilda astrild*

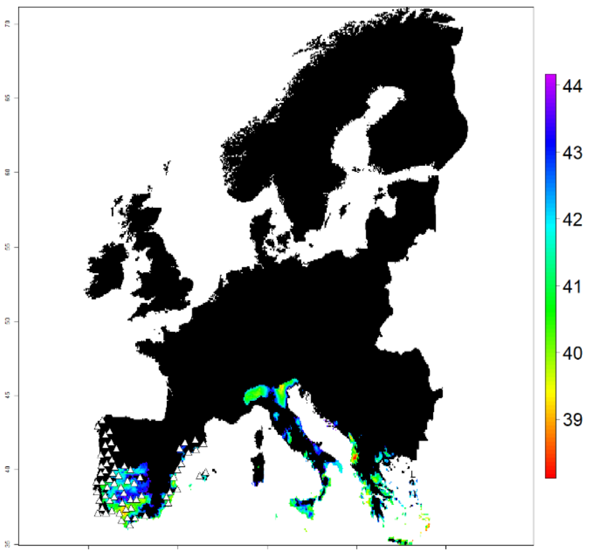

J *Estrilda melpoda*

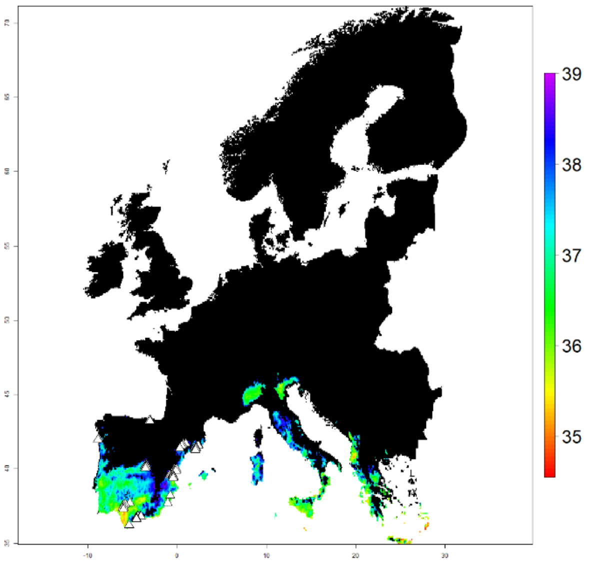

K *Estrilda troglodytes*

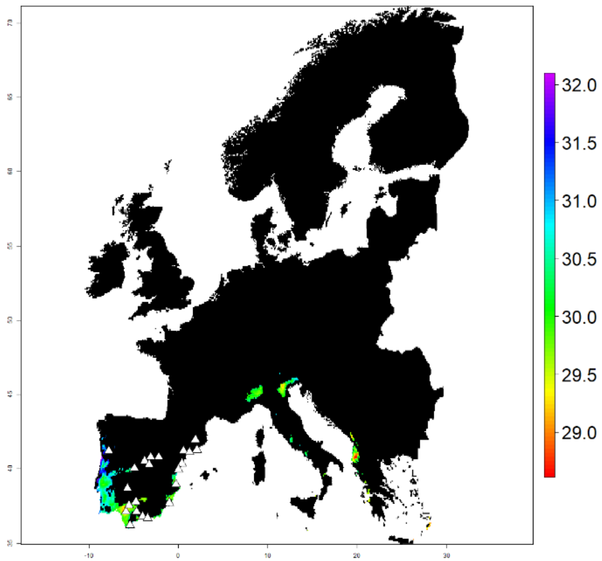

L *Euplectes afer*

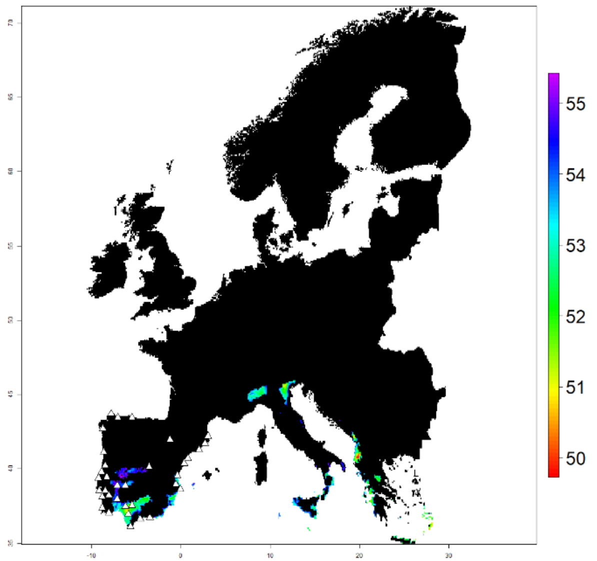

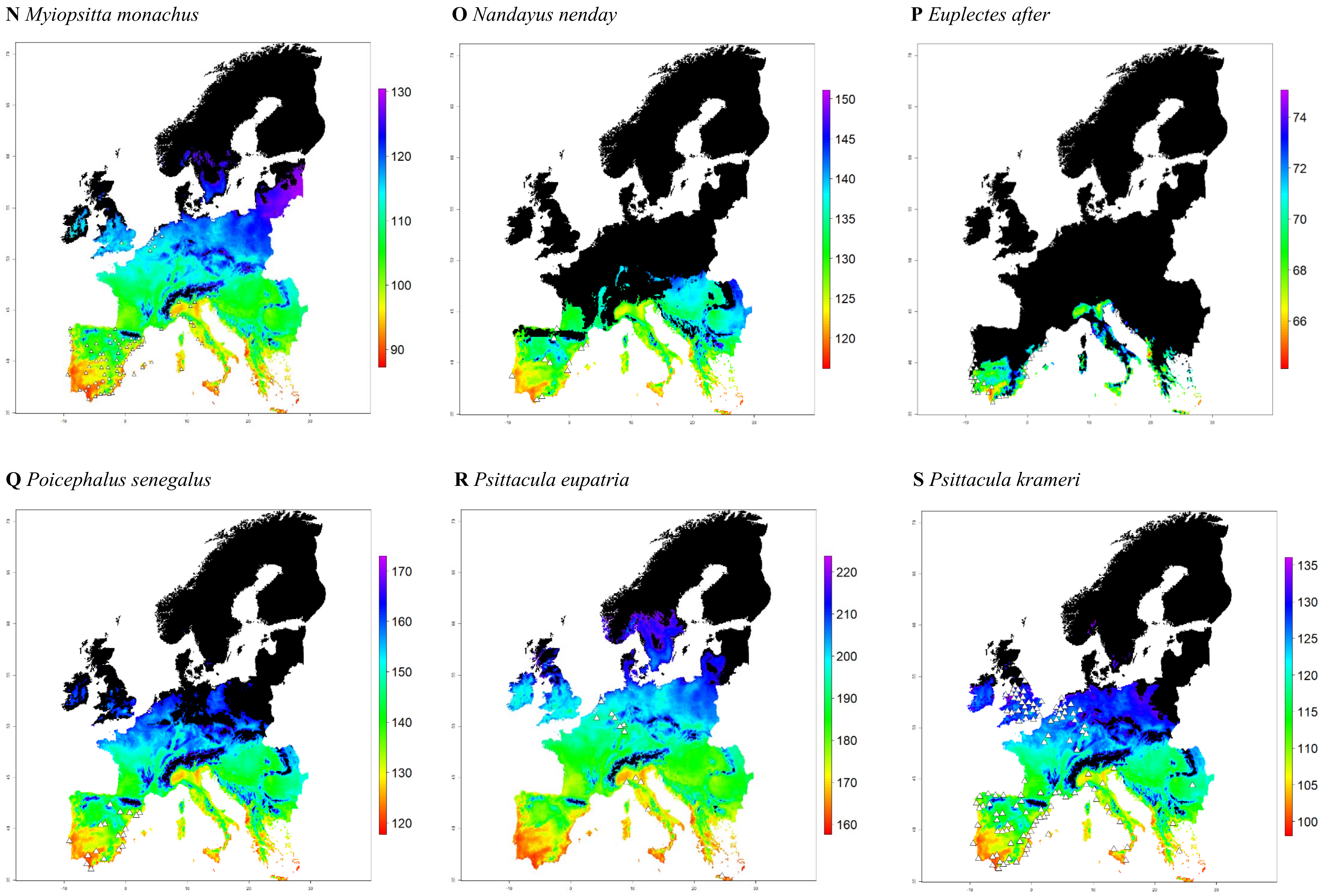

N *Myiopsitta monachus*
130
120
110
100
90
O *Nandayus nenday*
150
145
140
135
130
125
120
P *Euplectes after*
74
72
70
68
66
Q *Poicephalus senegalus*
170
160
150
140
130
120
R *Psittacula eupatria*
220
210
200
190
180
170
160
S *Psittacula krameri*
135
130
125
120
115
110
105
100

T *Syrmaticus reveesi*

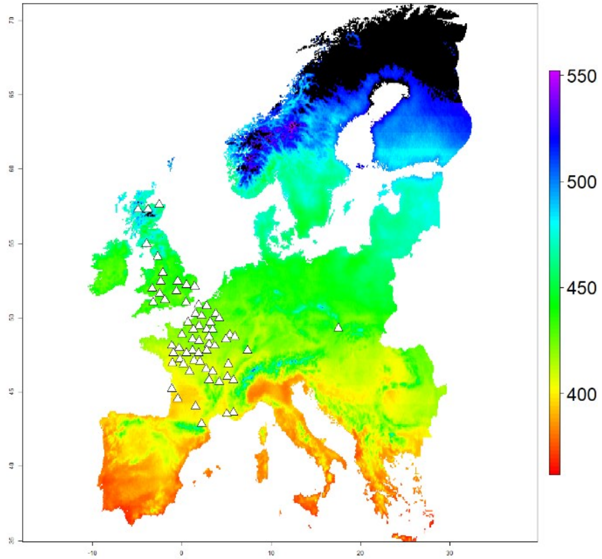

U *Vidua macroura*

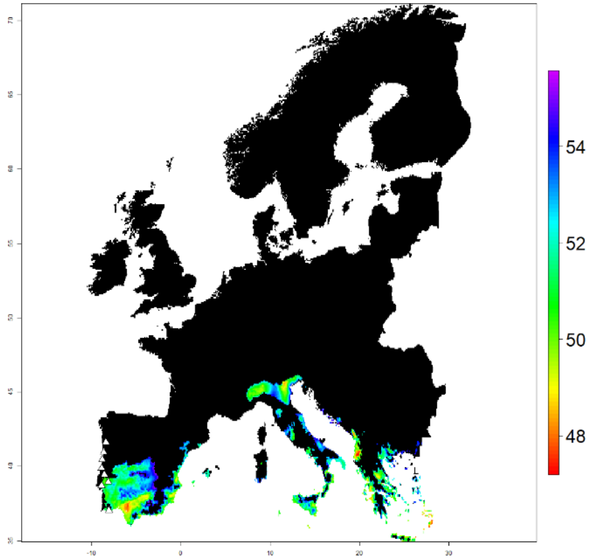

Supplementary Fig 3A-U. Forecasts of invasion risk across Europe for each of the 20 introduced bird species considered here. Black areas indicate parts of Europe that are forecasted to be unsuitable for introduced bird species, as the amount of energy required for individuals to remain in homeothermy is considered outside of species' metabolic reach (i.e. more than 4.6 times a species' basal metabolic rate). Colored areas are predicted to be at risk of invasion, whereby warmer colors indicate lower thermoregulatory costs. Map units are megaJoule per year. Corresponding maps for the correlative models applied here can be found at https://github.com/LauraJim/Modeling_bird_invasions.

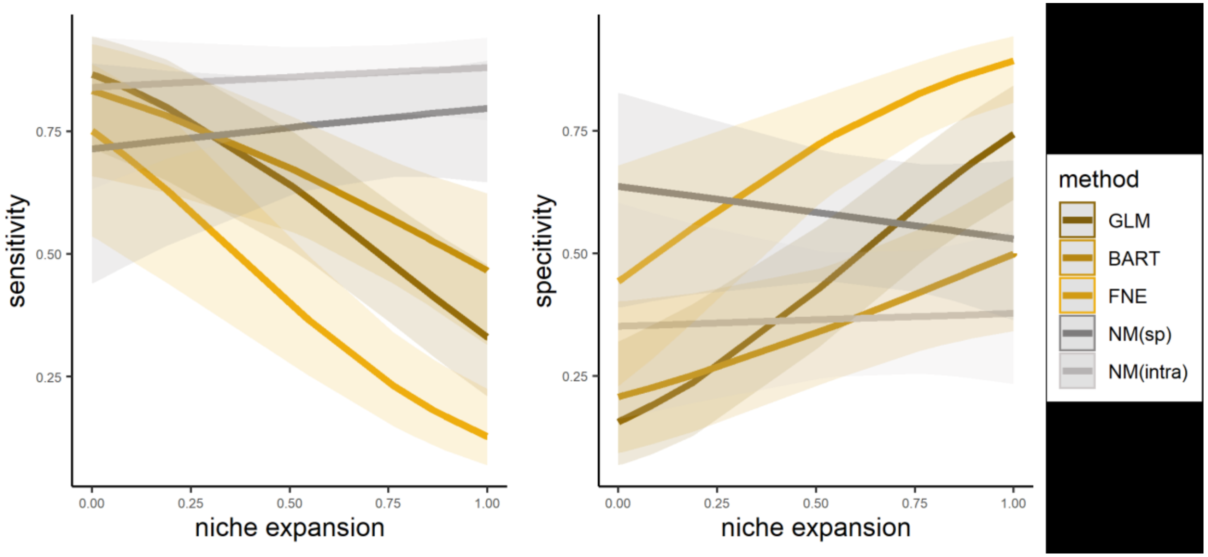

Supplementary Fig 4. Relationship between the amount of niche expansion into novel climates across the invaded range and the capacity of models to correctly identify invasive occurrences (sensitivity) and locations currently free from invasive occurrences (‘pseudoabsences’, specificity). Mechanistic models (‘NM’) are indicated in grey colors, correlative models (GLM, BART, FNE) in yellow to brown. Solid lines represent the mean estimate, shading indicates the 95% confidence interval of significant relationships.

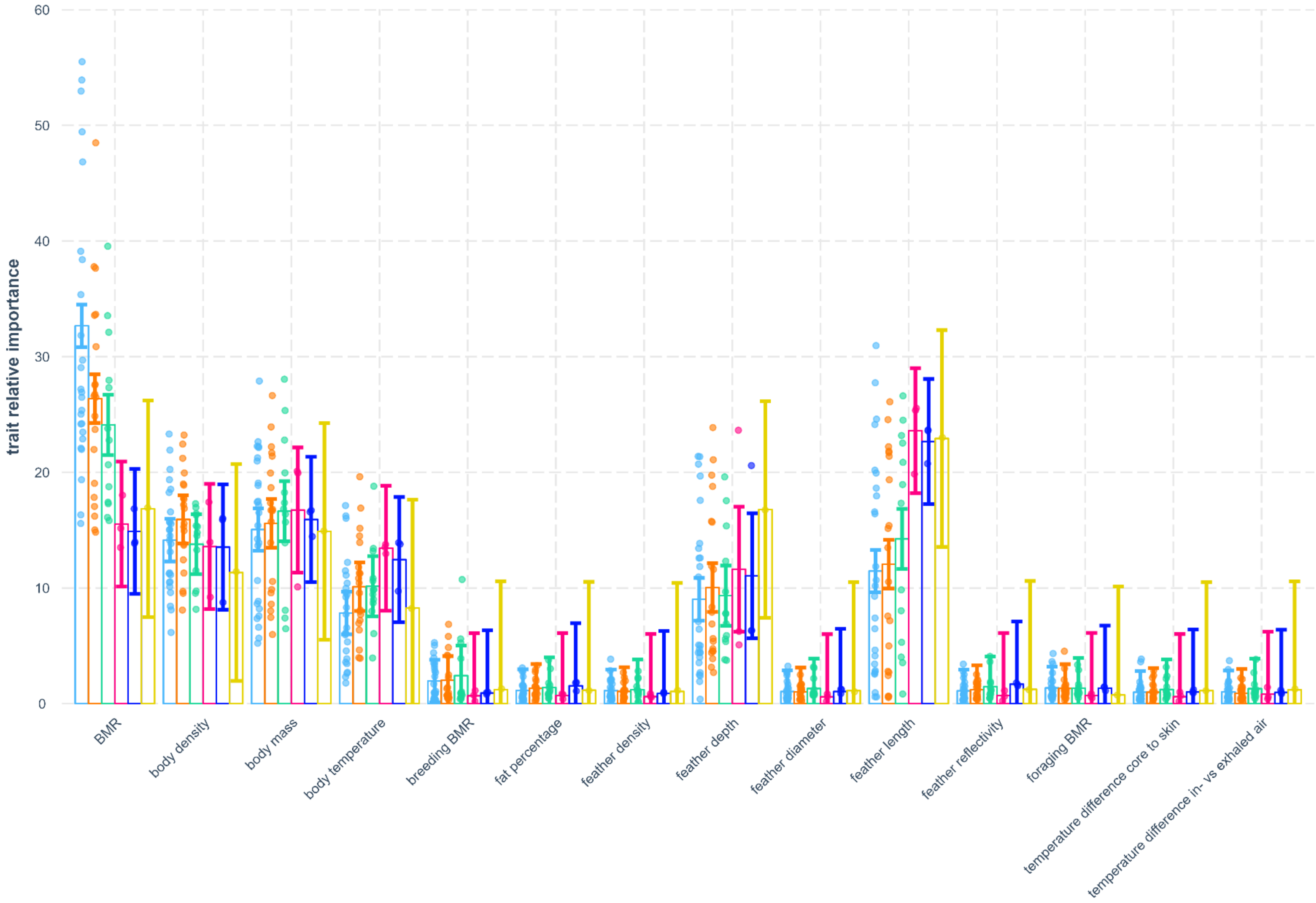

Supplementary Fig 5. Relative biophysical trait importance derived from sensitivity analyses on mechanistic niche models for birds invading Europe ‘(n= 20 introduced bird species). Sensitivity model iterations were run until gain in narrowing trait values was lower than 5% for any of the fourteen traits considered. Colours indicate model iterations. Data are presented as mean values ± SD.

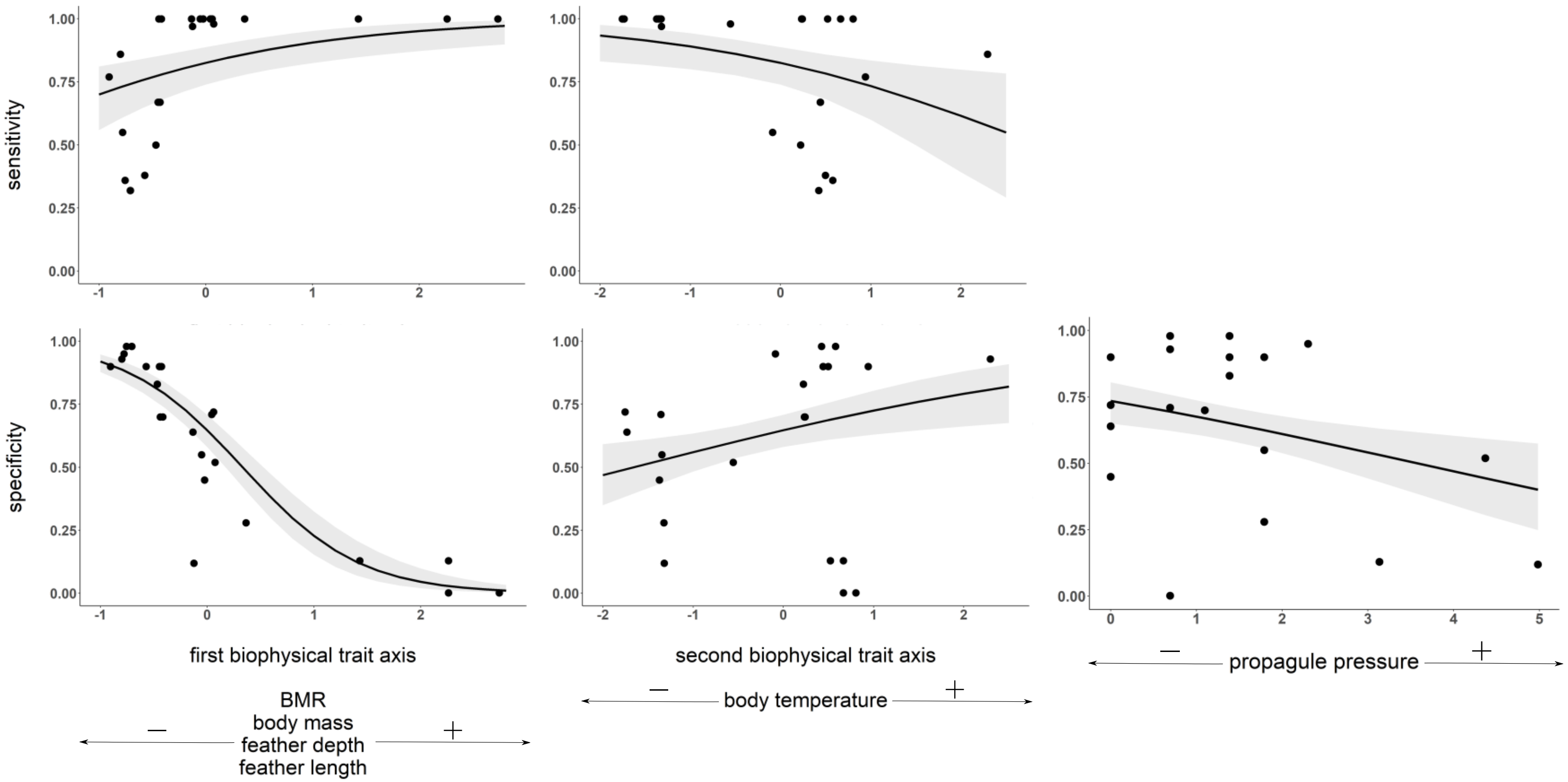

Supplementary Fig 6. Predictive accuracy (sensitivity and specificity) of invasion risk forecasts obtained from mechanistic models in relation to species' biophysical traits and introduction history (significant relationships only). Each dot represents a bird species, and data are slightly jittered along the x-axis for visibility. Solid black lines represent the mean estimate, grey shading indicates the 95% confidence interval of significant relationships.

Supplementary Figure 7: impact of changing values of different input parameters (i.e. sensitivity analyses) on the geographical area identified as suitable by mechanistic models. Data are scaled to a mean of 0 and a standard deviation of 1. Solid black lines represent the mean estimate, grey shading indicates the 95% confidence interval of significant relationships.

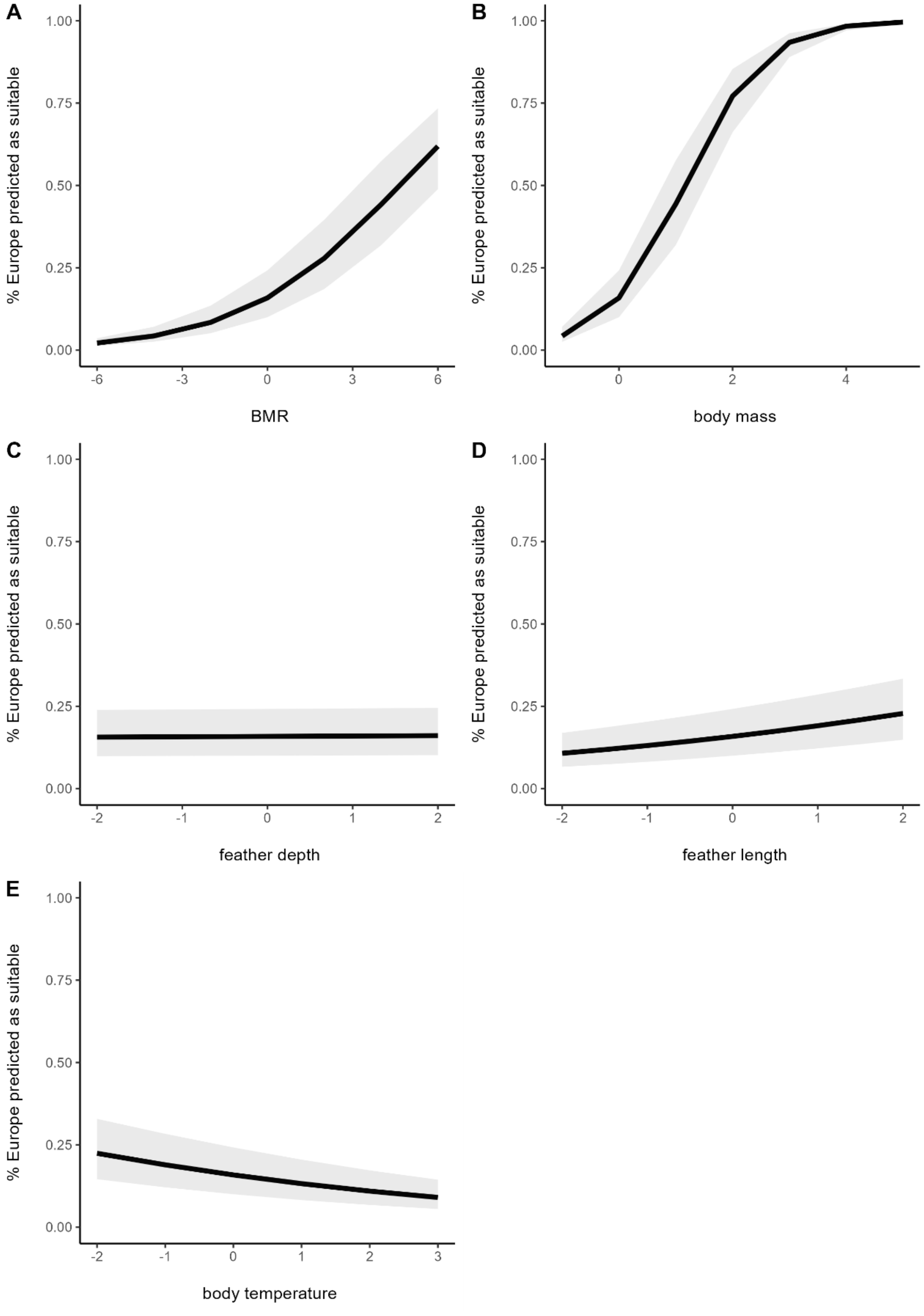

Supplement: Supplementary file 1 — Supplementary Information [file 41467_2023_38329_MOESM1_ESM.pdf]
