# Supplementary material for: Mechanistic models project bird invasions with accuracy
Source: Nat Commun. 2023 May 2;14:2520. doi: 10.1038/s41467-023-38329-4 (PMC10154326; doi:10.1038/s41467-023-38329-4)
Supplement: Supplementary file 8 — Reporting Summary [file 41467_2023_38329_MOESM8_ESM.pdf]

nature portfolio

Corresponding author(s): diederik.strubbe@ugent.be

Last updated by author(s): Apr 25, 2023

Reporting Summary

Nature Portfolio wishes to improve the reproducibility of the work that we publish. This form provides structure for consistency and transparency in reporting. For further information on Nature Portfolio policies, see our Editorial Policies and the Editorial Policy Checklist.

Statistics

For all statistical analyses, confirm that the following items are present in the figure legend, table legend, main text, or Methods section.

n/a	Confirmed	
☐	☒	The exact sample size (*n*) for each experimental group/condition, given as a discrete number and unit of measurement
☐	☒	A statement on whether measurements were taken from distinct samples or whether the same sample was measured repeatedly
☐	☒	The statistical test(s) used AND whether they are one- or two-sided<br>*Only common tests should be described solely by name; describe more complex techniques in the Methods section.*
☐	☒	A description of all covariates tested
☐	☒	A description of any assumptions or corrections, such as tests of normality and adjustment for multiple comparisons
☐	☒	A full description of the statistical parameters including central tendency (e.g. means) or other basic estimates (e.g. regression coefficient) AND variation (e.g. standard deviation) or associated estimates of uncertainty (e.g. confidence intervals)
☐	☒	For null hypothesis testing, the test statistic (e.g. *F*, *t*, *r*) with confidence intervals, effect sizes, degrees of freedom and *P* value noted<br>*Give P values as exact values whenever suitable.*
☒	☐	For Bayesian analysis, information on the choice of priors and Markov chain Monte Carlo settings
☐	☒	For hierarchical and complex designs, identification of the appropriate level for tests and full reporting of outcomes
☐	☒	Estimates of effect sizes (e.g. Cohen's *d*, Pearson's *r*), indicating how they were calculated

Our web collection on statistics for biologists contains articles on many of the points above.

Software and code

Policy information about availability of computer code

Data collection	R version 4.2.1 (2022-06-23 ucrt) -- "Funny-Looking Kid"
Data analysis	R version 4.2.1 (2022-06-23 ucrt) -- "Funny-Looking Kid". The following packages were used for data analysis: embarcadero (version 1.2.0.1003), stats (version 4.2.1), modEva (version 3.5) , blockCV (version 2.1.4), kuenm (version 1.1.9), enmSdm (version 0.9.3), CoordinateCleaner (version 2.0.20), , rgeos (version 0.5.9), sampbias (version 1.04), ENMeval (version 2.0.4), glmmTMB (version 1.1.4), betareg (version 3.2.4)<br>NicheMapper endotherm module Endo2017.exe<br>NicheMapper microclimate module Micr2010a.exe<br>All codes are available at https://github.com/LauraJim/Modeling_bird_invasions

For manuscripts utilizing custom algorithms or software that are central to the research but not yet described in published literature, software must be made available to editors and reviewers. We strongly encourage code deposition in a community repository (e.g. GitHub). See the Nature Portfolio guidelines for submitting code & software for further information.

Data

Policy information about availability of data

All manuscripts must include a data availability statement. This statement should provide the following information, where applicable:

- Accession codes, unique identifiers, or web links for publicly available datasets
- A description of any restrictions on data availability
- For clinical datasets or third party data, please ensure that the statement adheres to our policy

The data gathered and processed, and resulting model outcomes (GIS maps and associated model evaluation statistics), are openly available via the Zenodo database under accession code 10.5281/zenodo.7733648.

Research involving human participants, their data, or biological material

Policy information about studies with human participants or human data. See also policy information about sex, gender (identity/presentation), and sexual orientation and race, ethnicity and racism.

Reporting on sex and gender	NA
Reporting on race, ethnicity, or other socially relevant groupings	NA
Population characteristics	NA
Recruitment	NA
Ethics oversight	NA

Note that full information on the approval of the study protocol must also be provided in the manuscript.

Field-specific reporting

Please select the one below that is the best fit for your research. If you are not sure, read the appropriate sections before making your selection.

☐ Life sciences ☐ Behavioural & social sciences ☒ Ecological, evolutionary & environmental sciences

For a reference copy of the document with all sections, see nature.com/documents/nr-reporting-summary-flat.pdf

Ecological, evolutionary & environmental sciences study design

All studies must disclose on these points even when the disclosure is negative.

Study description	The study aims to test the accurracy of predictions of invasion risk for invasive birds introduced to Europe derived from mechanistic versus correlative species distribution models. Mechanistic models are created using the NicheMapper platform, a biophysical model that was paramterized based on data gleaned from the literature and from (morphological) traits measured on museum specimens. Correlative models were run in R using a set of SDM algorithms, and were parametrized based on species occurrence data downloaded from GBIF.
Research sample	The study focussed on 20 birds species introduced to Europe: Acridotheres cristatellus, Acridotheres tristis, Agapornis fischeri, Agapornis personatus, Agapornis roseicollis, Amandava amandava, Aratinga acuticaudata, Chrysolophus pictus, Estrilda astrild, Estrilda melpoda, Estrilda troglodytes, Euplectes afer, Myiopsitta monachus, Nandayus nenday, Ploceus melanocephalus, Poicephalus senegalus, Psittacula eupatria, Psittacula krameri, Syrmaticus reveesi, Vidua macroura
Sampling strategy	Occurrence data were downloaded from GBIF (see Supplementary Table 5 for download DOIs). From the set of known non-native bird species in Europe, we selected terrestrial bird species that occurred in at least five different locations (the minimum sample size for niche dynamic analyses), that did not have any part of their native range in Europe, and for which museum specimens were present in the Royal Belgian Institute of Natural Sciences (RBINS, Brussels, Belgium), resulting in 20 species for this study
Data collection	Correlative model data were downloaded via GBIF (see above), the literature review to gather data for parametrizing NicheMapper was carried out by Diederik Strubbe
Timing and spatial scale	Climate data represent 1970-2000, habitat data: 2015-2019 and species occurrence data from 1960 to 2019. Spatial scale is (near) global (native distribution ranges of 20 birds species plus Europe). The focus is on prediction invasion risk for Europe.
Data exclusions	The only data that were omitted from this study are downloaded GBIF occurrence data points that were marked as potentially problematic by the CoordinateCleaner package. This package removes occurrences with potentially problematic geographical coordinates, such as country centroids, equal longitude-latitude observations, GBIF headquarters, biodiversity institutions and zero coordinates.

Reproducibility	All input data and codes are available from https://github.com/LauraJim/Modeling_bird_invasions
Randomization	Possible non-independence of data was accounted for (1) by rarefying downloaded GBIF species occurrence data with a distance of 50 km, (2) by including species and model algorithm as random factors in regression models.
Blinding	Most data were collected before this study started, i.e. were collected blindly regarding hypotheses tested in the present study.

Did the study involve field work? ☐ Yes ☒ No

Reporting for specific materials, systems and methods

We require information from authors about some types of materials, experimental systems and methods used in many studies. Here, indicate whether each material, system or method listed is relevant to your study. If you are not sure if a list item applies to your research, read the appropriate section before selecting a response.

Materials & experimental systems

n/a	Involved in the study
☒	☐ Antibodies
☒	☐ Eukaryotic cell lines
☒	☐ Palaeontology and archaeology
☒	☐ Animals and other organisms
☒	☐ Clinical data
☒	☐ Dual use research of concern
☒	☐ Plants

Methods

n/a	Involved in the study
☒	☐ ChIP-seq
☒	☐ Flow cytometry
☒	☐ MRI-based neuroimaging
