# Supplementary material for: Mechanistic models project bird invasions with accuracy
Source: Nat Commun. 2023 May 2;14:2520. doi: 10.1038/s41467-023-38329-4 (PMC10154326; doi:10.1038/s41467-023-38329-4)
Supplement: Supplementary file 2 — Peer Review File [file 41467_2023_38329_MOESM2_ESM.pdf]

nature portfolio

Peer Review File

Mechanistic models project bird invasions with accuracy

Open Access This file is licensed under a Creative Commons Attribution 4.0 International License, which permits use, sharing, adaptation, distribution and reproduction in any medium or format, as long as you give appropriate credit to the original author(s) and the source, provide a link to the Creative Commons license, and indicate if changes were made. In the cases where the authors are anonymous, such as is the case for the reports of anonymous peer reviewers, author attribution should be to 'Anonymous Referee' followed by a clear attribution to the source work. The images or other third party material in this file are included in the article's Creative Commons license, unless indicated otherwise in a credit line to the material. If material is not included in the article's Creative Commons license and your intended use is not permitted by statutory regulation or exceeds the permitted use, you will need to obtain permission directly from the copyright holder. To view a copy of this license, visit http://creativecommons.org/licenses/by/4.0/.

Reviewer comments, first round –

Reviewer #1 (Remarks to the Author):

In this study, the authors applied both ecophysiological mechanistic models and correlative models to predict the distribution of alien bird species in Europe and to assess their predictive performance in the invasive range for those species. They found the clear niche shift for most alien birds and the poor performance of correlative models in the invasive range. In contrast, mechanistic models can accurately predict the distribution of alien species. Although I am a freshwater fish ecologist, I also collaborated my colleagues to understand the role of different factors on the impacts of alien bird invasions. To my knowledge, bird should be one of the taxa that are suitable for mechanistic models because of the sufficient information. I like this study, because it paved the way of applying mechanistic models to predict the invasion risks of alien species, which is important for future studies and management. I listed my comments, which could be useful to improve this study before it is accepted:

The current Introduction addressed the meaning of studying biological invasions and predicting the invasion risks. Given this study focuses on applying mechanistic models to predict the distribution of invasive species, it would be good to provide some background information about the current process of related researches, so that the readers could have a better understanding of the application of mechanisms models for invasive species.

Given the first paragraph of the Results section is about realized niche shifts, maybe the analyses of niche shift in the Materials and Methods section (which is currently at the end) can be moved forward to some extent? In addition, it may be good to have a figure summarizing the results of niche shift across all species.

Given they have the results of both niche shift and the performance of mechanistic and correlative models, it would be interesting to see the relationship between the magnitude of niche shift and model performance across species. The results would be informative to identify whether models can accurately predict the distribution of alien species in the invasive range based on the results of niche shift.

Another interesting result should be the overall geographical patterns of invasion risks for all 20 species based on the predictions of mechanistic and correlative models, respectively. These results could provide insights on the difference in predicted invasions risks using models with good and poor performance.

I think one part that can be added to the current Discussion section could be how the findings of this study can be used to guide the prediction of invasions risks for alien species. For example, whether we can have more reliable predictions for a certain group of species given the clear influence of biophysical traits on the performance of mechanistic models?

For the figures, it would be good to have the first sentence in the caption to briefly describe of the figure. Currently, the first sentence of Figure 1 shows the meaning of successfully predicting invasion risks, and the first sentence of Figure 2 shows the main results (I rarely see that the caption shows the results).

In the Materials and Methods section, I would like to see how the 20 bird species were selected, because this manuscript didn't give the description of these bird species. Based on the texts in other sections, I guess the species are those introduced to Europe? It is also important to know how many presence data points are in the native and invasive range for each species.

For the paragraph started from Line 405 with "The endotherm model", there is too much detailed information about each trait. Although I know such information is important, I don't think they are as important as other text, such as the modeling setting. There are 959 words in one paragraph, I suggest some texts can be moved to the Supplement Materials if the authors want to keep those

words.

I also listed some minor concerns:

Linke 115-118, this sentence is better move to the Discussion section, which is based on the findings in this study.

Line 137, they need to mention that “65 and 45%” are the results of intra-specific levels and species-level respectively.

Line 548, I think “Intraspecific-level models predictive accuracy” should be changed to “The predictive accuracy of intraspecific-level models”?

Chunlong Liu

Reviewer #2 (Remarks to the Author):

I read this manuscript with considerable anticipation, particularly given the list of authors--I genuinely enjoy reviewing papers when I am able to learn from them at the same time. That said, however, I was disappointed in the comparison that was presented--the mechanistic models are presented strongly, but the correlative models developed are weak, such that it is easy to then say that one is better than the other. I think that a more appropriate correlative analysis would reveal that both approaches have merit, and that an ideal analysis will include both approaches.

The following are some key points:

1. "Currently, invasion risk forecasts are usually based on extrapolating environmental tolerances derived from species’ current native ranges to new areas. However, such correlative niche modeling often fails to correctly predict invasive occurrences, leading to risk-ignorant decisions and miscalculation of expected impacts and costs". This paper is not set up as an objective comparison of two parallel methodologies. Rather, it is clear from the outset that the authors have/had made up their minds before the study was initiated, and set out to prove their point. A more appropriate introduction might set out the positive achievements of both methods, as well as the assumptions and drawbacks of each.

2. Line 36 -- Focusing exclusively on thermal niches represents a clear and significant drawback of the mechanistic approach presented. The authors might well comment on the frequency with which non-thermal dimensions are most important (limiting) across birds. My understanding is that non-thermal dimensions are indeed frequently important, and yet they are entirely ignored in the approaches presented.

3. "While such correlative species distribution models (SDM) perform well in characterizing the natural distributions of species within their current range15, multiple statistical and eco-evolutionary issues arise when models are transferred to new areas or time periods, introducing uncertainty into model forecasts and decreasing reliability of forecasts..." Again, the emphasis appears to be on "shooting down" the correlative approaches, rather than on presenting an objective comparison of the two approaches. For instance, although the authors are abundant in citing studies that document drawbacks of the correlative approaches, they have not cited a review of the two methods that pointed out some serious concerns (re overparameterization) of the mechanistic approaches--Peterson, A. T., M. Papeş, and J. Soberón. 2016. Mechanistic and correlative models of ecological niches. European Journal of Ecology 1:28-38. I will apologize for pointing the authors toward one of my own publications, but it does genuinely point to problems with the mechanistic approaches.

4. "Also, presumed relationships between correlative estimates of climatic suitability and the life-history traits and mechanisms that determine species persistence have been found to be

questionable at best" Again, this seems to be a gratuitous potshot at the correlative methodology: the same sort of generalization could be aimed at the simplifications involved in the mechanistic modeling process, but it is not.

5. "Using species' realized niches for forecasting invasion risks is likely to result in..." This is incorrect: correlative modeling approaches *attempt to* approximate the fundamental, not the realized, niche. However, the approaches used by the authors of this manuscript are clearly going to replicate/estimate the realized niche, and not the fundamental. See comments below on this point.

6. "Correlative models based on native range occurrences accurately described species' native distributions (Supplementary Material 1), but when extrapolating these models to Europe to obtain invasion risk forecasts, they incorrectly delineated the whole continent as unsuitable for 18 out of the 20 species studied here (Fig. 2)." Here, much more more information is needed about how extrapolations were carried out... E.g., was a MOP-style assessment made of the extrapolation risk? how were model predictions extended to non-analog conditions? etc.

7. "Possible reasons for this mediocre performance are many, but most prominently include the inherent assumption of realized niche conservatism..." My 2011 review of niche conservatism is cited here, but incorrectly. Correlative model transfers indeed depend on an assumption of niche conservatism, but not of the realized niche! Rather, given the definition of the realized niche (= fundamental niche reduced by accessible conditions to the existing niche, which is then further reduced by biotic interactions to the realized niche), any assumption of realized niche conservatism would be folly, and none of my work has made any such assertion.

8. "Ecophysiological models only use occurrence data from the invaded range to evaluate forecasts of invasion risk, while correlative SDMs rely on native-range occurrences also for model calibration." This point is particularly important--there is no real comparative evaluation of performance of two different approaches here! Rather, the comparison is between apples and squid, in my opinion. That is, how can one compare the performance of two approaches when the data on which the two are based are entirely different? Indeed, it appears that different environmental datasets were also employed in the two approaches... so we are reduced to comparing digital images of apples to poetry about squid.

9. "Here, we first ran such a species-level model, which was then subjected to a sensitivity analysis uncovering the most influential variables. These variables were then considered in an intraspecific-level model accounting for trait variation." I consider such a sensitivity analysis to be crucial, yet no information was presented as to what sensitivity analyses were run: I would assume that parameter estimates were varied by small increments around the central estimate, yet varying by 1% or 5% or what? And was each parameter varied independently or were they varied in tandem? And with what precision was each original parameter estimate known? Many appear to be very general assumptions. In the Peterson et al. paper in European Journal of Ecology paper mentioned above, we performed such an analysis using the NicheMapper software, and found that the results were extremely sensitive to specific parameter values.

10. "Species distribution modeling was done using the 'sdm' the ensemble modelling framework140. We applied eight different modelling algorithms: generalized linear models (GLM), generalized additive models (GAM), random forest (RF), maximum entropy (MaxEnt), maximum likelihood (MaxLike), flexible discriminant analysis (FDA), multiple adaptive regression splines (MARS) and generalized boosted models (GBM)." A long series of methodological decisions will plague any attempt to argue that the correlative models in this study are able to reconstruct the fundamental niche (which is the only niche model that can or should be used for model transfers). The choice of algorithms includes many approaches that will estimate complex response shapes, when fundamental niches are likely simple and convex (see https://www.biorxiv.org/content/biorxiv/early/2021/01/27/2021.01.25.428165.full.pdf). No information is presented about how calibration areas were selected, even though that decision is crucial to model success (https://www.sciencedirect.com/science/article/pii/S0304380011000780?casa_token=dAHhLbHyE88AAAAA:oR-c5iPJSiE4e4Au0y7wZCuvPZlaqe-Jy4aSsMJ4SJdVOEPiLnnqlBDz4iga804FtKPAtxft7Q)

and to appropriate consideration of uncertainty regarding niche limits (https://onlinelibrary.wiley.com/doi/pdf/10.1002/ece3.6359). TSS approaches are used for model evaluation, even though they require absence data, which simply do not exist. Etc., etc. My point is simply that the authors will need to do a credible job at the correlative modeling efforts if they wish to then shoot down those approaches as ineffective.

Finally, and most trivially, the manuscript is rife with what many would consider to be grammatical errors, such as split infinitives, comma splices, and subject-verb disagreements.

I present these extensive comments because I consider this topic to be an important one, and because I do not feel that the manuscript (at least in present form) genuinely presents a balanced comparison of the value and utility of the two approaches.

Town Peterson, University of Kansas

Reviewer #3 (Remarks to the Author):

This manuscript focuses on one important scientific question: how to develop more robust predictions of alien species invasions? The authors focused on (sub)tropical birds introduced to Europe as one example, they used some predictor variables related with species' fundamental niches such as body allometry, body temperature, metabolic rates, and feather insulation, and applied eco-physiological mechanistic models (hereafter EMMs), and found that these models can accurately identify the true extent of the geographical area at risk of alien bird invasions. Overall, this manuscript is written clearly. However, I have following major concerns on the method, novelty, and concept advance of the work.

1) Although correlative species distribution models (hereafter SDMs) have limitations, this technique is more widely applied than the EMMs because SDMs are based on predictor variables that are more available than those physiological parameters, which are always unknown for most taxa. In fact, SDMs can also use some key factors related with the species physiological requirements such as upper and lower thermal tolerance (Araújo et al. 2013) to capture physiological limiting processes that may influence species geographical distributions (Kearney & Porter 2009). It has also been observed that correlative SDMs and EMMs can make congruent and accurate predictions of current distributions (Kearney et al. 2010).
2) The authors said that SDMs are based on environmental factors in their native ranges (e.g., Line 31, Line 600-601). In fact, with increasing evidences of the realized niche shift (e.g., Atwater et al. 2018, Early & Sax 2014, etc.), increasing studies have used their entire ranges by incorporating native and invaded ranges together to conduct the predictions in order to capture those niche shift in novel ranges (Broennimann & Guisan, 2008). Just as what the author found in the text (Line 266-268), correlative models could get useful maps when incorporating invasive niches.
3) The authors argued that SDMs estimates climate similarity between native and non-native areas (e.g., Line 90). However, SDMs are not only based on climate variable, but also incorporated factors that are important to species distributions and physiological requirements such as habitat variables (e.g., waters and vegetation as proxy of food availability) (Cardador et al. 2013), species interactions (Wisz et al. 2013), and human affiliations (Cardador & Blackburn 2020). So, one potential explanation on the less accuracy of the correlative SDMs in the present study may be simply due to that the authors only used eight climatic variables (Line 562), but not incorporate more important predictors mentioned above.
4) There are some issues around the niche dynamics quantification and correlative modeling used in the present study. First, regarding the niche dynamics, a previous study had conducted the similar analyses using the European alien birds and found that realized niche expansion is rare but niche unfilling was much more common (Strubbe et al. 2013). But the present work found that the invasive birds in Europe showed a large proportion of realized niche expansion (Line 124-128). These are contrast results, which are needed to be clarified. In addition, regarding the SDM, the authors used an ensemble method based on eight techniques, however, the recent study suggested that there is no particular benefit of ensembles over individual tuned models (Hao et al.

2020).
5) I am particularly concerned with the novelty of the work as there have been many physiological mechanistic models published in recent years (e.g., Kearney et al. 2008, Tingley et al. 2014). Compared with single models such as SDM or EMM, there have been suggestions on integrating correlative niche and mechanistic models together to improve the predictions (Enriquez-Urzelai et al. 2019). Furthermore, except for the traditional modeling based on ecological niches or physiological limitations, the newly-developed approach based on comparing biotic similarity among native communities in the invaded ranges has been used to predict the invasion potentials of established alien birds worldwide (Lovell et al. 2021), which I think is a more promising method and the authors need to compare the performance of the present EMM and the biotic similarity framework. How to combine these different techniques together to develop the next generation of predictive modeling is a more important filed warrants further investigation.

References in the review
Araújo, M. B. et al. Heat freezes niche evolution. Ecol Lett 16, 1206-1219 (2013).
Atwater, D. Z., Ervine, C. & Barney, J. N. Climatic niche shifts are common in introduced plants. Nat Ecol Evol 2, 34-43, (2018).
Broennimann, O. & Guisan, A. Predicting current and future biological invasions: both native and invaded ranges matter. Biol Lett 4, 585-589, (2008).
Cardador, L. & Blackburn, T. M. A global assessment of human influence on niche shifts and risk predictions of bird invasions. Global Ecol Biogeogr 29, 1956-1966, (2020).
Cardador, L., Sardà-Palomera, F., Carrete, M. & Mañosa, S. Incorporating spatial constraints in different periods of the annual cycle improves species distribution model performance for a highly mobile bird species. Divers Distrib 20, 515-528, (2014).
Early, R. & Sax, D. F. Climatic niche shifts between species' native and naturalized ranges raise concern for ecological forecasts during invasions and climate change. Global Ecol Biogeogr 23, 1356-1365, (2014).
Enriquez-Urzelai, U., Kearney, M. R., Nicieza, A. G. & Tingley, R. Integrating mechanistic and correlative niche models to unravel range-limiting processes in a temperate amphibian. Global Change Biology 25, 2633-2647, (2019).
Hao, T., Elith, J., Lahoz-Monfort, J. J. & Guillera-Arroita, G. Testing whether ensemble modelling is advantageous for maximising predictive performance of species distribution models. Ecography 43, 549-558, (2020).
Kearney, M. & Porter, W. Mechanistic niche modelling: combining physiological and spatial data to predict species' ranges. Ecol Lett 12, 334-350, (2009).
Kearney, M. et al. Modelling species distributions without using species distributions: the cane toad in Australia under current and future climates. Ecography 31, 423-434, (2008).
Kearney, M., Wintle, B. & Porter, W. Correlative and mechanistic models of species distribution provide congruent forecasts under climate change. Conserv Lett 3, 203-213, (2010).
Lovell, R. S. L., Blackburn, T. M., Dyer, E. E. & Pigot, A. L. Environmental resistance predicts the spread of alien species. Nat Ecol Evol 5, 322-329, (2021).
Strubbe, D., Broennimann, O., Chiron, F. & Matthysen, E. Niche conservatism in non-native birds in Europe: niche unfilling rather than niche expansion. Global Ecol Biogeogr 22, 962-970, (2013).
Tingley, R., Vallinoto, M., Sequeira, F. & Kearney, M. R. Realized niche shift during a global biological invasion. P Natl Acad Sci USA 111, 10233-10238, (2014).
Wisz, M. S. et al. The role of biotic interactions in shaping distributions and realised assemblages of species: implications for species distribution modelling. Biol Rev 88, 15-30 (2013).

Reviewers' comments:
Reviewer #1 (Remarks to the Author):

In this study, the authors applied both ecophysiological mechanistic models and correlative models to predict the distribution of alien bird species in Europe and to assess their predictive performance in the invasive range for those species. They found the clear niche shift for most alien birds and the poor performance of correlative models in the invasive range. In contrast, mechanistic models can accurately predict the distribution of alien species. Although I am a freshwater fish ecologist, I also collaborated my colleagues to understand the role of different factors on the impacts of alien bird invasions. To my knowledge, bird should be one of the taxa that are suitable for mechanistic models because of the sufficient information. I like this study, because it paved the way of applying mechanistic models to predict the invasion risks of alien species, which is important for future studies and management. I listed my comments, which could be useful to improve this study before it is accepted:

The current Introduction addressed the meaning of studying biological invasions and predicting the invasion risks. Given this study focuses on applying mechanistic models to predict the distribution of invasive species, it would be good to provide some background information about the current process of related researches, so that the readers could have a better understanding of the application of mechanisms models for invasive species.
Reply: We rewrote parts of the introduction to better contextualize our study and contributions in the wider field of spatial invasive species risk assessments (lines 82-94), and focusing on mechanistic models for invasive species in lines 107-115.

Given the first paragraph of the Results section is about realized niche shifts, maybe the analyses of niche shift in the Materials and Methods section (which is currently at the end) can be moved forward to some extent? In addition, it may be good to have a figure summarizing the results of niche shift across all species.
Reply: We have rearranged the methods section and now first present the correlative analysis framework, followed by the mechanistic modeling procedures to better match the flow of the results section. We have added a figure summarizing the niche dynamics analyses (including sensitivity analyses) to the manuscript as Supplementary Figure 1, and the data (niche metrics) as Supplementary Table 1.

Given they have the results of both niche shift and the performance of mechanistic and correlative models, it would be interesting to see the relationship between the magnitude of niche shift and model performance across species. The results would be informative to identify whether models can accurately predict the distribution of alien species in the invasive range based on the results of niche shift.
Reply: Thank you for this suggestion. We have now included these analyses into the ms, showing that correlative (but not mechanistic) model performance declines with increasing magnitude of niche expansion. Please see lines 173-175 and 241-243 in the main ms, lines 63-66 of Supplementary Text 1 and Supplementary Figure 6).

Another interesting result should be the overall geographical patterns of invasion risks for all 20 species based on the predictions of mechanistic and correlative models, respectively. These results could provide insights on the difference in predicted invasions risks using models with good and poor performance.
Reply: Visual representations of the invasion risk predicted by the different methods is given, per species, in Supplementary Figure 3.We assume that with ‘the overall geographical patterns of invasion risks’ the reviewer hints as maps of predicted invasive species richness for Europe. We have therefore provided such maps in Supplementary Figure 2, showing that mechanistic models predict more extensive invasive distributions and generally higher invasive species richness compared to correlative models (line 150-154 and line 228-229).

I think one part that can be added to the current Discussion section could be how the findings of this study can be used to guide the prediction of invasions risks for alien species. For example, whether we can have more reliable predictions for a certain group of species given the clear influence of biophysical traits on the performance of mechanistic models?

Reply: As to the question on taxa, mechanistic models have already been used for predicting invasion risks for e.g. invasive insects (e.g. Kearney et al. 2009 Funct Ecol 23:528-538) and ectotherms (e.g. Tingley et al. 2014 PNAS 28: 10233-10238), but here we show that – based on a limited number of relatively easily obtainable data – mechanistic models can be quickly tailored for a range of endothermic species as well. Hence, we do not have any reason to a priori expect a differing model performance for specific groups of species but instead argue that mechanistic models can and should be more widely used as conservation tools. As suggested by the reviewer, we now more extensively discuss how ecophysiological models can be used in invasive species policy, either 'on their own' or integrated into a wider process-based approach to predicting invasion risks (lines 312-334).

For the figures, it would be good to have the first sentence in the caption to briefly describe of the figure. Currently, the first sentence of Figure 1 shows the meaning of successfully predicting invasion risks, and the first sentence of Figure 2 shows the main results (I rarely see that the caption shows the results).

Reply: Thank you for this comment. All figures captions have been accordingly changed.

In the Materials and Methods section, I would like to see how the 20 bird species were selected, because this manuscript didn't give the description of these bird species. Based on the texts in other sections, I guess the species are those introduced to Europe? It is also important to know how many presence data points are in the native and invasive range for each species.

Reply: We have clarified this in the beginning of the Material and Methods section (lines 434-438). Briefly, we considered all introduced bird species that were a) terrestrial, b) did not have any part of their native range in Europe, c) that had at least five different invasive populations across Europe, and d) for which museum skins were available to measure morphological traits (note that this last condition prevented us from including the red-billed leothrix (*Leiothrix lutea*). Information on native and invasive range occurrence data sample sizes are available at a public GitHub repository at https://github.com/LauraJim/Modeling_bird_invasions, statistical analyses testing for the importance of sample size are reported at line 715-718 and detailed in Supplementary Table 3).

For the paragraph started from Line 405 with "The endotherm model", there is too much detailed information about each trait. Although I know such information is important, I don't think they are as important as other text, such as the modeling setting. There are 959 words in one paragraph, I suggest some texts can be moved to the Supplement Materials if the authors want to keep those words.

Reply: We have severely shortened this paragraph (to 396 words) by only describing key traits a) needed for a non-expert to grasp main NicheMapper inputs, and b) where we obtained species-specific data ourselves (i.e. information on 'default' parameters such animal muscle efficiency of ~25% have been moved to the supplementary material). We also kept the methodological description as succinct as possible (e.g. the spectrophotometry setup). Full methodological details and procedures are now included only as Supplementary Text 2.

I also listed some minor concerns:

Link 115-118, this sentence is better move to the Discussion section, which is based on the findings in this study.

Reply: We would prefer to keep these sentences, as it is not uncommon to end the introduction with a brief summary of the key results. We think these sentences are helpful for focusing the paper to the

reader, but are of course prepared to remove them and reformat into a more ‘traditional’ introduction if the reviewer and editor are of that opinion.

Line 137, they need to mention that “65 and 45%” are the results of intra-specific levels and species-level respectively.
Reply: Given requested re-analyses, this part of the results section has been partly rewritten, but we made sure to be clear about which statistics refer to which model variants, please see lines 146-150.

Line 548, I think “Intraspecific-level models predictive accuracy” should be changed to “The predictive accuracy of intraspecific-level models”?
Reply: Thank you for this suggestion, we have changed the text accordingly (lines 704-706).

Chunlong Liu

Reviewer #2 (Remarks to the Author): I read this manuscript with considerable anticipation, particularly given the list of authors--I genuinely enjoy reviewing papers when I am able to learn from them at the same time. That said, however, I was disappointed in the comparison that was presented--the mechanistic models are presented strongly, but the correlative models developed are weak, such that it is easy to then say that one is better than the other. I think that a more appropriate correlative analysis would reveal that both approaches have merit, and that an ideal analysis will include both approaches.

The following are some key points:
1. "Currently, invasion risk forecasts are usually based on extrapolating environmental tolerances derived from species’ current native ranges to new areas. However, such correlative niche modeling often fails to correctly predict invasive occurrences, leading to risk-ignorant decisions and miscalculation of expected impacts and costs". This paper is not set up as an objective comparison of two parallel methodologies. Rather, it is clear from the outset that the authors have/had made up their minds before the study was initiated, and set out to prove their point. A more appropriate introduction might set out the positive achievements of both methods, as well as the assumptions and drawbacks of each.
Reply: It certainly was not our intention to hold correlative methods subject to a show trial in order to unfairly advance ecophysiological mechanistic modelling as the sole lodestar for assessing species invasion risks, and we welcome the reviewer suggestions for reformulating our aims and findings. We have now rewritten parts of the abstract (where the sentences quoted above come from, lines 30-32), the introduction and the discussion to address this comment. Whereas the previous version of the manuscript may have given the impression that we argued that correlative methods would be *inherently and per definition* incapable of accurately forecasting species invasion risks, we now stress that correlative projections to new ranges constitute an active area of research (lines 82-94), and that the most preferable modeling approach depends on research goals and data availability. We also discuss that recent meta-analyses showing moderate to low SDM accuracy may at least partly reflect how invasive species SDMs have generally been constructed to date, rather than what can potentially be achieved (please see lines 241-243 for the role of niche changes, lines 246-257 on the quality and availability of occurrence data, lines 259-271 on the selection of relevant accessible/background areas, lines 273-284 and 236-239 for a discussion on climate input data, and lines 286-288 and 296-300 for possible alternative modeling strategies).

Line 36 -- Focusing exclusively on thermal niches represents a clear and significant drawback of the mechanistic approach presented. The authors might well comment on the frequency with which non-thermal dimensions are most important (limiting) across birds. My understanding is that non-thermal

dimensions are indeed frequently important, and yet they are entirely ignored in the approaches presented.

Reply: Thank you for this comment. We now discuss the risk of invasion risk overprediction in the warmer and arid parts of Europe, as we here indeed do not explicitly tackle the potential role of water availability on bird evaporative cooling capacity and hence the risk of death by hyperthermia (lines 236-239).

3. "While such correlative species distribution models (SDM) perform well in characterizing the natural distributions of species within their current range[15], multiple statistical and eco-evolutionary issues arise when models are transferred to new areas or time periods, introducing uncertainty into model forecasts and decreasing reliability of forecasts..." Again, the emphasis appears to be on "shooting down" the correlative approaches, rather than on presenting an objective comparison of the two approaches. For instance, although the authors are abundant in citing studies that document drawbacks of the correlative approaches, they have not cited a review of the two methods that pointed out some serious concerns (re overparameterization) of the mechanistic approaches--Peterson, A. T., M. Papeş, and J. Soberón. 2016. Mechanistic and correlative models of ecological niches. European Journal of Ecology 1:28-38. I will apologize for pointing the authors toward one of my own publications, but it does genuinely point to problems with the mechanistic approaches.

Reply: Please see our response to comment #1, this part of the introduction has been rewritten, and the quoted sentences have been removed from the manuscript. For example, we discuss potential drawbacks of our mechanistic modelling relating to model complexity and data demands (e.g. lines 231-239, 218-220) and strengths and opportunities for correlative invasion risk SDMs (e.g. lines 83-86, 90-94, 286-288, 296-300).

4. "Also, presumed relationships between correlative estimates of climatic suitability and the life-history traits and mechanisms that determine species persistence have been found to be questionable at best" Again, this seems to be a gratuitous potshot at the correlative methodology: the same sort of generalization could be aimed at the simplifications involved in the mechanistic modeling process, but it is not.

Reply: As per above, these sentences have been removed from the manuscript as this part of the introduction has been fully rewritten to provide a fairer comparison of different methods.

5. "Using species' realized niches for forecasting invasion risks is likely to result in..." This is incorrect: correlative modeling approaches *attempt to* approximate the fundamental, not the realized, niche.

Reply: Also these sentences are no longer part of the revised introduction. Please see lines 90-94, where we clarify that some correlative models do indeed aim at approximating species' fundamental niches.

However, the approaches used by the authors of this manuscript are clearly going to replicate/estimate the realized niche, and not the fundamental. See comments below on this point.

Reply: We have substantially revised our correlative SDM methodology to address the concerns on realized versus fundamental niches estimation raised by the reviewer. Briefly, we reran the models using an alternative set of modeling algorithms and threshold selection choices that aimed at approximating species' fundamental niches (briefly: climate PCA axes based on all BioClim variables as predictor variables on lines 456, 472-476, selection of modeling algorithms on lines 495-509), use of specificity, sensitivity and AUC ratio as model evaluation criteria on lines 527-537.) Please see our response to the more detailed comments and suggestions about methodology made by the reviewer below.

6. "Correlative models based on native range occurrences accurately described species' native distributions (Supplementary Material 1), but when extrapolating these models to Europe to obtain invasion risk forecasts, they incorrectly delineated the whole continent as unsuitable for 18 out of the 20 species studied here (Fig. 2)." Here, much more more information is needed about how extrapolations

were carried out... E.g., was a MOP-style assessment made of the extrapolation risk? how were model predictions extended to non-analog conditions? etc.

Reply: We had already investigated extrapolation to environmental conditions outside of the model training range by conducting a MESS analysis (this may not have been very clear in the previous version of the manuscript, but it was presented in the Supplementary Materials of the previous submission). We have now expanded this analysis.

***(1)** About identifying areas with 'novel' conditions*: the reviewer suggests to use the 'MOP'-method, but we prefer the related (and more conservative) MESS method, because it requires only information that we can objectively assess. MOP, in contrast, requires the subjective choice of 3 parameters, and when the user wishes to include the entire possible range of climatic distances, MOP most closely matches the similarity surface produced by MESS.

***(2)** About projecting models to 'novel' conditions*: we now use both 'extrapolation' and 'clamping' to novel environments. We initially used 'extrapolation' only, as restricting the analyses to analogous climates (i.e. removing all areas identified as novel by the MESS analyses) already showed that the mechanistic models outperformed the correlative approaches. Put differently, even when eliminating extrapolation risk by considering analogous invasive climates only, correlative models performed comparatively poorly. We now show (lines 175-179, 520-526, Supplementary Table 3) that there is little difference in model performance when allowing extrapolation or using clamping.

(3) *To improve reporting* of all used SDM procedures, we adopted the ODMAP (Overview, Data, Model, Assessment and Prediction) protocol (Zurell et al. 2020 Ecography 43(9): 1261-1277), included as Supplementary Table 6.

7. "Possible reasons for this mediocre performance are many, but most prominently include the inherent assumption of realized niche conservatism..." My 2011 review of niche conservatism is cited here, but incorrectly. Correlative model transfers indeed depend on an assumption of niche conservatism, but not of the realized niche! Rather, given the definition of the realized niche (= fundamental niche reduced by accessible conditions to the existing niche, which is then further reduced by biotic interactions to the realized niche), any assumption of realized niche conservatism would be folly, and none of my work has made any such assertion.

Reply: We have rewritten this paragraph in full, and the sentences referred to by the reviewer have been omitted from the manuscript. Instead, we now discuss how the correlative modeling choices we made (e.g. pertaining to the selection and cleaning of occurrence data (lines 251-257), our generic approach to delineating model calibration areas (lines 259-271) and the use of coarse-scaled versus microclimate predictors (lines 273-284) may influence the accuracy of our SDM implementation. As suggested above by the reviewer, we now point out that correlative and mechanistic models both have merits, based, among others, on the availability of input data and the level of ecological knowledge on the species under consideration (e.g. lines 83-94, 243-257, 259-271, 286-305).

8. "Ecophysiological models only use occurrence data from the invaded range to evaluate forecasts of invasion risk, while correlative SDMs rely on native-range occurrences also for model calibration." This point is particularly important--there is no real comparative evaluation of performance of two different approaches here! Rather, the comparison is between apples and squid, in my opinion. That is, how can one compare the performance of two approaches when the data on which the two are based are entirely different? Indeed, it appears that different environmental datasets were also employed in the two approaches... so we are reduced to comparing digital images of apples to poetry about squid.

Reply: We believe this comment stems from a misunderstanding of our previous (probably insufficiently clear) sentence, which has now been removed. We meant that ecophysiological models do not use occurrence data (from the invaded range or other), except for evaluating the accuracy of the forecasts of invasion risk. SDMs use native range occurrences to derive species-environment correlations (and then they are also evaluated on the invaded range), while NicheMapper employs a biophysical simulation – these different classes of models indeed have different inputs but both aim at

correctly predicting where invasive species are likely to establish. Our paper is not the first to make such a comparison – e.g. also Tingley et al. (2014, PNAS 111 (28) 10233-10238) used the percentage of correctly identified invasive occurrences of cane toads (*Rhinella marina*) in Australia to argue that NicheMapper could more accurately predict the full area at risk of invasion compared to a correlative SDM (maxent).
We do agree with the reviewer that the environmental datasets used can be an important source of discrepancy between model outcomes. We followed the convention of fitting SDMs with raw WorldClim data (representing climate averages at a 2m reference height) while NicheMapper uses its built-in microclimate model to convert these and other weather data into conditions actually experienced by animals. There is recent and increasing evidence that including microclimate predictors into SDM can affect their performance. We now discuss in the manuscript that the higher accuracy of NicheMapper forecasts we find here does not imply that it is an 'inherently superior' technique, and that more research is needed for identifying modeling procedures and conditions that most strongly influence the performance of different forecasting approaches (lines 243-257, 264-271, 282-284, 286-288, 296-300).

9. "Here, we first ran such a species-level model, which was then subjected to a sensitivity analysis uncovering the most influential variables. These variables were then considered in an intraspecific-level model accounting for trait variation." I consider such a sensitivity analysis to be crucial, yet no information was presented as to what sensitivity analyses were run: I would assume that parameter estimates were varied by small increments around the central estimate, yet varying by 1% or 5% or what?
Reply: More details on the sensitivity analyses were presented later in the paragraph. We have now clarified this in the text by adding 'see Latin Hypercube sampling below', lines 647-649). Later in the paragraph, we had written that 'Parameter values were allowed to vary within the range of values contained within our empirical measurements or present within the literature surveyed'. Minimum and maximum values for each trait and species were presented in the Supplementary Material, but we have now added an extra Supplementary Table 7 (lines 681-682), where we detail for each species and traits how much parameter values were varied around the central estimate (expressed in percentages). Body temperature was the trait that was varied the least (on average by ~5%), median percentage of variation was ~25%.

And was each parameter varied independently or were they varied in tandem? And with what precision was each original parameter estimate known? Many appear to be very general assumptions. In the Peterson et al. paper in European Journal of Ecology paper mentioned above, we performed such an analysis using the NicheMapper software, and found that the results were extremely sensitive to specific parameter values.
Reply: Parameters were varied simultaneously ('in tandem'), please see lines 684-686 where we present the Latin Hypercube as an efficient technique to representatively sample the parameter space. A similar approach was followed by Wang et al. (2018, Ecology 99: 2721-2730) for assessing NicheMapper sensitivity when applied to estimating water consumption rates of woolly mammoths. Precision of the original parameter estimates varies according to trait and species, and are given in Supplementary Table 7. In our sensitivity analyses, we focused on how varying parameters influenced the predictive accuracy of invasion risk forecasts (i.e. which geographical areas that have climates that are within or outside of a species' fundamental thermal niche). We did not assess how varying parameters influenced 'raw' predictions of required energy output, but note that Mathewson et al. (2015, PLoS ONE 10(8): e0136677) carried out sensitivity analyses (for a NicheMapper model on whooping cranes *Grus americana*) where input variables were (separately) varied with +/- 10%, which typically resulted in a change in predicted metabolic rates of less than 10% (their Table 7). The European Journal of Ecology example quoted above by the reviewer is a bit more difficult to interpret. It presents an elegant but simple exercise where two parameters of a published NicheMapper model for elk (*Cervus elaphus*) were slightly varied, resulting in changes in predicted evaporative water loss. It is a bit difficult to judge the

magnitude of change from their Figure 4, but the scale of the figure varies from 408 to 415 water loss in kg per year – suggesting a maximum variation in predicted water loss of less than 1%...?
Our finding that NicheMapper performance is mainly governed by a few key traits only and that models parametrized on information gleaned from museum specimens, allometric scaling relationships and literature data can result in useful forecasts of invasion risk is, we argue, one the most interesting results of our study, suggesting mechanistic models may be more broadly applicable than previously assumed.

10. "Species distribution modeling was done using the 'sdm' the ensemble modelling framework140. We applied eight different modelling algorithms: generalized linear models (GLM), generalized additive models (GAM), random forest (RF), maximum entropy (MaxEnt), maximum likelihood (MaxLike), flexible discriminant analysis (FDA), multiple adaptive regression splines (MARS) and generalized boosted models (GBM)." A long series of methodological decisions will plague any attempt to argue that the correlative models in this study are able to reconstruct the fundamental niche (which is the only niche model that can or should be used for model transfers). The choice of algorithms includes many approaches that will estimate complex response shapes, when fundamental niches are likely simple and convex (see https://www.biorxiv.org/content/biorxiv/early/2021/01/27/2021.01.25.428165.full.pdf). No information is presented about how calibration areas were selected, even though that decision is crucial to model success (https://www.sciencedirect.com/science/article/pii/S0304380011000780?casa_token=dAHhLbHyE88AAAAA:oR-c5iPJSiE4e4Au0y7wZCuvPZlaqe-Jy4aSsMJ4SJdVOEPiLnnqlBDz4iga804FtKPAtxft7Q) and to appropriate consideration of uncertainty regarding niche limits (https://onlinelibrary.wiley.com/doi/pdf/10.1002/ece3.6359). TSS approaches are used for model evaluation, even though they require absence data, which simply do not exist. Etc., etc. My point is simply that the authors will need to do a credible job at the correlative modeling efforts if they wish to then shoot down those approaches as ineffective.
Reply: We have completely revamped the correlative analyses in response to the suggestion of the reviewer. **First**, we now use different modeling algorithms. (A) An envelope model, which assumes a regular shape of the fundamental niche in the environmental space and that determines the parameters of that shape based on environmental conditions associated with known presences. We selected the novel fundamental niche ellipse model suggested by the reviewer above. These analyses have been run by Dr Jiménez, lead author on the manuscript introducing this method to the scientific community and now a co-author on this revised manuscript. (B) A statistical algorithm, namely a logistic Generalized Linear Model (GLM), as Qiao et al. (2019, Ecography 42: 521-534) found this method best approximated virtual species' fundamental niches. (C) A machine-learning algorithm, namely Bayesian Additive Regression Trees (BART), a novel, flexible approach to fitting a variety of regression models while avoiding strong parametric assumptions, recently introduced in ecological modeling with very promising results (Carlson, 2020, Methods in Ecology and Evolution 11: 850-858). Please see lines 120-124 and 497-509.
Second, we calculated the Potential Niche Truncation Index (Brown & Carnaval, 2019, Frontiers of Biogeography: e44158) for all species, a metric that quantifies the risk that occurrence data cannot fully represent the fundamental niche because of a limited availability of climates across the geographical area in which the models are calibrated (lines 544-549). While for 3 out of the 20 species this risk was moderate, and for four species high, niche truncation was not correlated with model predictive accuracy (lines 172-173, 267-270, and Supplementary Table 3). We now discuss explicitly how our selection of the calibration area may affect our correlative model predictions (lines 259-271).
Third, we have changed the set of metrics we use for model evaluation. First, instead of the native range TSS threshold, we now evaluate the invasive range forecasts based on both a 2.5 and a 5% training omission threshold (lines 527-529). This allowed us to recalculate the 'sensitivity' of the forecasts (i.e. success in correctly predicting invasive occurrences) and their 'specificity' (i.e. correctly identifying locations that are currently without invasive occurrences, which is informative). In addition, for correlative model invasion risk forecasts, we now also calculate the suggested 'partial area under the

receiver operating characteristic curve' (pROC; Peterson et al. 2008 Ecological Modelling 213:63-72), which prioritizes omission error over commission error in estimating model robustness (lines 532-535). Please also see our replies to related methodological comments above, where we discuss our changes to selecting and applying climate predictors (lines 455-456, 472-475), the selection of calibration areas (456-471), the use of spatial cross-validation of models (lines 510-519), the use of extrapolation and clamping to predict to 'novel' environments (lines 520-526) and restricting model evaluation to similar climates only (through MESS analysis, lines 537-544) and the consideration of potential niche truncation in the native range (lines 544-549).

Finally, and most trivially, the manuscript is rife with what many would consider to be grammatical errors, such as split infinitives, comma splices, and subject-verb disagreements.
Reply: All authors have checked the manuscript to identify and correct grammatical errors, including a native English speaker.

I present these extensive comments because I consider this topic to be an important one, and because I do not feel that the manuscript (at least in present form) genuinely presents a balanced comparison of the value and utility of the two approaches.
Reply: Through our revised modeling procedures and revised contextualization of our goal and results, we hope we have adequately addressed all these comments, and we thank the reviewer for helping us improve the manuscript.

Reviewer #3 (Remarks to the Author):
This manuscript focuses on one important scientific question: how to develop more robust predictions of alien species invasions? The authors focused on (sub)tropical birds introduced to Europe as one example, they used some predictor variables related with species' fundamental niches such as body allometry, body temperature, metabolic rates, and feather insulation, and applied eco-physiological mechanistic models (hereafter EMMs), and found that these models can accurately identify the true extent of the geographical area at risk of alien bird invasions. Overall, this manuscript is written clearly. However, I have following major concerns on the method, novelty, and concept advance of the work.

1) Although correlative species distribution models (hereafter SDMs) have limitations, this technique is more widely applied than the EMMs because SDMs are based on predictor variables that are more available than those physiological parameters, which are always unknown for most taxa. In fact, SDMs can also use some key factors related with the species physiological requirements such as upper and lower thermal tolerance (Araújo et al. 2013) to capture physiological limiting processes that may influence species geographical distributions (Kearney & Porter 2009). It has also been observed that correlative SDMs and EMMs can make congruent and accurate predictions of current distributions (Kearney et al. 2010). Compared with single models such as SDM or EMM, there have been suggestions on integrating correlative niche and mechanistic models together to improve the predictions (Enriquez-Urzelai et al. 2019).
Reply: We largely agree with these comments, and (also in response to comments of Reviewer #2) we have rewritten parts of the introduction (lines 82-94) and discussion (lines 243-257, 273-284, 286-305) to stress that different modeling approaches have different strengths, weaknesses and applicability. For example, when modeling current distributions, both SDMs and other approaches often work well – however, when forecasts to novel environments are the aim, models predictions tend to diverge (reviewed in Tourinho & Vale 2022, Integrative Zoology, in press https://doi.org/10.1111/1749-4877.12618). We note that one of the main findings of our work is that it is feasible to obtain useful mechanistic forecasts based on readily available or obtainable physiological data, pointing to a wider applicability of ecophysiological modeling than often assumed (lines 300-305, 329-334).

On the integration of physiology into SDMs and 'hybrid' models (also mentioned in comment #5): hybrid models are obtained either by incorporating microclimatic ('mechanistic') predictors into SDMs (as we now discuss in lines 273-284), or by using mechanistic model outputs as input for correlative models (e.g. Mathewson et al. 2016 Glob Ch. Biol 23:1018-1064). Yet, for our study goals – i.e. identifying all areas with climates suitable to a species - any modeling approach that relies on native range occurrence data for model training runs the risk of underpredicting suitable areas, as because of e.g. dispersal limitations or biotic interactions, contemporary occurrence data frequently do not reflect all climates fundamentally tolerable to a species (see e.g. the quoted Atwater et al. 2018 reference; but see lines 87-94 where we discuss ongoing research on the potential feasibility of approximating fundamental niches using occurrence data). Indeed, in their recent review of correlative, mechanistic and hybrid models Tourinho & Vale (2022) argue that 'the best approach is highly context-dependent' and that 'when the focus is on assessing something close to the fundamental (NF) or existing (NE) niches […] , then physiological mechanistic models become the best choice." We argue that our results support the use of ecophysiological models for assessing invasions risks, an application whereby it is important not to underpredict potential areas at risk of invasion (Srivastava et al. 2019 CAB Reviews 14 doi: 10.1079/PAVSNNR201914020).

2) The authors said that SDMs are based on environmental factors in their native ranges (e.g., Line 31, Line 600-601). In fact, with increasing evidences of the realized niche shift (e.g., Atwater et al. 2018, Early & Sax 2014, etc.), increasing studies have used their entire ranges by incorporating native and invaded ranges together to conduct the predictions in order to capture those niche shift in novel ranges (Broennimann & Guisan, 2008). Just as what the author found in the text (Line 266-268), correlative models could get useful maps when incorporating invasive niches.
Reply: We have now clarified in the ms that our prime focus is on 'pro-active' modelling of invasion risks, e.g. identifying species that currently are not yet invasive (or at a very early stage of invasion) but that are likely to become successful is they were to be introduced to a given area (lines 91-94, 288-293). These are, for example, potential invaders identified through horizon-scanning exercises – such as several species that are on the List of Invasive Alien Species of [European] Union Concern but have currently no recorded invasive presences.

3) The authors argued that SDMs estimates climate similarity between native and non-native areas (e.g., Line 90). However, SDMs are not only based on climate variable, but also incorporated factors that are important to species distributions and physiological requirements such as habitat variables (e.g., waters and vegetation as proxy of food availability) (Cardador et al. 2013), species interactions (Wisz et al. 2013), and human affiliations (Cardador & Blackburn 2020). So, one potential explanation on the less accuracy of the correlative SDMs in the present study may be simply due to that the authors only used eight climatic variables (Line 562), but not incorporate more important predictors mentioned above.
Reply: Distribution models, mechanistic and correlative approaches, that are aimed at characterizing species' distributions at large spatial scales mostly employ climate predictors because, at continental scales, climate is considered the principal driver of where species can occur (e.g. Root 1998 Ecology 69: 330-339, Thomas 2010 Divers. Distrib. 16: 488-495). At more local scales, habitat and land-use become more relevant, and interact with biotic interactions to determine more fine-grained patterns of occurrence within the broader, climatically suitable area (Pearson & Dawson 2003 Glob Ecol Biogeogr 12:361-371). As our goal here is to forecast potential range limits of invasive species, we argue that our choice of including only climate predictors is justified. However, and also in response to a suggestion of Reviewer #2, we now include all available bioclimatic variables (summarized into PCA axes, lines 472-474, 495-497), as this approach has been suggested to increase model transferability (Petitpierre et al 2017 Glob Ecol Biogeogr 26:275-287).
We argue that including habitat and land-use variables is unlikely to result in improved predictions of invasive occurrences outside of areas predicted as climatically suitable. For example, the quoted Cardador & Blackburn (2020) paper finds that including anthropogenic predictor variables into SDM

model training affects estimates of niche unfilling, rather than niche expansion. Put differently, habitat acts as a ‘filter’ on climatically suitable areas. Different approaches have been proposed to integrate habitat information, for example running a separate SDM using only habitat variables present in areas designated as climatically suitable (e.g. Fournier et al. 2017 Glob Ecol Cons 12: 215-226 for invasive hornets) or simply marking as ‘unsuitable’ each grid pixel where a predefined required habitat is not present (e.g. Naimi et al. in press Glob Change Biol, https://doi.org/10.1111/gcb.16310 for invasion risks of traded birds species under climate and land-use change). We now discuss such integration in lines 312-334).

4) There are some issues around the niche dynamics quantification and correlative modeling used in the present study. First, regarding the niche dynamics, a previous study had conducted the similar analyses using the European alien birds and found that realized niche expansion is rare but niche unfilling was much more common (Strubbe et al. 2013). But the present work found that the invasive birds in Europe showed a large proportion of realized niche expansion (Line 124-128). These are contrast results, which are needed to be clarified. In addition, regarding the SDM, the authors used an ensemble method based on eight techniques, however, the recent study suggested that there is no particular benefit of ensembles over individual tuned models (Hao et al. 2020).
Reply: This apparent discrepancy is due to a different set of bird species that is considered in both studies. Given that our focus here is on testing the accuracy of distribution model predictions when forecasted to novel environments, we did not include bird species that have at least part of their native distribution in Europe. Also, since Strubbe et al. 2013, several new invasive species databases have been published (Dyer et al. 2017 Sci Data 4: 170041, Ascensão et al. 2021 NeoBiota 64: 1–21, Abellán et al. 2017 PNAS 35: 9385-9390, Reino et al. 2017 Sci Adv 3: doi: 10.1126/sciadv.1700783), allowing us to include species where (spatial) information was not (sufficiently) available before 2013. These species mainly are (sub)tropical birds introduced as pets (Reino et al. 2017). For the five bird species shared between this paper and Strubbe et al. (2013), there is a strong correlation between measured niche expansion ($r=0.96$; $p= 0.0083$). Please see lines 434-438, where we present more information on species selection. Also in response to suggestions of Reviewer #2, we have revamped the ensemble modeling approach, allowing to investigate the performance of fewer key algorithms (lines 120-124, lines 496-).

5) I am particularly concerned with the novelty of the work as there have been many physiological mechanistic models published in recent years (e.g., Kearney et al. 2008, Tingley et al. 2014).
Reply: A main novelty is that we present one of the first papers that independently validates the accuracy of mechanistic predictions of species’ distributions to unsampled (‘novel’) environments - and the first one to consider endothermic invasive species. There indeed are some studies that apply mechanistic or ‘hybrid’ models to predict likely range shifts under climate change (e.g. the quoted Kearney et al. 2008, 2010 and Enriquez-Urzelai et al. 2019 papers), but these forecast are currently in fact untestable, as they predict possible futures. Biological invasions offer the unique opportunity to evaluate model transferability to novel environments using independent invasive range data. The only paper that can be considered similar to ours is Tingley et al. 2014, which compares (ecophysiological) NicheMapper to (correlative) Maxent for predicting the distribution of cane toad introduced to Australia. To date, ecophysiological approaches have remained largely limited to ectothermic species, as they are assumed to be more directly limited by climate gradients (e.g. Aragón et al. 2009 Glob Ecol Biogeogr 19:40-49, Buckley et al. 2012 Glob Ecol Biogeogr 21:873-885), and because they are considered less challenging to conduct experiments for obtaining physiological data (e.g. the experiments described in Tingley et al. 2014). We are (to our knowledge) the first to show that it is possible to obtain accurate forecasts of species’ ranges using information that is readily available for most endotherm species. We argue this is a timely and important contribution for ecological forecasting, serving as a catalyst for integrating climate-related physiological costs into improved methodologies for forecasting the future of biodiversity in a changing world. Please see lines 83-94 and 107-115, where we provide more context

regarding the use of different modeling techniques for assessing biodiversity dynamics, and lines 312-334 where we discuss how our results can be used in invasive species management.

Furthermore, except for the traditional modeling based on ecological niches or physiological limitations, the newly-developed approach based on comparing biotic similarity among native communities in the invaded ranges has been used to predict the invasion potentials of established alien birds worldwide (Lovell et al. 2021), which I think is a more promising method and the authors need to compare the performance of the present EMM and the biotic similarity framework. How to combine these different techniques together to develop the next generation of predictive modeling is a more important filed warrants further investigation.
Reply: Lovell et al. (2021) use the species compositions of both source and incipient communities to predict how (already established) invaders are likely to spread. They find that biotic resistance is more accurate compared to native-range climate matching. Directly comparing the biotic similarity framework to our ecophysiological approach is however difficult, as it is not possible to rerun the biotic similarity script for Europe at the ~10 km² resolution we used (Lovell et al. 2021: "We used a grid resolution of ~100 km because this is the finest resolution at which expert polygon range maps can be analysed without incurring substantial false presences"). We therefore, for each species considered in our study, extracted the biotic similarity invasion risk for all 100-km² pixels overlapping with Europe from the Lovell et al. 2021 Supplementary Material (using the 'InvasionProbVec' in their R script '5.CalculateGlobalInvasionRisk.R'). This was possible for 18 out of our 20 species (we did not find *Ploceus melanocephalus* and *Poicephalus senegalus*), and is visualized in the figures below. Results show an all-or-nothing pattern whereby either no (7 species) or all occurrences (11 species) were correctly predicted. For these 11 species, biotic resistance however forecasts invasions across extensive parts of Europe, including cold continental and north-European areas that arguably are unlikely to be climatically suitable e.g. to small (sub)tropical birds such as the *Estrilda* species.
We are in agreement with Lovell et al. 2021 in that correlative models, be it our SDMs or their climate matching procedure, often fail to accurately predict invasive ranges. An advantage of ecophysiological models is that they can be used to predict invasion risk for species not yet introduced to a given region, whereas the biotic similarity framework depends on simulating further spread from an already invaded location. However, rather than pitching 'ecophysiology' against 'biotic resistance' as a panacea, we argue it may be more effective to integrate both into process-explicit frameworks, whereby climate-driven physiological costs are used to scale population dynamics models, and using the biotic landscape resistance to inform models' dispersal components. Please see lines lines 312-334 for this discussion.

References in the review.
Araújo, M. B. et al. Heat freezes niche evolution. Ecol Lett 16, 1206-1219 (2013).

Atwater, D. Z., Ervine, C. & Barney, J. N. Climatic niche shifts are common in introduced plants. Nat Ecol Evol 2, 34-43, (2018).

Broennimann, O. & Guisan, A. Predicting current and future biological invasions: both native and invaded ranges matter. Biol Lett 4, 585-589, (2008).

Cardador, L. & Blackburn, T. M. A global assessment of human influence on niche shifts and risk predictions of bird invasions. Global Ecol Biogeogr 29, 1956-1966, (2020).

Cardador, L., Sardà-Palomera, F., Carrete, M. & Mañosa, S. Incorporating spatial constraints in different periods of the annual cycle improves species distribution model performance for a highly mobile bird species. Divers Distrib 20, 515-528, (2014).

Early, R. & Sax, D. F. Climatic niche shifts between species' native and naturalized ranges raise concern for ecological forecasts during invasions and climate change. Global Ecol Biogeogr 23, 1356-1365, (2014).

Enriquez-Urzelai, U., Kearney, M. R., Nicieza, A. G. & Tingley, R. Integrating mechanistic and correlative niche models to unravel range-limiting processes in a temperate amphibian. Global Change Biology 25, 2633-2647, (2019).

Hao, T., Elith, J., Lahoz-Monfort, J. J. & Guillera-Arroita, G. Testing whether ensemble modelling is advantageous for maximising predictive performance of species distribution models. Ecography 43, 549-558, (2020).

Kearney, M. & Porter, W. Mechanistic niche modelling: combining physiological and spatial data to predict species' ranges. Ecol Lett 12, 334-350, (2009).

Kearney, M. et al. Modelling species distributions without using species distributions: the cane toad in Australia under current and future climates. Ecography 31, 423-434, (2008).

Kearney, M., Wintle, B. & Porter, W. Correlative and mechanistic models of species distribution provide congruent forecasts under climate change. Conserv Lett 3, 203-213, (2010).

Lovell, R. S. L., Blackburn, T. M., Dyer, E. E. & Pigot, A. L. Environmental resistance predicts the spread of alien species. Nat Ecol Evol 5, 322-329, (2021).

Strubbe, D., Broennimann, O., Chiron, F. & Matthysen, E. Niche conservatism in non-native birds in Europe: niche unfilling rather than niche expansion. Global Ecol Biogeogr 22, 962-970, (2013).

Tingley, R., Vallinoto, M., Sequeira, F. & Kearney, M. R. Realized niche shift during a global biological invasion. P Natl Acad Sci USA 111, 10233-10238, (2014).

Wisz, M. S. et al. The role of biotic interactions in shaping distributions and realised assemblages of species: implications for species distribution modelling. Biol Rev 88, 15-30 (2013).

Lovell et al. (2021): predictions of invasive species occurrence in Europe based on biotic resistance. Regular dots represent the center of 100x100 km grid pixels. Black dots indicate areas where introduced birds are not predicted to invade, yellow pixels indicate areas at risk of invasion. Red dots represent (raw) contemporary bird occurrences across Europe.

Acridotheres cristatellus

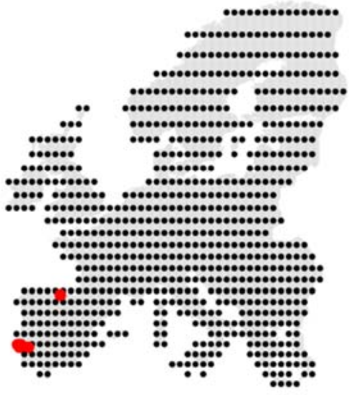

Acridotheres tristis

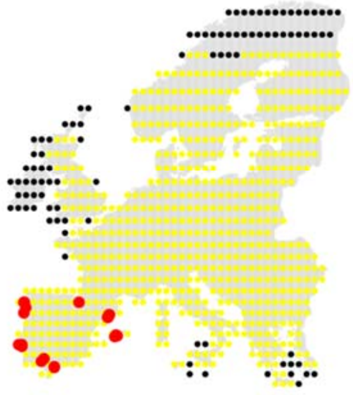

Agapornis fisheri

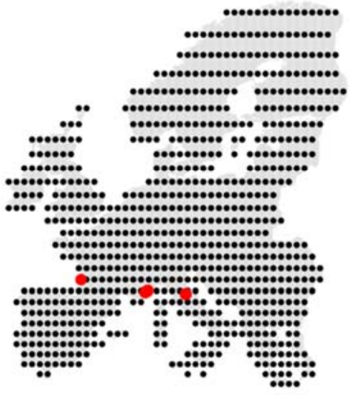

Agapornis personatus

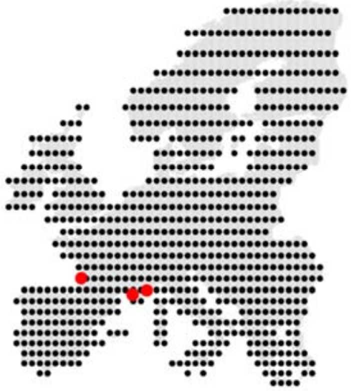

Agapornis roseicollis

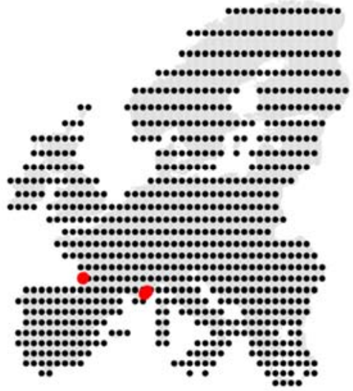

Amandava amandava

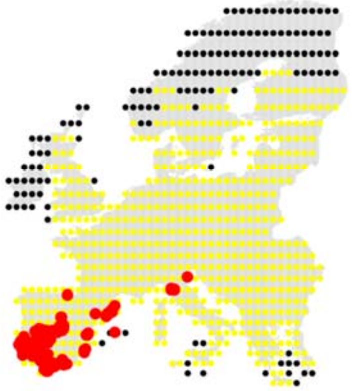

Aratinga acuticaudata

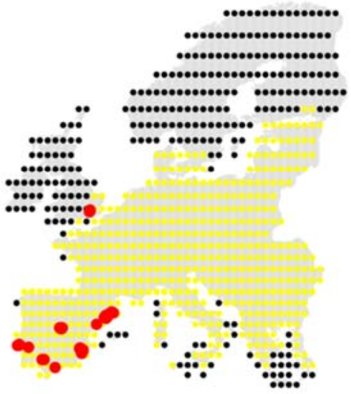

Chrysolophus pictus

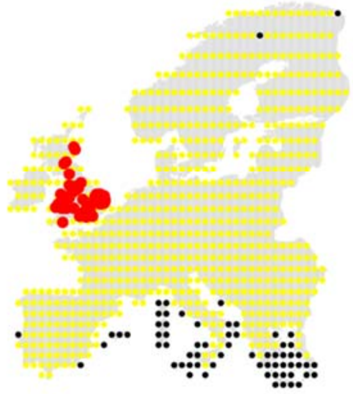

Estrilda astrild

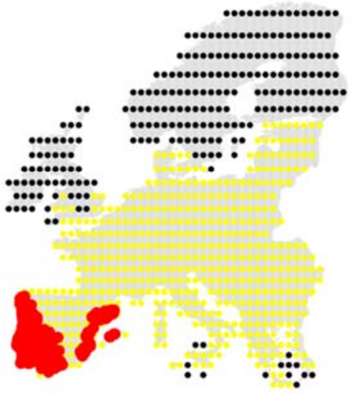

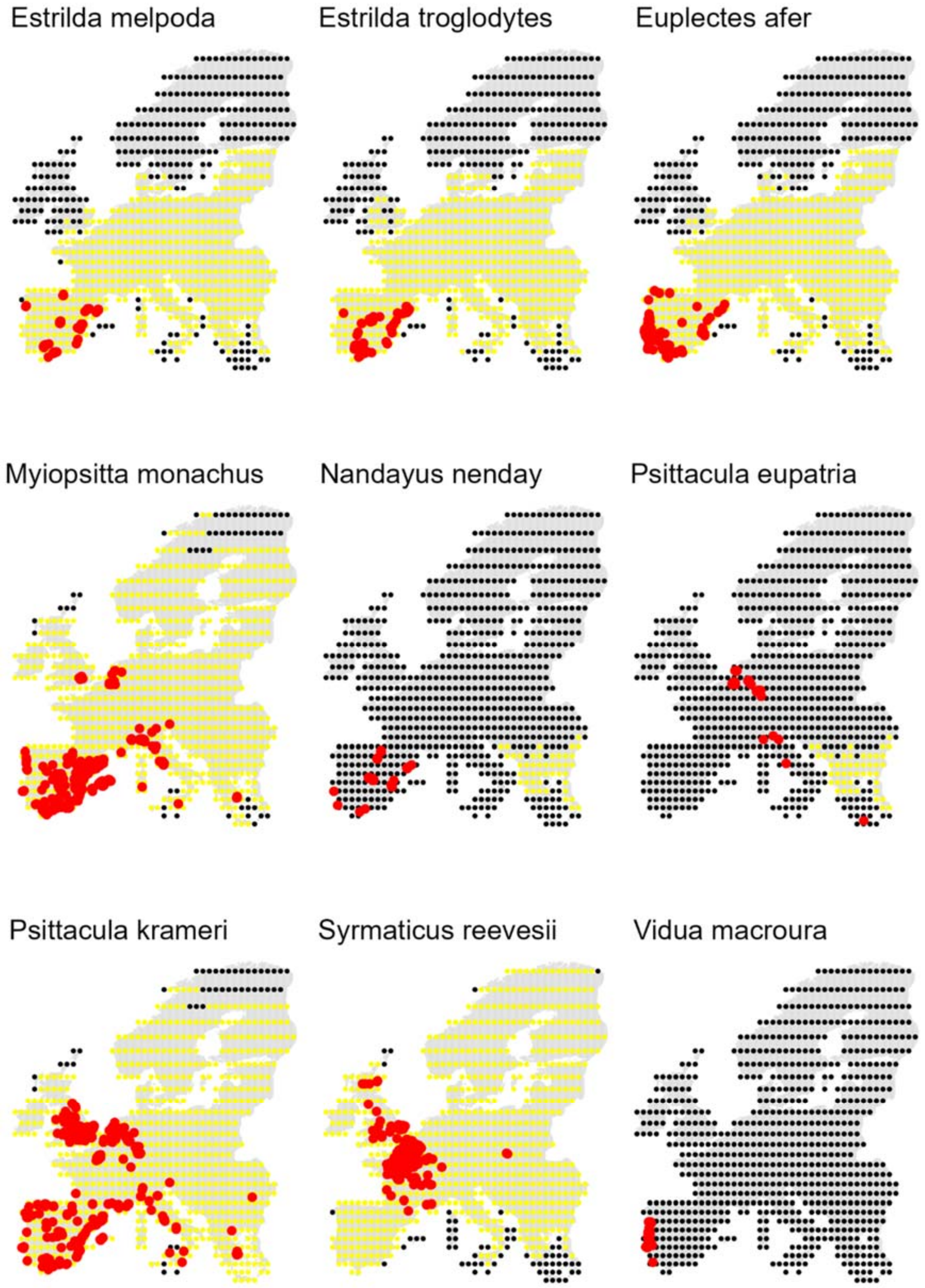
Estrilda melpoda
Estrilda troglodytes
Euplectes afer
Myiopsitta monachus
Nandayus nenday
Psittacula eupatria
Psittacula krameri
Syrmaticus reevesii
Vidua macroura

Reviewer comments, second round –

Reviewer #2 (Remarks to the Author):

The authors have certainly undertaken their revision of this manuscript with gusto, such that I was quite impressed with the changes that they made in response to the first round of reviews.

The manuscript, while much improved, remains a bit off balance. Two clear gaps are as follows:

1. MESS is not an acceptable substitute for MOP. The reason is clear, if one reads the original MOP paper: MESS refers the calculation of the distances to the centroid of the reference cloud, whereas MOP refers those calculations to the nearest part of the reference cloud. The authors made brief mention of wishing to take into account the full reference cloud, which may mean that they were aware of this point, but they have not appreciated that the reference clouds may be highly irregular, such that a distance to a centroid can be very misleading. What MATTERS is how novel the particular set of conditions manifested at a site of interest among the conditions manifested across the reference cloud. MESS fails in evaluating this facet of model transfer and evaluation.

2. The authors added sensitivity analyses of their biophysical models, but only partially. That is, they have added in a sensitivity analysis that assesses the relative influences of changing values of different input parameters on the models, BUT they do not assess how that turns into changes in the area identified as suitable by the biophysical models. (They refer to more detail being provided in the Supplementary Text 2, but that was not in the packet of materials provided to me as reviewer._

Overall, this is an interesting contribution, but I think that the conclusions are stated too broadly for the model-transfer test that was set up (i.e., subtropical bird species invading into Europe).

Reviewer #3 (Remarks to the Author):

I appreciate it for the authors great efforts to revise the manuscript especially a substantial of new analyses, which have indeed strengthened the manuscript. In this round of review, I am still mainly concerned with the broad application of ecophysiological modeling and provide some technical suggestions to further improve the correlative modeling construction.
First, I want to say that I never doubted that there are advantages of ecophysiological modeling compared with other correlative approaches. Indeed, the ecophysiological predictor variables would be more likely to reflect the species' fundamental niches, and thus may be able to generate more precise predictions. However, just as what the authors argue that "different modeling approaches have different strengths, weaknesses and applicability", for correlative modeling approaches, scientists have been working hard to continue to develop new methods to improve the model predictive power from techniques to control sampling bias and selection of more predictor variables to reflect more aspects of species niches especially representing their interactions with other biotic and abiotic factors in habitats. As a paper shooting for a high-profile and multidisciplinary journal such as Nature Communications, I just think it is very important to consider the potential general application of the work to a possibly wide field. For instance, the present work only used 20 alien birds in European countries with available ecophysiological data and under other conditions. However, by the year of 2008, there has been about 140 alien birds introduced to Europe and 77 species have established populations. Based on Dyer's recent database on global alien birds (GAVIA database, Dyer et al. 2017, Scientific Data) and the later publications (e.g., Dyer et al. 2017, PLoS Biology), there are more established alien species recorded in Europe. So, although there are approach limitations in correlative species distribution modeling or the environmental resistance model (Lovell et al. 2021 Nature Ecology & Evolution), they can cover more established alien species and thus have broader implications for the early prevention of alien species establishment.
Second, for the construction of the correlative species distribution modeling, I suggest that there are still spaces to improve and be compared with the ecophysiological modeling. For example, the

authors used the 19 climatic variables in the correlative modeling construction. However, biotic interaction factors are important to the improvement of bird species distribution models (e.g., Palacio FX and Girini JM. 2018. Biotic interactions in species distribution models enhance model performance and shed light on natural history of rare birds: A case study using the straight-billed reedhaunter limnoctites rectirostris Journal of Avian Biology 49: e01743.). Unfortunately, the authors did not consider biotic interaction factors or proxy variables in correlative distribution modeling. Although the authors argued in their response letter that including habitat and land-use variables is unlikely to result in improved predictions of invasive occurrences, the authors need quantitative evidences but not only address this in the discussion. Similarly, regarding the potential effects of sampling bias in species occurrence on model performance, although the authors argued this issue in the Discussion section (e.g., line 246-257), I would suggest that the authors need apply more robust approach to account for the potential sampling bias in model constructions. For instance, the authors could consider using the approach of related species (Qiao HJ, Peterson AT, Ji LQ, et al. 2017. Using data from related species to overcome spatial sampling bias and associated limitations in ecological niche modelling Methods Ecol Evol 8: 1804-1812.) or a target group method (Phillips SJ, Dudik M, Elith J, et al. 2009. Sample selection bias and presence-only distribution models: Implications for background and pseudo-absence data Ecol Appl 19: 181-197.).

REVIEWER COMMENTS

Reviewer #2 (Remarks to the Author):
The authors have certainly undertaken their revision of this manuscript with gusto, such that I was quite impressed with the changes that they made in response to the first round of reviews. The manuscript, while much improved, remains a bit off balance. Two clear gaps are as follows:
Reply: We thank the reviewer for their efforts to further improve the modeling undertaken in this ms. We have now replaced our earlier MESS analyses by the suggested MOP approach, and have included the requested mechanistic model sensitivity analysis regarding the part of Europe that is predicted as suitable for invaders.

1. MESS is not an acceptable substitute for MOP. The reason is clear, if one reads the original MOP paper: MESS refers the calculation of the distances to the centroid of the reference cloud, whereas MOP refers those calculations to the nearest part of the reference cloud. The authors made brief mention of wishing to take into account the full reference cloud, which may mean that they were aware of this point, but they have not appreciated that the reference clouds may be highly irregular, such that a distance to a centroid can be very misleading. What MATTERS is how novel the particular set of conditions manifested at a site of interest among the conditions manifested across the reference cloud. MESS fails in evaluating this facet of model transfer and evaluation.
Reply: We thank the reviewer for stressing the importance of carefully considering the shape of the reference cloud (i.e. the set of environmental conditions available to the species across its native range) and its potential implications. We initially selected the MESS approach because of a) its simplicity and b) because visual inspections of the parts of Europe marked as 'similar' to the native range were credible (i.e. mostly considering the southern Mediterranean parts of the Europe as 'similar climate'). We have now replaced the MESS analyses with the proposed MOP approach, using a similarity threshold of 10% (line 614-617). Also according to MOP, for most species, the Mediterranean part of the continent is labeled as 'similar' to the native range (see visualizations in the 'MOP_JPGs' folder on Zenodo). Restricting correlative model forecasts to 'similar' areas only as defined by MOP resulted in slightly higher AUCratio for BART and GLM models (about 5% higher, lines 65-69 in Supplementary Text 1, lines 181-184 in the main ms).

2. The authors added sensitivity analyses of their biophysical models, but only partially. That is, they have added in a sensitivity analysis that assesses the relative influences of changing values of different input parameters on the models, BUT they do not assess how that turns into changes in the area identified as suitable by the biophysical models. (They refer to more detail being provided in the Supplementary Text 2, but that was not in the packet of materials provided to me as reviewer.
Reply: First, we apologize that the reviewer was not able to access Supplementary Text 2. For this submission we double-checked that all files (Supplementary Files, input data and model outputs) are easily accessible to reviewers and readers. Our sensitivity analyses indeed aimed at testing the relative influence of changing parameter values on the models, to identify the most influential variables. We have now added how changing the most influential variables impacts upon the percentage of Europe that is predicted as suitable (lines 197-202 and 769-774), Supplementary Figure 7). Results show that increasing species capacity for generating (higher body mass and BMR) and retaining heat (higher plumage insulation, lower body temperatures) lead to a larger percentage of Europe predicted to be at risk of invasion.

Overall, this is an interesting contribution, but I think that the conclusions are stated too broadly for the model-transfer test that was set up (i.e., subtropical bird species invading into Europe).
Reply: We introduced several new paragraphs in the discussion to better frame our conclusions, and to highlight current obstacles to more widely apply mechanistic models in ecological forecasting, please see lines 312-332, 334-353 and 379-385).

Reviewer #3 (Remarks to the Author):

I appreciate it for the authors great efforts to revise the manuscript especially a substantial of new analyses, which have indeed strengthened the manuscript. In this round of review, I am still mainly concerned with the broad application of ecophysiological modeling and provide some technical suggestions to further improve the correlative modeling construction.

First, I want to say that I never doubted that there are advantages of ecophysiological modeling compared with other correlative approaches. Indeed, the ecophysiological predictor variables would be more likely to reflect the species' fundamental niches, and thus may be able to generate more precise predictions. However, just as what the authors argue that "different modeling approaches have different strengths, weaknesses and applicability", for correlative modeling approaches, scientists have been working hard to continue to develop new methods to improve the model predictive power from techniques to control sampling bias and selection of more predictor variables to reflect more aspects of species niches especially representing their interactions with other biotic and abiotic factors in habitats. As a paper shooting for a high-profile and multidisciplinary journal such as Nature Communications, I just think it is very important to consider the potential general application of the work to a possibly wide field. For instance, the present work only used 20 alien birds in European countries with available ecophysiological data and under other conditions. However, by the year of 2008, there has been about 140 alien birds introduced to Europe and 77 species have established populations. Based on Dyer's recent database on global alien birds (GAVIA database, Dyer et al. 2017, Scientific Data) and the later publications (e.g., Dyer et al. 2017, PLoS Biology), there are more established alien species recorded in Europe. So, although there are approach limitations in correlative species distribution modeling or the environmental resistance model (Lovell et al. 2021 Nature Ecology & Evolution), they can cover more established alien species and thus have broader implications for the early prevention of alien species establishment.
Reply: We thank the reviewer for insisting on a balanced discussion of the pros and cons of different available techniques for obtaining useful predictions of invasion risk, and thus agree with the above comment. In response to this comment and a similar comment of reviewer #2, we have now introduced into the discussion several paragraphs that highlight which obstacles that need be dealt with before mechanistic modeling can be truly broadly applied (please see lines 312-332, 334-353 and 379-385). Hereby we hope that the ms text better conveys our message that mechanistic models can be powerful tools that need to be explored further, without being dismissive about the research that is currently being done on improving correlative forecasts.

Second, for the construction of the correlative species distribution modeling, I suggest that there are still spaces to improve and be compared with the ecophysiological modeling. For example, the authors used the 19 climatic variables in the correlative modeling construction. However, biotic interaction factors are important to the improvement of bird species distribution models (e.g., Palacio FX and Girini JM. 2018. Biotic interactions in species distribution models

enhance model performance and shed light on natural history of rare birds: A case study using the straight-billed reedhaunter limnoctites rectirostris Journal of Avian Biology 49: e01743.). Unfortunately, the authors did not consider biotic interaction factors or proxy variables in correlative distribution modeling. Although the authors argued in their response letter that including habitat and land-use variables is unlikely to result in improved predictions of invasive occurrences, the authors need quantitativeevidences but not only address this in the discussion.
Reply: We have now included habitat and biotic variables into our modeling framework to test our assumption that at broad spatial scales, climate is the primary driver of species' range limits (Pearson & Dawson 2003, Glob. Ecol. Biogeogr. 12: 361-373). Habitat variables were taken from the set of habitat heterogeneity variables derived by Tuanmu & Jetz (2015, Glob. Ecol. Biogeogr. 24:1329-1339) while biotic interaction proxy variables representing nest and resource availability were obtained through the Copernicus Global Land Service (please see lines 575-585). Results of these analyses are in line with our assumption regarding the role of climate: across the native range, the capacity to correctly identify bird occurrences did not differ between models including habitat and biotic interactions variables and climate-only models (i.e. models have similar sensitivity). Habitat and biotic interactions variables did allow to identify native range areas where species are absent despite suitable climates (i.e. higher specificity). The accuracy of forecasts of invasion risk was lower for models with habitat and biotic interaction variables compared to climate-only models. Please see lines 160-165 in the main ms text and lines 45-57 in Supplementary Text 2.

Similarly, regarding the potential effects of sampling bias in species occurrence on model performance, although the authors argued this issue in the Discussion section (e.g., line 246-257), I would suggest that the authors need apply more robust approach to account for the potential sampling bias in model constructions. For instance, the authors could consider using the approach of related species (Qiao HJ, Peterson AT, Ji LQ, et al. 2017. Using data from related species to overcome spatial sampling bias and associated limitations in ecological niche modelling Methods Ecol Evol 8: 1804-1812.) or a target group method (Phillips SJ, Dudik M, Elith J, et al. 2009. Sample selection bias and presence-only distribution models: Implications for background and pseudo-absence data Ecol Appl 19: 181-197.).
Reply: We thank the reviewer for these suggestions aimed at further mitigating the possible effects of bias in the occurrence data used for correlative model training. We explored this issue more deeply and decided to implement a novel 'accessibility bias' correction (lines 568-575). Target-group approaches are not compatible with the fundamental niche ellipse method used here, as this methods aims to include as many random points from the calibration area as possible because these points are used to estimate a density in environmental space from which weights are extracted for each presence. The approach of related species has as potential drawback that selecting a set of 'close phylogenetic relatives with overlapping fundamental niches' is far from straightforward. The accessibility bias approach that we use instead applies a Bayesian approach to estimate how sampling rates vary as a function of proximity to bias factors such as the presence of roads, rivers, airports and cities. While we explored introducing the accessibility bias into the fundamental niche ellipses (by adjusting the weight function that modifies the probabilities of observing different environmental combinations by the accessibility bias index), this turned out to be not feasible at the moment and needs more research. Hence, the accessibility bias was only used for BART and GLM methods, whereby spatially explicit estimates of sampling rates were included as covariate when training SDMs on the native range. This bias layer was then set to zero for invasive range predictions. In addition, we downloaded the most recent available GBIF occurrence data, and used the R package 'CoordinateCleaner' to verify the reliability of the available occurrences (lines 505-509).

Reviewer comments, third round –

Reviewer #2 (Remarks to the Author):

The authors have made a very credible and significant effort to respond to the comments that they got from the reviewers. I was more than happy to see that they added essentially all of the additional analyses that I had suggested, and that they made changes that were quite a bit more than trivial. Thanks to the authors for taking reviewers' comments so much to heart. My only reservation, which is based in my being a grammar nerd, is that the authors have used a number of split infinitives, which I do not like... this is a trivial point, and the Editors can decide what to do about it, if anything. More to the point, I think that this manuscript is now a significant contribution, and I am completely "OK" with it being published in Nature Communications.

Town Peterson, University of Kansas

Reviewer #3 (Remarks to the Author):

The authors have conducted substantial efforts to revise their manuscript by providing a deeper discussion on the pros and cons of correlative and mechanistic models, conducting new analyses by incorporating biotic interaction variables, and addressing the potential sampling bias issue based on an accessibility bias approach. I think the manuscript has been greatly improved and I suggest that it is ready to be accepted.

Responses (in blue font) **to the comments by the editor and the reviewers**

Reviewer #1 (Remarks to the Author):

The authors satisfactorily addressed all my previous comment, and I can now recommend this nice manuscript for publication. I have one last suggestion, though. In your rebuttal, you explain why you chose to group the plots per 2 into spatial blocks as follows:

"However, at the time our platform was built, there were indeed some natural gradients at the site (e.g. plant community composition, depth from soil surface to bedrock, microtopography) that made two plots within one block more similar to each other than to plots in other blocks. Moreover, each block (an ambient plot and a paired warming plot) was controlled by a separate computer program and datalogger system (as well as soil temperature and moisture sensors). Whether the warming cable for the warming plot in each block was switched on or off was determined independently by the temperature difference between the two plots (warming plot vs. ambient plot) within a block. In other words, the two paired plots within each of the four blocks were monitored (soil temperature and moisture) and controlled (turning on/off heater) independently. In this case, "plot nested within block" should be served as a random effect in the LMM models, and we have redone data analyses."

These are two very valid arguments for your choice of blocking: i) there are natural gradients in the field site, ii) the paired warmed and control plots were both steered by a single data logging system coupled to a warming system, which could cause data dependency (suppose, for example, that one of the systems malfunctions for a while). So why not just add this information to the methods section? Now the first reference to "block" is in the "statistical analysis" section. Other than that, the reader has to go to Supplementary Figure 1 to see what is meant by "block". You are making the reader work a bit hard here.

Response: Thank you very much for your approval of our revision work. According to your further comments, we have added the description that you offered to explain why we chose to group the plots per 2 into spatial blocks in the Methods section. Please see the lines 202-205.

Reviewer #3 (Remarks to the Author):

Review of the manuscript titled "Climate warming causes mismatches in plant-microbe fauna phenology", by Yin et al.
My review is focused on camera-NDVI retrieval, as specifically asked by the editor.

The authors clarified all points raised in the processing of camera-NDVI. I think all these steps were needed in order to make the workflow clear, which was clearly not the case for the first version of the manuscript.

It is noteworthy however that the raw camera NDVI values fall within the range of spectral NDVI already before any correction. This must certainly have to do with some site/instrumental-specific feature. Howerever, at present, as far as camera-NDVI is concerned, the manuscript and the data behind appear now sound. I suggest the publication of the manuscript.

Response: Thank you very much for your approval of our revision work. With your great help, our manuscript had been greatly improved in terms of NDVI data processing and description in the previous rounds of revisions.
